# Integrative Molecular Profiling and Single-Cell Characterization of *LHFPL5* Reveal Cancer-Specific Regulatory Features in Clear Cell Renal Cell Carcinoma and Lung Adenocarcinoma

**DOI:** 10.3390/medicina62101971

**Published:** 2026-10-12

**Authors:** Hakeemah H. Al-Nakhle, Zinab O. Doha

**Affiliations:** 1 Department of Clinical Laboratory Sciences, College of Applied Medical Sciences, Taibah University, Madinah 42353, Saudi Arabia; ztoha@taibahu.edu.sa; 2 Health and Life Research Center, Taibah University, Madinah 42353, Saudi Arabia

**Keywords:** *LHFPL5*, clear cell renal cell carcinoma, lung adenocarcinoma, copy-number variation, DNA methylation, transcriptomics, tumor microenvironment, single-cell RNA sequencing, overall survival

## Abstract

*Background and Objectives*: *LHFPL5* encodes a four-pass transmembrane protein essential for cochlear mechanotransduction, but its molecular behavior in cancer remains poorly defined. We characterized the genomic, epigenetic, transcriptomic, immune, and cellular context of *LHFPL5* in clear cell renal cell carcinoma (KIRC) and lung adenocarcinoma (LUAD), along with its associations with clinical outcomes. *Materials and Methods*: Retrospective analyses integrated TCGA RNA sequencing, clinical, DNA methylation, copy number, tumor purity, and immune deconvolution data. Transcriptome-wide associations and Hallmark enrichment defined *LHFPL5*-associated programs. Continuous-expression Cox models estimated hazard ratios (HRs) per standard-deviation increase, adjusted for age, sex, and pathological stage. GEO cohorts assessed external reproducibility, matched TCGA expression pipelines assessed processing sensitivity, and three non-TCGA single-cell datasets examined cellular localization. *Results*: *LHFPL5* showed distinct molecular contexts in KIRC and LUAD. In KIRC, higher expression remained associated with poorer overall survival after clinical adjustment (528 patients; 174 deaths; HR = 1.36, 95% CI 1.21–1.53; *p* = 2.42 × 10^−7^), while subtype and immune fractions showed limited statistical overlap with the association. In LUAD, expression covaried with copy-number state and showed a weak inverse relationship with first-exon methylation. Proliferation-related transcriptional programs were associated with survival, although enrichment support depended on the multiple-testing procedure. The primary STAR-derived LUAD analysis was null (485 patients; 177 deaths; HR = 1.02, 95% CI 0.89–1.18; *p* = 0.770), and survival inference varied with RNA processing. External adjusted replication was inconclusive. Single-cell detection was sparse in KIRC and one LUAD dataset; another LUAD cohort showed more frequent epithelial detection without resolved malignant-cell identity. *Conclusions*: *LHFPL5* exhibits cancer-specific molecular architecture. LUAD showed copy-number, methylation, and proliferation-related associations, whereas KIRC showed a distinct molecular context with an independent survival signal. These findings support context-dependent *LHFPL5* regulation and prioritize genomic, epigenetic, and transcriptional mechanisms for functional investigation.

## 1. Introduction

*LHFPL5* (TMHS) encodes a four-pass transmembrane protein best characterized as a component of the cochlear mechanotransduction complex. In sensory hair cells, *LHFPL5* interacts with PCDH15 and contributes to the structural coupling required for efficient transmission of mechanical force to the mechanosensory apparatus [1,2]. These functions establish *LHFPL5* as a specialized membrane-associated protein, but whether comparable molecular properties are relevant in cancer remains unknown. Its membrane topology alone does not support classification as a conventional tetraspanin or imply a direct role in tumor-cell migration, invasion, or signaling.

Observations involving other members of the LHFPL family provide a rationale for examining this protein family in cancer, but they cannot be extrapolated directly to *LHFPL5*. *LHFPL2* has been associated with macrophage-related phenotypes in renal carcinoma, whereas *LHFPL6* has been investigated in gastric cancer [3,4]. Because these findings involve distinct proteins and biological settings, they offer limited mechanistic insight into *LHFPL5*. Our focus on clear cell renal cell carcinoma (KIRC) and lung adenocarcinoma (LUAD) instead arose from an earlier pan-cancer screen in which higher *LHFPL5* expression showed adverse overall-survival signals in these two cohorts. This observation provided the basis for a more focused evaluation of the molecular features accompanying *LHFPL5* expression.

Such analysis is particularly relevant in KIRC and LUAD because both tumors are characterized by substantial molecular and cellular heterogeneity. Bulk RNA measurements may reflect not only tumor-cell transcription but also variation in immune and stromal composition, genomic dosage, epigenetic state, and technical differences in RNA processing. Associations with clinical outcome may likewise be influenced by age, pathological stage, molecular subtype, smoking exposure, tumor purity, and other cohort-specific factors [5,6,7]. Similarly, parallel changes in DNA methylation and gene expression do not by themselves establish epigenetic regulation and require locus-specific assessment in matched samples [8]. Integrating transcriptomic, genomic, epigenetic, immune, and single-cell data therefore provides a framework for distinguishing cancer-associated molecular patterns from those attributable to tissue composition or analytical variation.

In the present study, the initial pan-cancer findings were used only to select KIRC and LUAD for focused investigation and were not treated as confirmatory evidence. We examined whether continuous *LHFPL5* expression remained associated with overall survival after adjustment for major clinical and tumor-composition variables and characterized its molecular context through transcriptome-wide association analysis, Hallmark pathway enrichment, immune-cell profiling, DNA methylation, and copy-number analysis. External cohorts were used to assess prognostic reproducibility, while matched RNA-processing comparisons evaluated analytical sensitivity. Non-TCGA single-cell datasets were further examined to define the cellular distribution of LHFPL5. KIRC and LUAD were analyzed separately, without assuming a shared mechanism, to delineate cancer-specific molecular features and identify regulatory hypotheses for subsequent functional investigation.

## 2. Materials and Methods

### 2.1. Study Design, Initial Pan-Cancer Screening, and Focused Cohort Assembly

The study was conducted in two stages. First, an exploratory pan-cancer bioinformatics screen was performed to characterize *LHFPL5* expression across human malignancies and identify cancer types warranting more detailed investigation. Transcriptomic data and corresponding clinical annotations for more than 10,000 tumor and normal samples spanning 33 cancer types were obtained primarily from The Cancer Genome Atlas (TCGA; accessed 20 May 2025) and the Genotype-Tissue Expression project (GTEx; accessed 20 May 2025). Tumor samples and available adjacent or healthy normal tissues were included. Samples lacking *LHFPL5* expression measurements or the clinical variables required for a given analysis were excluded from that specific analysis. Expression values were analyzed as transcripts per million (TPM) or RNA-Seq by Expectation Maximization (RSEM)-normalized values, depending on the source platform, with logarithmic transformation applied where appropriate. The initial screening analyses were performed using the browser-based platform versions available on the stated access dates.

The pan-cancer screen identified adverse overall-survival signals associated with higher *LHFPL5* expression in clear cell renal cell carcinoma (KIRC) and lung adenocarcinoma (LUAD). These observations were used solely to prioritize KIRC and LUAD for subsequent patient-level analyses and were not treated as independent prognostic validation. The second stage therefore consisted of a retrospective, focused reanalysis of these two cohorts, designed to reassess the survival associations and characterize the accompanying molecular context. The focused study was not prospectively specified as a prognostic validation analysis.

For the focused reanalysis, data were accessed on 23 September 2026. STAR-derived RNA-sequencing TPM matrices and clinical metadata were obtained from the UCSC Xena GDC hub. The RNA files were supplied as log2(TPM + 1), used GENCODE v36/GRCh38 identifiers, carried the Xena version string “05-10-2024,” and were linked in the accompanying metadata to GDC release 40. No additional logarithmic transformation was applied to these expression values. Overall survival (OS) information was obtained from the TCGA Clinical Data Resource (TCGA-CDR) [7,9].

Only primary tumors were retained for the focused analyses. When more than one primary-tumor barcode was available for the same patient, the lexicographically first sample was selected, regardless of expression level or clinical outcome. Molecular datasets were matched at the patient level using TCGA barcodes after primary-sample selection within each source. Within each cancer, *LHFPL5* expression was standardized using the mean and sample standard deviation calculated across all eligible unique primary tumors, and the same scaling constants were applied across nested models. Complete-case populations were defined separately for each model family; missing covariates were not imputed. Patients with missing, zero, or otherwise invalid OS follow-up were excluded from survival analyses.

Additional patient-matched molecular data included ABSOLUTE tumor-purity estimates, GISTIC copy-number measurements, published CIBERSORT relative immune-cell fractions, DNA methylation-derived leukocyte fractions, mRNA subtype annotations, and Bindea immune-signature scores obtained from GDC-hosted Pan-Cancer Atlas resources. Individual Illumina HumanMethylation450 probes were retrieved from the legacy Xena TCGA hub and verified against GPL13534 annotations (GRCh37) [6,10,11,12,13]. Because the RNA-sequencing and methylation resources were based on different genome assemblies, genomic coordinates were not directly intersected; integration instead relied on verified gene or probe identifiers and patient barcodes. Data provenance, sample-flow information, model-specific populations, and resource versions are detailed in Supplementary Tables S1 and S12. Patient-level matching should not be interpreted as confirmation that all molecular measurements were generated from the same tissue aliquot. All analyses used existing datasets and involved no participant recruitment, intervention, or new biospecimen collection. OS, defined as death from any cause, was the only clinical endpoint retained in the focused survival analysis.

### 2.2. LHFPL5 Expression Profiling Across Cancer Types

*LHFPL5* identity was first confirmed using NCBI Gene. Expression data for *LHFPL5* (TMHS; Entrez Gene 222662; ENSG00000197753.8) were then obtained from the UCSC Xena Browser Toil dataset TcgaTargetGtex_rsem_gene_tpm (version 2016-09-03), accessed on 25 September 2026. This dataset provides uniformly processed TCGA and GTEx RNA-sequencing data generated using the STAR–RSEM pipeline. The deposited log_2_(TPM + 0.001) values were used directly for visualization without additional transformation, whereas TPM values were reconstructed for descriptive statistics and fold-change calculations.

Primary TCGA tumors and available adjacent-normal tissues were included, with one sample retained per participant within each tissue group. Comparisons between TCGA tumors and adjacent-normal tissues were performed separately from analyses incorporating anatomically matched GTEx normal tissues. When multiple GTEx samples were available from the same donor, expression values were averaged at the donor level. No alternative normal tissue was used when an appropriate anatomical comparator was unavailable.

Differences in *LHFPL5* expression between independent groups were assessed using two-sided Mann–Whitney U tests, with a minimum of five samples required in each group. Comparisons based on 5–9 normal samples were considered exploratory. Effect sizes were summarized using Cliff’s delta together with the ratio of arithmetic mean TPM values. For KIRC and LUAD, matched tumor–adjacent-normal pairs were additionally evaluated using Wilcoxon signed-rank tests after excluding pairs with zero differences. Rank-based tests used asymptotic *p*-values with continuity correction. Multiple-testing correction was performed using the Benjamini–Hochberg procedure separately for the 18 TCGA tumor–adjacent-normal comparisons, 23 TCGA/GTEx comparisons, and two paired analyses, with an FDR < 0.05 considered statistically significant. Sensitivity analyses were also conducted after excluding tumors from participants who contributed adjacent-normal tissue.

Associations between *LHFPL5* expression and clinicopathological features were examined using clinical annotations from the UCSC Xena TCGA matrices (version 2019-12-06). Kruskal–Wallis tests were used to assess differences according to pathological stage, KIRC tumor grade, and LUAD smoking history, whereas sex-based differences were evaluated using Mann–Whitney U tests. Associations with age were examined using Spearman’s rank correlation. Samples lacking the clinical information required for a given analysis were excluded, and Benjamini–Hochberg correction was applied across the eight clinical tests. For additional cross-platform comparison, results reported by the UALCAN portal for seven cancer types were examined. These analyses were considered consistency checks rather than independent validation because UALCAN is also based largely on TCGA data.

### *LHFPL5* Expression and Survival in Clear Cell Renal Cell Carcinoma and Lung Adenocarcinoma

The association between *LHFPL5* expression and survival was examined using TCGA PanCancer Atlas data obtained from cBioPortal for Cancer Genomics (https://www.cbioportal.org/; accessed on 25 September 2026). This exploratory analysis was performed to provide context for the subsequent focused investigation of KIRC and LUAD. Expression and clinical data were retrieved for 14 of the 33 TCGA cancer types. Batch-normalized Illumina HiSeq RNASeqV2 RSEM expression values were used as deposited, without additional normalization or logarithmic transformation. One tumor sample was retained per patient, giving priority to primary tumors and using sample identifiers to resolve remaining duplicates. Within each cohort, patients were assigned to high-expression (>median) or low-expression (≤median) groups. The median was calculated before exclusions for missing outcomes and was held constant for both endpoints. Tied values, including zeros, were retained in the low-expression group. No outcome-based cutoff optimization was performed. The cutoffs were 0.3108 RSEM units for KIRC and 1.86105 for LUAD, each derived from 510 patients with expression data.

Overall survival (OS) was defined as the primary endpoint and disease-free survival (DFS) as the secondary endpoint. OS was derived from the deposited OS_MONTHS and OS_STATUS fields, with death considered an event, whereas DFS was based on DFS_MONTHS and DFS_STATUS, with recurrence or progression considered an event and disease-free status treated as censored, according to cBioPortal definitions (https://docs.cbioportal.org/file-formats/, accessed on 25 September 2026). Death was not independently incorporated into the DFS definition. Patients with missing or invalid survival data were excluded separately for each endpoint, without imputation.

Kaplan–Meier curves and two-sided log-rank tests were generated from the downloaded data. Unadjusted Cox models were used to estimate hazard ratios (HRs) with 95% confidence intervals for high versus low *LHFPL5* expression using lifelines v0.30.3 (https://lifelines.readthedocs.io/). Nominal *p* < 0.05 was considered exploratory, and Benjamini–Hochberg correction was applied separately to the 14 OS and 13 DFS comparisons. These median-based analyses were used for screening and were considered complementary to the continuous-expression multivariable Cox models used in the focused KIRC and LUAD analyses.

### 2.3. Clinical Outcome Modeling and Sensitivity Analyses

The primary exposure was continuous standardized *LHFPL5* expression, with hazard ratios (HRs) expressed per 1-SD increase. Cox proportional-hazards regression with Efron handling of tied events was used for survival modeling. Model 0 included *LHFPL5* expression alone; Model 1 additionally adjusted for age, entered per 10-year increment, and sex; Model 2 further incorporated pathological stage, with stages II–IV compared with stage I; and Model 3 added continuous ABSOLUTE tumor purity. Models 0–2 were evaluated within the same clinical complete-case population, whereas Models 0–3 were compared within the corresponding purity-complete subset. A maximal univariable analysis was retained separately. Cancer-specific sensitivity analyses additionally considered tumor grade in KIRC (III/IV versus I/II), KIRC mRNA subtype, and smoking exposure in LUAD, modeled per 20 pack-years, when sufficient data and events were available. More extensively adjusted models were fitted only when the event count supported approximately ten deaths per estimated coefficient.

The proportional-hazards assumption was assessed using scaled Schoenfeld residuals, covariate-specific rank-time tests, and a joint Efron risk-set score test for time interactions, together with graphical diagnostics. When categorical covariates showed evidence of nonproportionality, we examined stage- and sex-stratified models. Residual concerns were explored using counting-process models in which selected coefficients were allowed to differ before and after three years. Potential nonlinearity and influence were evaluated using natural cubic splines with three degrees of freedom, quadratic terms, and sensitivity analyses excluding the highest expression percentile. Primary Cox estimates were independently reproduced using statsmodels. Kaplan–Meier analyses were performed as secondary descriptive analyses using the cohort median of *LHFPL5* expression across unique eligible primary tumors; values above the median defined the high-expression group, and no data-driven or optimized cutpoint was applied.

For each Cox model, we reported the model-specific sample size, number of deaths, HR, 95% Wald confidence interval, and two-sided Wald *p* value. Log-rank *p* values were restricted to Kaplan–Meier comparisons. Dichotomized high-versus-low Cox estimates were treated as secondary sensitivity analyses and are presented separately in Supplementary Table S2. Reliable treatment data and LUAD molecular-subtype annotations were unavailable; therefore, we could not assess their potential confounding effects.

### 2.4. Clinicopathological and Molecular Subtype Analyses

Associations between *LHFPL5* expression and clinicopathological features were examined using Kruskal–Wallis tests. These analyses included pathological stage in both cohorts, as well as tumor grade and molecular subtype in KIRC. For each comparison, group sizes, median expression values, and epsilon-squared effect sizes were reported. The four global tests were considered a single multiple-testing family and adjusted using the BH procedure. Pairwise comparisons between KIRC molecular subtypes were undertaken only when the corresponding global test was significant, with BH correction applied across the six pairwise contrasts. Changes in the *LHFPL5* survival coefficient after adjustment for stage, grade, or molecular subtype were interpreted as evidence of statistical overlap between these variables and the expression–survival association, rather than as evidence of causal mediation.

### 2.5. Transcriptome-Wide Association and Hallmark Enrichment

Within each cancer type, gene-level log2(TPM + 1) expression was modeled against standardized LHFPL5 expression while adjusting for age, sex, pathological stage, and tumor purity. Ordinary least-squares regression with HC3 heteroskedasticity-robust standard errors was used. Genes were included if TPM was ≥1 in at least 20% of the corresponding modeling population. *LHFPL5* was excluded from the ranked gene list to avoid self-association. Gene-level significance was assessed using two-sided *t*-tests with residual degrees of freedom, and Benjamini–Hochberg correction was applied separately within each cancer. These models were designed to identify transcriptomic features independently associated with *LHFPL5* expression after covariate adjustment and were not interpreted as evidence of direct transcriptional regulation.

For comparison, an unadjusted transcriptome-wide Spearman analysis was also performed using all unique primary tumors, comprising 533 KIRC and 516 LUAD samples. Multiple testing was controlled separately within each cancer using the Benjamini–Hochberg procedure. These correlation results are reported in Supplementary Table S3 and were kept analytically distinct from the clinically and purity-adjusted gene-level models used for downstream enrichment analyses.

Preranked gene-set enrichment analysis was performed using the MSigDB Hallmark collection, release 2026.1.Hs, with signed HC3 t-statistics as the ranking metric. Analyses were conducted in GSEApy v1.3.1 using 10,000 gene-set permutations, a fixed random seed of 20260923, and gene-set size limits of 15–500 genes [14,15,16,17]. When duplicate gene symbols were present, we retained the entry with the highest mean expression, regardless of statistical significance. All 50 Hallmark gene sets were evaluated. Under the archived two-sided reporting procedure, directional nominal *p* values were doubled, capped at 1, assigned a minimum permutation-tail probability of 1/10,001, and subsequently adjusted across the 50 tested sets within each cancer using the Benjamini–Hochberg method. This implementation differed from the initially proposed absolute-NES permutation framework.

### 2.6. Pathway Scores and Outcome Integration

Prior to conducting survival analyses, up to three Hallmark pathways per cancer type were selected based on the absolute normalized enrichment score (NES) among gene sets that met the criterion of a BH-adjusted *q*-value less than 0.05. This methodology resulted in the identification of two pathways in KIRC and three pathways in LUAD. Single-sample GSEA (ssGSEA) scores were subsequently derived from within-sample expression ranks and standardized prior to downstream analyses. Associations between pathway scores and *LHFPL5* expression levels were evaluated using Spearman correlation coefficients. Survival analyses were performed employing both pathway-only Cox proportional hazards models and combined models that incorporated *LHFPL5* expression, with adjustments for age, sex, pathological stage, and tumor purity. All comparisons were conducted within identical complete-case populations to ensure consistency across all models. Multiple testing correction was applied within each cancer-specific pathway family through the Benjamini–Hochberg procedure. Given that preranked gene-set enrichment analysis and sample-level ssGSEA correlations assess different analytical features, any discordances observed between these measures were interpreted separately, rather than assuming they reflect the same underlying biological phenomenon.

### 2.7. Tumor Purity and Immune-Cell Composition

Associations between *LHFPL5* expression and immune-cell composition were assessed after adjusting for tumor purity. Both *LHFPL5* expression levels and immune-cell fractions were residualized against ranked ABSOLUTE purity, and partial Spearman correlation analyses were performed on the resultant residuals. Two-sided significance tests along with approximate Fisher z-based confidence intervals were reported. Multiple testing corrections within each cancer type employed the BH procedure across 22 CIBERSORT cell fractions and the DNA-derived leukocyte fraction. As a sensitivity analysis of quality, the same associations were also examined within the subset of samples with CIBERSORT, *p* < 0.05.

The published Bindea immune-signature scores were analyzed as a complementary 25-signature framework, with multiple-testing correction performed separately within each cancer [6,18]. Since these scores were derived from the same TCGA patients, they were not regarded as an independent cohort. For subsequent survival analyses, the two most significant FDR-primary immune associations in each cancer were selected prior to outcome modeling. Feature-only and combined Cox models were fitted using identical patient cohorts, and survival tests for immune features were corrected within their respective model families. A validated stromal-score measure was not available for the present analysis.

### 2.8. DNA Methylation, Copy Number, and Integrated Expression Models

Eleven candidate DNA methylation probes identified through the *LHFPL5* Xena mapping were evaluated. Ten matched the original *LHFPL5* manifest annotation, while one annotation-discordant probe was retained for transparency and audit purposes. For each probe, the identifier, chromosome, GRCh37 genomic position, gene-region annotation, and CpG-island context were recorded. Methylation–expression associations were assessed by Spearman correlation in patient-matched primary tumors, with BH correction applied across the 11-probe candidate set. HC3-robust sensitivity regressions further adjusted for copy number, tumor purity, age, sex, and pathological stage. Continuous GISTIC copy ratios were correlated with LHFPL5 expression, whereas categorical copy-number states were compared using Kruskal–Wallis tests.

The primary multiple-testing family retained cg18451957 for completeness despite its annotation discrepancy. As the original manifest assigns this probe to SRPK1, it was excluded from the biological interpretation of LHFPL5. A separate LUAD sensitivity analysis was conducted, wherein the BH correction was reapplied using only the ten probes for *LHFPL5* that were confirmed in the manifest. Results from both testing families, including their adjusted *p*-values, are provided in Supplementary Table S7. Findings whose statistical classification changed according to the correction family were deemed threshold-sensitive rather than robust.

Because no individual promoter probe met the initial False Discovery Rate (FDR) criterion, we constructed an exploratory post hoc promoter summary by calculating the mean of seven transcription-start-site probes as defined by the annotation. The calculation of this summary required that at least 80% of the constituent probes be observed. Standardized *LHFPL5* expression was subsequently modeled as a function of the promoter mean, copy ratio, tumor purity, age, sex, and pathological stage. Confidence intervals for HC3, R^2^, adjusted R^2^, variance inflation factors, residual diagnostics, and incremental R^2^ were determined within the same patient cohort. It is important to note that this promoter-averaging analysis was not selected based on survival outcomes. Methylation variables that were chosen were incorporated into survival models solely when the number of events available was deemed sufficient.

### 2.9. Single-Cell RNA-Sequencing Localization

Cellular localization of *LHFPL5* was examined using three non-TCGA single-cell RNA-sequencing datasets: GSE159115 for ccRCC, GSE131907 for primary LUAD, and KRA accession KAP241022 for primary LUAD [19,20,21]. GSE159115 was represented by two exports derived from the same seven-patient cohort; these were analyzed separately and were not treated as independent replication. The initial focused analysis comprised 20,748 ccRCC cells and 45,149 cells from 11 patients in GSE131907. Curated CELLxGENE exports contained 20,509 ccRCC cells from the same seven GSE159115 patients and 117,266 cells from 18 treatment-naive patients in KAP241022. Benign kidney tissue, chromophobe renal cell carcinoma, and renal metastases identified on the basis of anatomical site were excluded from the ccRCC/LUAD comparison. Dataset- and export-specific denominators are detailed in Supplementary Table S9.

For the initial focused analysis, raw UMI counts were normalized to 10,000 counts per cell and log1p-transformed. Published annotations for tumor, tumor-associated epithelial, immune, vascular, and other cellular compartments were retained. Cells were considered *LHFPL5*-positive when at least one UMI was detected. Patient-level paired compartment comparisons were attempted only when prespecified eligibility criteria were met: at least five evaluable patient pairs, adequate numbers of cells within the compared compartments, at least 20 positive cells across the comparison, and at least five nonzero paired differences. Two-sided paired Wilcoxon tests were reported only for comparisons satisfying all of these criteria.

A subsequent localization analysis used the authors’ original quality-control procedures, annotations, and embeddings from curated exports accessed on 24 September 2026. Raw integer UMI counts were extracted from raw. X for ccRCC and X for LUAD, with *LHFPL5* uniquely mapped to ENSG00000197753. Normalized expression was calculated as ln[1 + 10,000 × LHFPL5 UMI/total exported UMI per cell]. All exported cells were retained, with no additional clustering, imputation, batch correction, or ambient-RNA correction. Detection at >0 UMI and, as a sensitivity threshold, ≥2 UMIs was summarized by annotated cell population and donor. Kidney t-SNE and lung UMAP coordinates were taken directly from the original studies and were interpreted only within their respective datasets.

Although the KAP241022 study performed malignancy inference, per-cell malignancy assignments were not included in the analyzed export; therefore, epithelial cells were not reclassified as malignant. Analyses based on the curated exports were descriptive and did not involve cell-level significance testing. In this study, “non-TCGA” refers only to the origin of the single-cell datasets relative to the TCGA bulk cohorts. Single-cell localization was not interpreted as validation of bulk differential expression, survival, methylation, immune associations, or drug response.

### 2.10. External Cohorts and RNA-Processing Sensitivity

External overall-survival analyses were performed using GSE29609 for KIRC (GPL1708 probe 41466/A_32_P494620) and GSE72094 for LUAD (GPL15048 probe merck-NM_182548_at) [22,23]. GSE68465 was excluded before outcome modeling because its GPL96 annotation contained no match for *LHFPL5*, TMHS, Entrez 222662, or NM_182548 [24,25]. Probe-sequence reannotation was not attempted. Deposited normalized expression values were used as provided, without applying an additional logarithmic transformation, and were standardized across all measured samples within each cohort. Cox models used Efron handling of tied events and reported 95% Wald confidence intervals with two-sided nominal *p* values. Age, sex, and categorical stage were included when available and when the prespecified convention of approximately ten deaths per model coefficient was satisfied. This event-count criterion was used as a modeling safeguard and was not interpreted as evidence of adequate statistical power.

Proportional-hazards assumptions in the external cohorts were assessed using scaled Schoenfeld rank tests and a joint Efron rank-time score test. Stratified models were used to examine departures associated with categorical covariates. GSE29609 included only 17 deaths and lacked deposited sex and directly usable overall-stage information. The available event count was insufficient even for the planned age-adjusted model; accordingly, no adjusted estimate was fitted. Complete-case and stage-stratified analyses for GSE72094 are reported in Supplementary Table S10.

Sensitivity to RNA-processing methodology was assessed by matching TCGA STAR-derived log2(TPM + 1) expression values with legacy Xena log2(RSEM normalized count + 1) values by patient and primary-tumor sample type. Expression concordance was quantified using Spearman and Pearson correlations together with agreement in median-based expression groups. Each expression source was standardized within the same matched patient population before fitting otherwise identical Cox models. Legacy identifiers did not permit confirmation of vial- or aliquot-level identity. These comparisons therefore reflect the combined influence of quantification, annotation, processing, and possible specimen differences rather than the effect of a single alignment method. Because the analyses reuse the same TCGA patients, they were interpreted as processing-sensitivity analyses rather than external replication (Supplementary Table S11).

### 2.11. Statistical Framework and Reproducibility

All statistical tests were two-sided. BH correction families were defined according to analysis domain and cancer, with the exception of the prespecified four-test severity and copy-number families and the two-test purity family. Prespecified single-exposure coefficients and proportional-hazards diagnostic tests were not subjected to additional FDR correction. Because selection of KIRC and LUAD, as well as subsequent prioritization of molecular features, arose from an exploratory discovery framework, pathway, immune, methylation, and regulatory analyses were considered hypothesis-generating. Although feature-selection files were finalized before downstream feature-survival modeling, this sequencing does not remove the potential for same-cohort selection bias.

Analyses were performed using Python 3.12.14, lifelines 0.30.3, statsmodels 0.15.0, SciPy 1.18.1, and GSEApy 1.3.1. Executed scripts, package and session information, analysis-ready data extracts, source records, patient-level model membership, and complete statistical outputs are provided in Supplementary Tables S1–S12 and the accompanying reproducibility files.

## 3. Results

### 3.1. Pan-Cancer Expression Profile of LHFPL5

**Figure 1:**
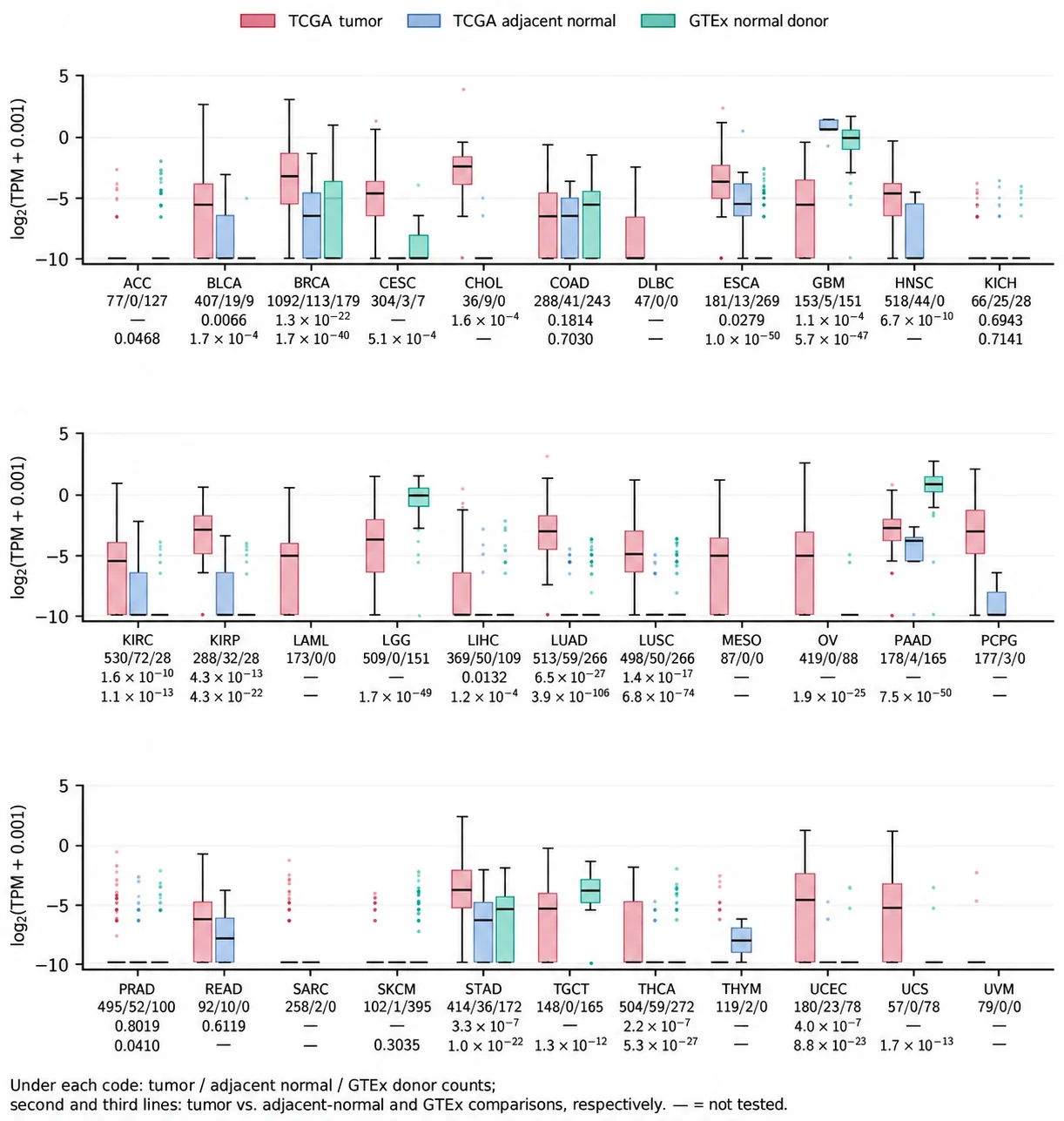
Pan-cancer expression of *LHFPL5*. Expression levels are shown for TCGA tumors, TCGA adjacent-normal tissues, and selected GTEx normal donors. Sample sizes and two-sided Mann–Whitney *p* values are indicated for tumor–adjacent-normal and tumor–pooled-normal comparisons. Boxes represent the median and interquartile range (IQR), whiskers extend to 1.5 × IQR, and individual points indicate outliers. Expression values are presented on the deposited log_2_(TPM + 0.001) scale.

**Figure 2:**
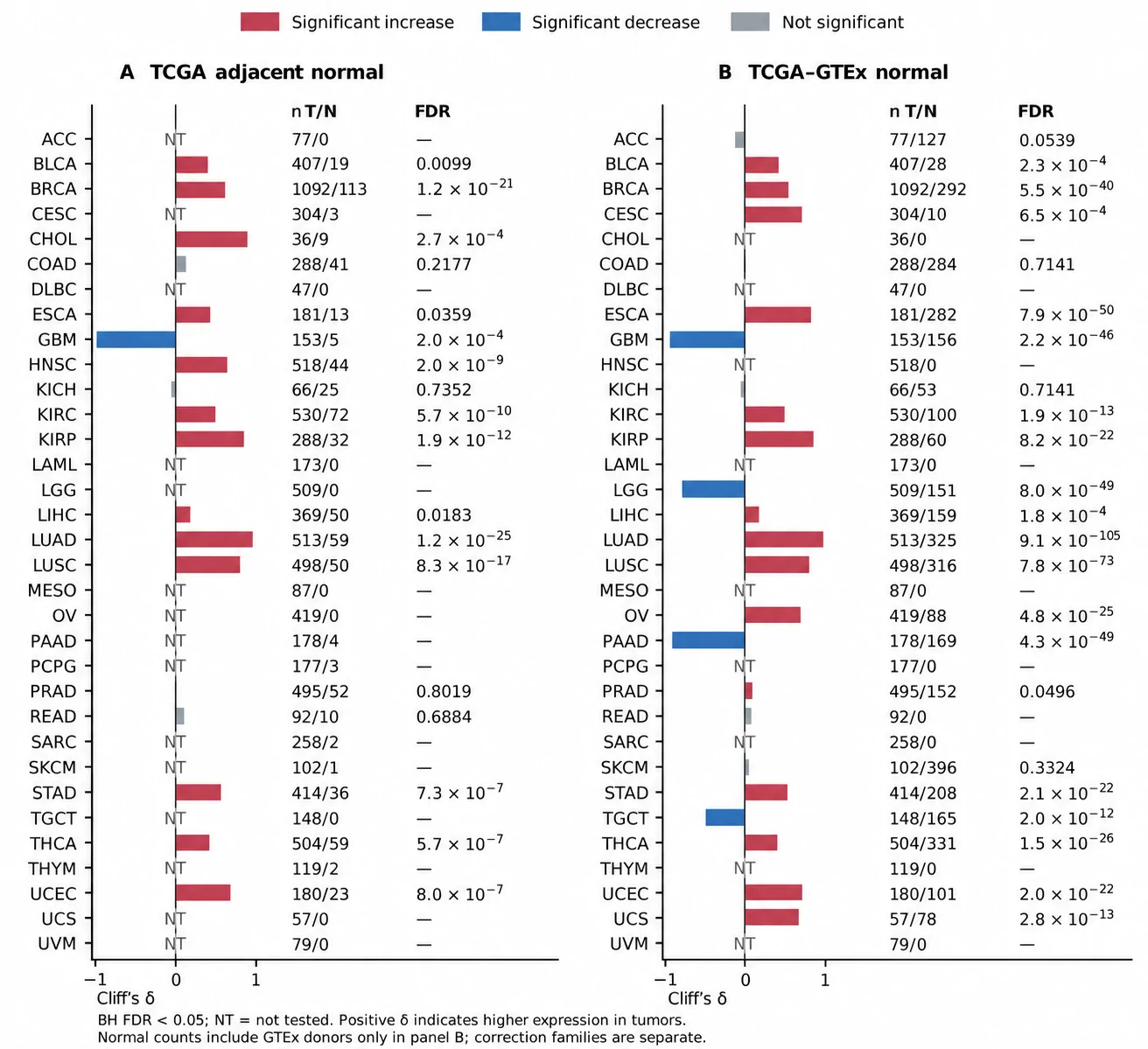
Differential *LHFPL5* expression across cancer types. Cliff’s delta shows the direction and magnitude of tumor–normal differences. Significant changes were defined at BH FDR < 0.05; gray indicates non-significance. Sample sizes and FDR values are shown. NT indicates an unavailable or insufficient comparator.

Across 33 TCGA cancer types, 9358 primary tumors and 727 adjacent-normal samples were included. Of the 18 cancers eligible for adjacent-normal testing, 13 showed higher *LHFPL5* expression and one, GBM, showed lower expression after FDR correction (Figure 1 and Figure 2). Four showed no significant difference; 15 lacked sufficient adjacent normals. These classifications were unchanged in donor-disjoint analyses. The GBM and CHOL comparisons were exploratory because they included only five and nine normals, respectively. Separately, harmonized TCGA–GTEx comparisons identified 15 increases and four decreases among 23 evaluable cancers. These findings depend on the selected normal tissues and should be distinguished from the adjacent-normal results.

**Figure 3:**
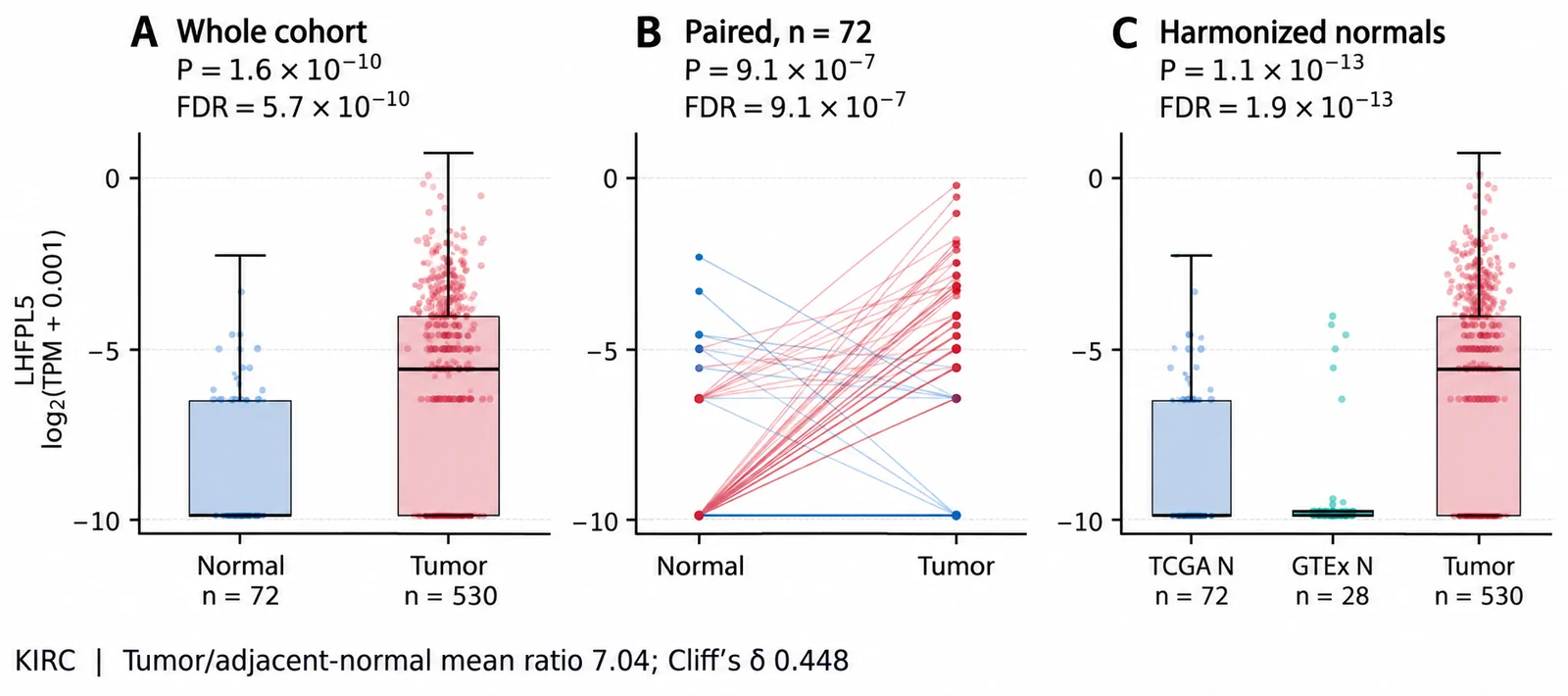
*LHFPL5* expression in KIRC. (**A**) Tumors versus adjacent normals. (**B**) Matched tumor–normal samples from 72 patients. (**C**) Normal sources shown separately, with 100 pooled normal samples including 28 GTEx kidney-cortex donors. Mann–Whitney tests were used for A and C and Wilcoxon testing for B, with FDR correction applied within the respective comparison families. Boxes show medians and IQRs; points represent individual samples.

In KIRC, *LHFPL5* expression was higher in 530 tumors than in 72 adjacent normal samples. Median expression was 0.0200 TPM (IQR 0–0.0600) versus 0 TPM (IQR 0–0.0100). Mean expression was 0.0596 versus 0.0085 TPM, corresponding to a 7.04-fold difference (Cliff’s delta = 0.448; *p* = 1.5755 × 10^−10^; FDR = 5.6716 × 10^−10^). The difference persisted in 72 matched pairs (median increase, 0.0200 TPM; FDR = 9.0862 × 10^−7^) and with harmonized normals (Figure 3).

**Figure 4:**
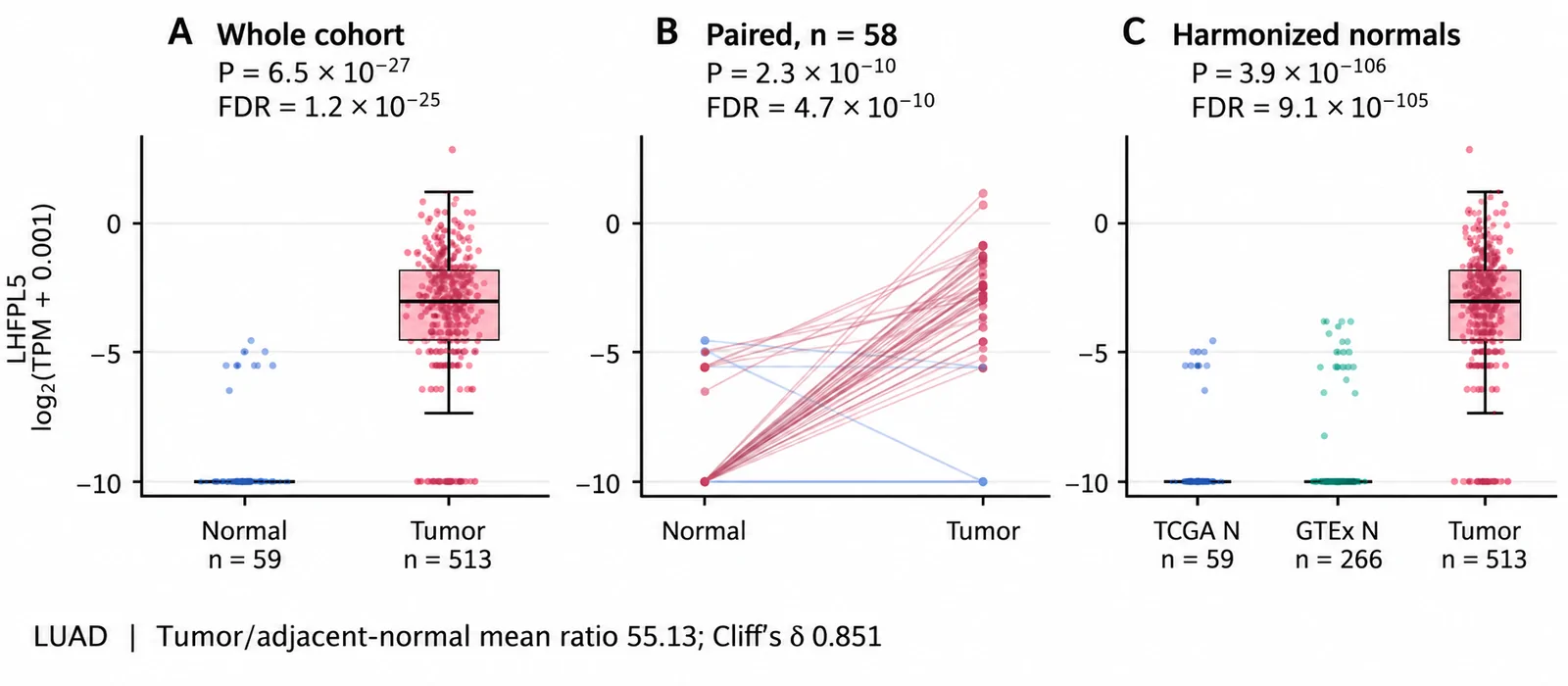
*LHFPL5* expression in LUAD. (**A**) Tumors versus adjacent normals. (**B**) Matched tumor–normal samples from 58 patients. (**C**) Pooled normal samples (n = 325), including 266 GTEx lung donors. One adjacent-normal donor lacked a corresponding primary tumor. Statistical tests and plot elements are as described in Figure 3, with FDR correction applied within the respective comparison families.

In LUAD, expression was higher in 513 tumors than in 59 adjacent normals. Median expression was 0.1200 TPM (IQR 0.0400–0.2900) versus 0 TPM (IQR 0–0). Mean expression was 0.2429 versus 0.0044 TPM, a 55.13-fold difference (Cliff’s delta = 0.851; *p* = 6.5187 × 10^−27^; FDR = 1.1734 × 10^−25^). Paired analysis of 58 patients confirmed higher tumor expression (median increase, 0.1400 TPM; FDR = 4.6509 × 10^−10^), as did the harmonized comparison (Figure 4).

**Figure 5:**
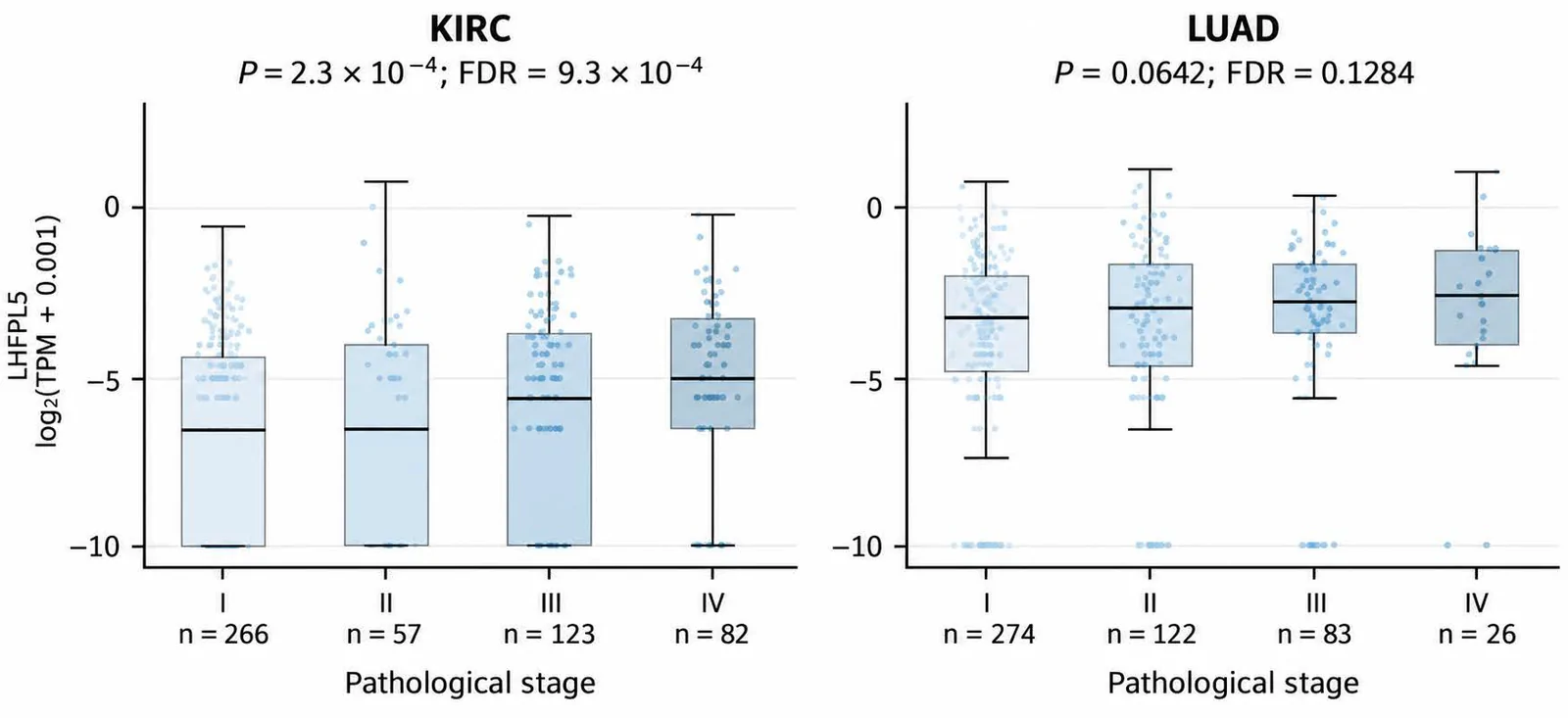
*LHFPL5* expression across pathological stages. Expression was compared across stages I–IV using Kruskal–Wallis tests, with FDR correction applied across eight clinicopathological analyses. Stage information was available for 528 KIRC and 505 LUAD tumors. Boxes show medians and IQRs, whiskers extend to 1.5 × IQR, and points represent individual tumors.

After correction, KIRC expression differed by stage, grade, and sex, but showed no significant association with age. Stage I–IV medians were 0.0100, 0.0100, 0.0200, and 0.0300 TPM, respectively (FDR = 0.000933). In LUAD, stage, sex, age, and smoking history showed no significant association (Figure 5). These analyses were univariable. UALCAN showed concordant tumor-associated increases in KIRC (533 tumors/72 normals; *p* = 1.7589 × 10^−7^) and LUAD (515/59; *p* = 2.1150 × 10^−13^), supporting cross-platform consistency.

### 3.2. Higher LHFPL5 Expression Is Associated with Poorer Overall Survival in KIRC and LUAD

**Figure 6:**
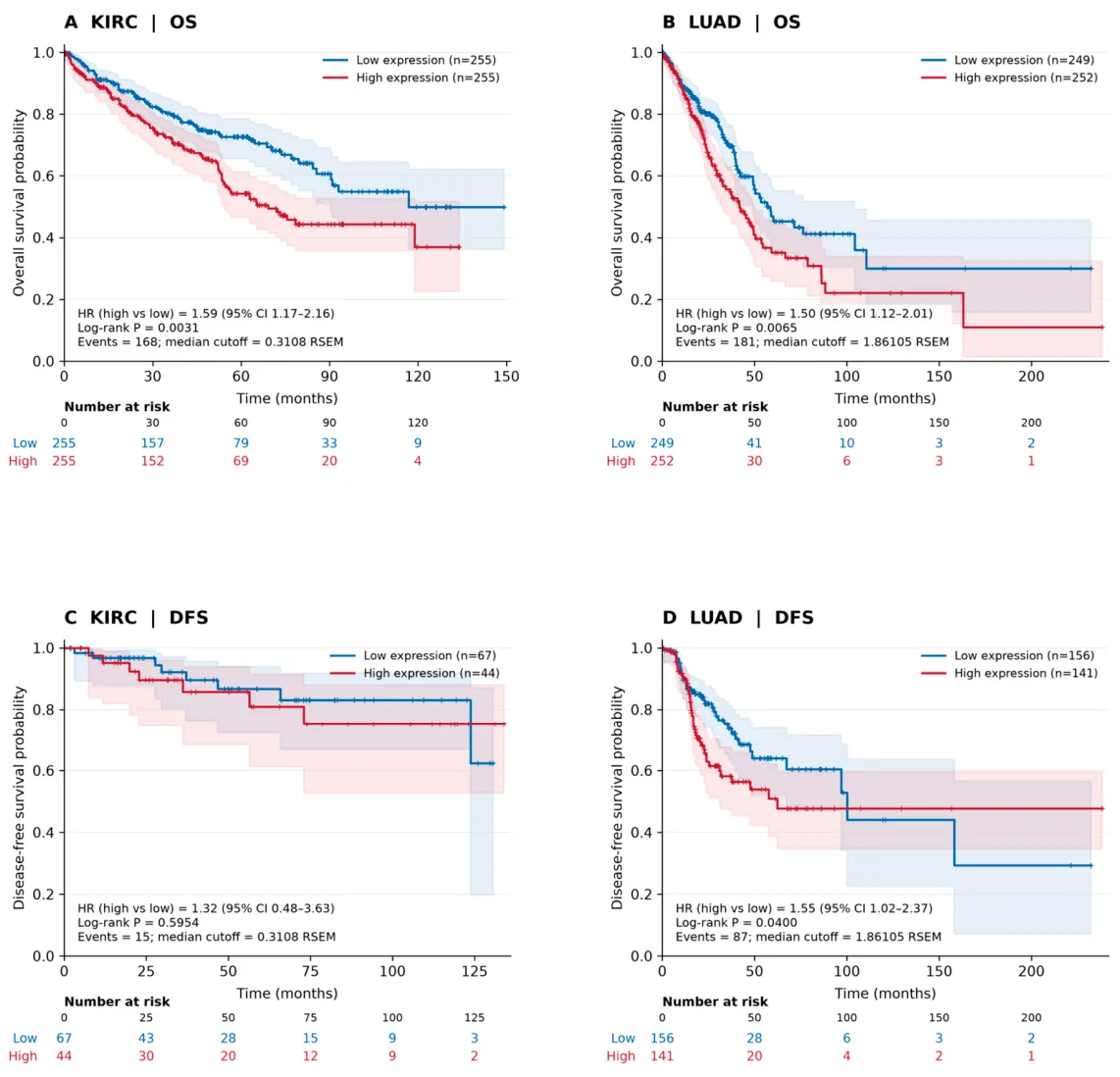
Association of *LHFPL5* expression with overall and disease-free survival in KIRC and LUAD. (**A**) KIRC OS; (**B**) LUAD OS; (**C**) KIRC DFS; and (**D**) LUAD DFS. Patients were stratified into high- and low-expression groups using cohort-specific median *LHFPL5* expression values (0.3108 RSEM for KIRC and 1.86105 RSEM for LUAD). Values above the median were classified as high expression, and those at or below the median as low expression. Shaded areas represent 95% confidence intervals, crosses indicate censored observations, and risk tables show the number of patients at risk. Hazard ratios and 95% CIs were estimated using unadjusted Cox models, while *p* values were obtained from two-sided log-rank tests. Estimates at later follow-up times should be interpreted cautiously because of the smaller numbers at risk. DFS was defined according to the deposited recurrence/progression endpoint, without independently including death as an event.

Higher *LHFPL5* expression was associated with poorer OS in both KIRC and LUAD (Figure 6A,B). In KIRC, 510 patients contributed 168 deaths; the high-expression group had an HR of 1.59 (95% CI 1.17–2.16; log-rank *p* = 0.0031). In LUAD, 501 patients contributed 181 deaths, with an HR of 1.50 (95% CI 1.12–2.01; *p* = 0.0065). Both associations remained significant after correction within the available OS screen (*q* = 0.0216 and 0.0305, respectively).

The DFS findings were less consistent (Figure 6C,D). In KIRC, only 111 patients had eligible DFS records, with 15 events, and the association was not significant (HR 1.32, 95% CI 0.48–3.63; *p* = 0.5954). In LUAD, higher expression was associated with shorter DFS among 297 patients with 87 events (HR 1.55, 95% CI 1.02–2.37; *p* = 0.0400), but this association did not remain significant after correction (*q* = 0.1733).

### 3.3. Patient Cohort Assembly and Molecular Data Harmonization

The RNA-sequencing matrices initially comprised 610 KIRC and 589 LUAD samples. After restricting the analysis to primary tumors and removing repeated samples from the same patient, 533 unique KIRC and 516 unique LUAD patients remained. Valid overall-survival data were available for 531 KIRC patients, including 175 deaths, and 503 LUAD patients, including 182 deaths. The clinical complete-case populations included 528 KIRC patients with 174 deaths and 485 LUAD patients with 177 deaths. After further restriction to patients with available ABSOLUTE tumor-purity estimates, the corresponding subsets comprised 324 KIRC patients with 87 deaths and 462 LUAD patients with 164 deaths (Table 1; Supplementary Table S1). The smaller KIRC purity-complete population reflects additional complete-case filtering and should not be interpreted as a separate cohort.

No duplicate patients remained after cohort assembly. Samples from different molecular data sources were matched using the first 12 characters of the TCGA barcode after primary-tumor selection within each dataset. This procedure establishes patient-level correspondence but does not imply that all molecular measurements were obtained from the same tissue aliquot. Each RNA matrix contained 60,660 gene identifiers, and *LHFPL5* was verified as ENSG00000197753.11 (Entrez 222662). Back-transformation of the deposited log2(TPM + 1) values produced approximately 1,000,000 total TPM per sample, consistent with TPM-normalized expression and confirming that an additional logarithmic transformation was not required. Additional quality-control checks included verifying methylation beta-value ranges, single-cell barcode correspondence, and source-specific library totals. Complete audit records are provided in Supplementary Table S12.

### 3.4. Clinical Association of LHFPL5 Expression

In KIRC, higher *LHFPL5* expression was consistently associated with poorer overall survival. In the clinical complete-case population, the univariable HR per 1-SD increase in expression was 1.27 (95% CI 1.15–1.41; *p* = 3.98 × 10^−6^). After adjustment for age, sex, and pathological stage, the association remained evident and increased slightly in magnitude (HR = 1.36, 95% CI 1.21–1.53; *p* = 2.42 × 10^−7^). Within the purity-complete subset, the clinically adjusted estimate was HR = 1.55 (95% CI 1.28–1.87; *p* = 5.08 × 10^−6^) and was essentially unchanged after inclusion of ABSOLUTE tumor purity (HR = 1.56, 95% CI 1.28–1.91; *p* = 1.01 × 10^−5^; 324 patients, 87 deaths). Thus, the observed KIRC association was not attenuated by adjustment for measured stage or tumor purity in these nested models.

**Figure 7:**
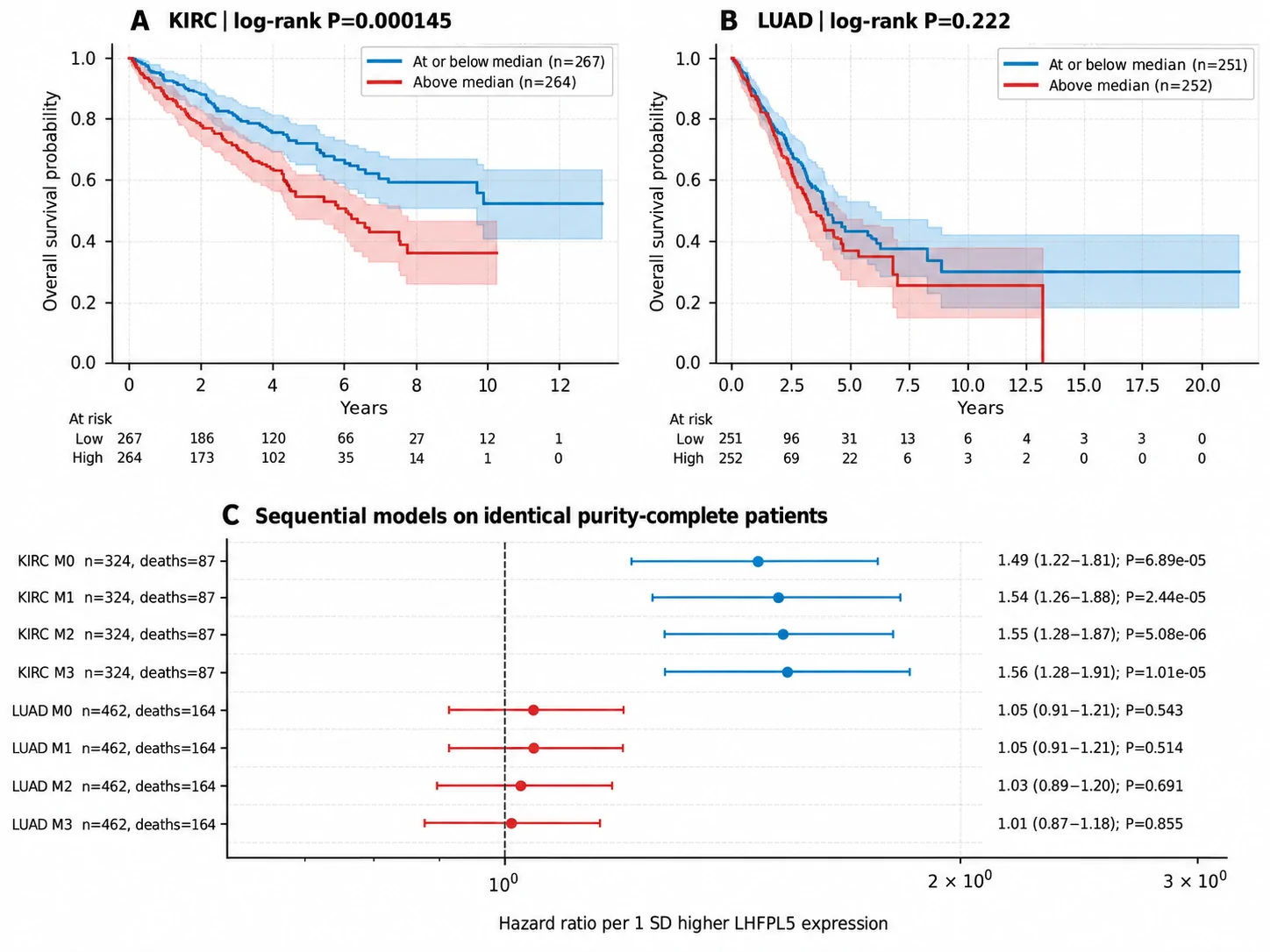
Overall survival according to *LHFPL5* expression and sequential continuous-expression Cox models. (**A**) Kaplan–Meier analysis in KIRC included 531 patients and 175 deaths. The low-expression group comprised 267 patients with 70 deaths, whereas the high-expression group included 264 patients with 105 deaths. (**B**) In LUAD, 503 patients and 182 deaths were analyzed; the low-expression group included 251 patients with 92 deaths and the high-expression group included 252 patients with 90 deaths. Median expression cutoffs were 0.368153711 for KIRC and 0.986265271 for LUAD, expressed as log2(TPM + 1) and calculated across all unique primary tumors. Values above the cohort-specific median were classified as high expression. Shaded areas represent 95% confidence intervals; numbers at risk and log-rank *p* values are displayed. (**C**) Sequential Cox models were fitted in identical purity-complete populations comprising 324 KIRC patients with 87 deaths and 462 LUAD patients with 164 deaths. M0 included *LHFPL5* expression alone; M1 additionally adjusted for age and sex; M2 further included pathological stage; and M3 added tumor purity. Points represent HRs per 1-SD increase in *LHFPL5* expression, with horizontal bars indicating 95% confidence intervals.

In contrast, no association between *LHFPL5* expression and overall survival was observed in LUAD using the primary STAR-derived expression dataset. The unadjusted HR in the clinical complete-case population was 1.05 (95% CI 0.91–1.20; *p* = 0.500), and remained null after adjustment for age, sex, and pathological stage (HR = 1.02, 95% CI 0.89–1.18; *p* = 0.770). Further adjustment for ABSOLUTE purity produced a similar estimate (HR = 1.01, 95% CI 0.87–1.18; *p* = 0.855; 462 patients, 164 deaths). The lack of association in LUAD was therefore apparent before covariate adjustment rather than emerging after controlling for measured confounders. Consistent with the continuous-expression models, median-split Kaplan–Meier analyses yielded log-rank *p* = 0.000145 for KIRC and *p* = 0.222 for LUAD (Figure 7; Table 2; Supplementary Table S2).

For the secondary Kaplan–Meier analyses, the KIRC cohort included 267 patients with 70 deaths at or below the median expression level and 264 patients with 105 deaths above the median. The corresponding LUAD groups comprised 251 patients with 92 deaths and 252 patients with 90 deaths, respectively. Median expression cutoffs were 0.368153711 for KIRC and 0.986265271 for LUAD, expressed as log2(TPM + 1) and calculated across all unique primary tumors. Binary high-versus-low Cox estimates are reported separately in Supplementary Table S2 and should be interpreted as group contrasts rather than as equivalents of the primary continuous per-SD effect estimates.

### 3.5. Clinicopathological Context and Model Robustness

STAR-derived *LHFPL5* expression showed no significant variation across pathological stage in KIRC (N = 530, *p* = 0.450; epsilon-squared = 0) or LUAD (N = 508, *p* = 0.335; epsilon-squared = 0.000773), and no association with tumor grade was observed in KIRC (N = 525, *p* = 0.315; epsilon-squared = 0.00104). In contrast, LHFPL5 expression differed across KIRC mRNA subtypes (N = 417, *p* = 3.54 × 10^−9^; *q* = 1.42 × 10^−8^; epsilon-squared = 0.0950). Median log2(TPM + 1) expression values for subtypes 1–4 were 0.368, 0.557, 0.424, and 0.240, respectively. Subtype-specific sample sizes and BH-adjusted pairwise comparisons are provided in the supplementary results.

The association between higher *LHFPL5* expression and poorer overall survival in KIRC remained evident after additional clinicopathological adjustment. Inclusion of tumor grade yielded an HR of 1.38 (95% CI 1.23–1.56; *p* = 1.21 × 10^−7^; 520 patients, 173 deaths). Among patients with available mRNA subtype information, additional subtype adjustment reduced the matched clinical HR from 1.36 to 1.25 (95% CI 1.06–1.46; *p* = 0.00691; 416 patients, 149 deaths), consistent with partial statistical overlap between subtype and the expression–survival association. In LUAD, adjustment for smoking exposure did not alter the null result (HR = 1.10, 95% CI 0.93–1.30; *p* = 0.288; 333 patients, 118 deaths).

Global proportional-hazards tests indicated departures from proportionality in the clinical models for both KIRC (*p* = 0.0146) and LUAD (*p* = 0.0424), largely attributable to categorical covariates. After stratification by stage and sex, the LHFPL5 estimate remained significant in KIRC (HR = 1.31, 95% CI 1.16–1.47; *p* = 1.9 × 10^−5^) and remained null in LUAD (HR = 1.04, 95% CI 0.90–1.20; *p* = 0.557). The global proportional-hazards test was nonsignificant for the KIRC purity-adjusted model (*p* = 0.234) but remained significant for the corresponding LUAD model (*p* = 0.0321), leading to additional stratified and time-varying analyses. Some covariate-specific departures persisted. In an exploratory KIRC time-varying model, the HR for *LHFPL5* was 1.33 (95% CI 1.02–1.73) during the first three years and 2.07 (95% CI 1.39–3.10) thereafter; the interaction test was borderline (*p* = 0.0552). Allowing flagged purity and Treg effects in LUAD to vary over time did not materially change the null *LHFPL5* estimate.

Sensitivity analyses excluding the highest 1% of expression values produced similar findings, with the KIRC association persisting (HR = 1.44, 95% CI 1.24–1.67) and no association in LUAD (HR = 1.01, 95% CI 0.87–1.17). Evidence for nonlinearity in KIRC varied according to model specification: the quadratic term was significant (*p* = 0.0152), whereas the three-degree-of-freedom spline test was borderline (*p* = 0.0519). No evidence of nonlinearity was detected in LUAD (*p* = 0.181). Accordingly, the reported per-SD HRs should be interpreted as summary estimates under an approximately log-linear model. Independent implementations using Efron handling of tied events reproduced the primary coefficients to within 10^−7^.

Complete model coefficients, matched base models, expression-scaling constants, Schoenfeld and global proportional-hazards diagnostics, nonlinearity tests, and influence analyses are provided in Supplementary Tables S1, S2 and S11. Stage-related plots and additional diagnostic figures are shown in Figures S1 and S9–S11. Treatment-adjusted analyses and LUAD subtype-adjusted models were not conducted due to the unavailability of suitable covariate data.

### 3.6. Transcriptomic Programs Associated with LHFPL5

Covariate-adjusted transcriptome-wide models included 326 KIRC and 466 LUAD patients, independent of survival-data availability. In KIRC, 20,016 genes met the inclusion criteria, of which 10,574 were positively and 691 negatively associated with *LHFPL5* expression at BH-adjusted *q* < 0.05. In LUAD, 12,226 of 21,024 tested genes showed positive associations, and 854 showed negative associations. The marked predominance of positive gene-level associations indicates a broad correlated transcriptional background. Accordingly, downstream gene-set enrichment was interpreted in relative terms, with recognition that residual technical or compositional effects may remain.

**Figure 8:**
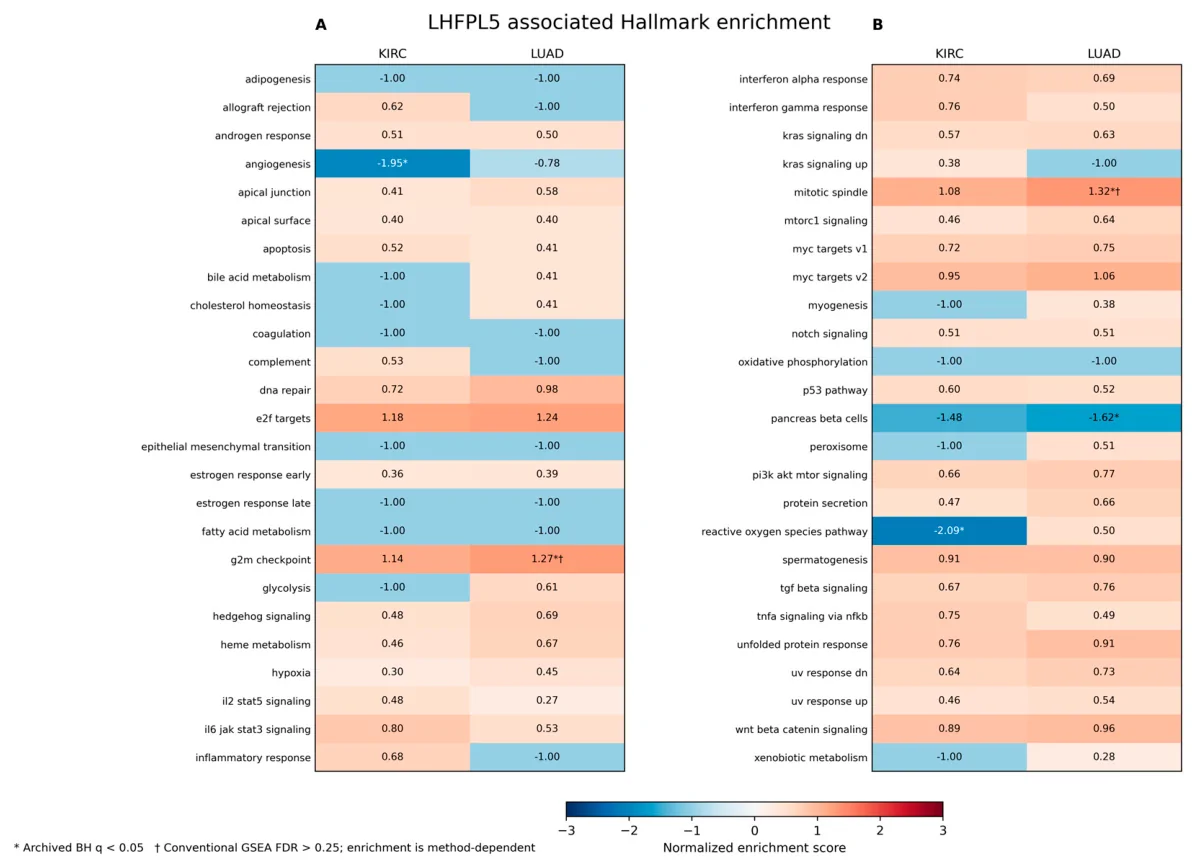
Hallmark enrichment associated with *LHFPL5* expression in KIRC and LUAD. (**A**,**B**) All 50 Hallmark sets were evaluated in each cancer using signed HC3 t-statistic rankings adjusted for age, sex, stage, and purity. Cell values show NES. Asterisks identify sets meeting the archived doubled-nominal-*p*/BH *q* < 0.05 convention; daggers identify LUAD G2M-checkpoint and mitotic-spindle sets that did not meet conventional GSEA FDR < 0.25 despite passing the archived BH criterion. Negative NES denotes relative rank enrichment and does not imply uniform downregulation of every pathway gene. Table S4 provides original nominal *p* values, reported two-sided *p* values, BH *q* values, conventional GSEA FDR, and leading-edge genes. “Pancreas beta cells” is a Hallmark gene-set label and does not denote inferred pancreatic tissue in LUAD.

In KIRC, the Hallmark Reactive Oxygen Species (ROS) and Angiogenesis gene sets met the archived doubled-nominal-*p*/BH significance criterion, with normalized enrichment scores (NES) of −2.093 and −1.950, respectively; both had BH *q* = 0.00500. In LUAD, the Pancreas Beta Cells, Mitotic Spindle, and G2M Checkpoint gene sets met the same criterion (NES = −1.623, 1.319, and 1.274; BH *q* = 0.0100, 0.0100, and 0.0167, respectively). However, the conventional GSEA FDR values for the LUAD Mitotic Spindle and G2M Checkpoint sets were 0.267 and 0.258, exceeding the commonly used exploratory threshold of 0.25. These two enrichment signals were therefore considered dependent on the statistical correction framework rather than consistently supported across approaches. The conventional GSEA FDR for KIRC Angiogenesis was 0.0366. Software-reported FDR values of zero for the KIRC ROS and LUAD Pancreas Beta Cells sets reflect the finite resolution of the permutation procedure and should not be interpreted as probabilities of exactly zero (Figure 8; Table 3; Supplementary Table S4).

Sample-level pathway scores provided a complementary view of these relationships. In KIRC, the ROS score was inversely correlated with *LHFPL5* expression (Spearman ρ = −0.343; *p* = 1.95 × 10^−10^). By contrast, the Angiogenesis score showed a modest positive correlation (ρ = 0.130; *p* = 0.0193), despite the negative NES observed in the preranked enrichment analysis. Consistent with this discrepancy, 27 leading-edge genes had positive gene-level coefficients compared with seven showing negative coefficients. The data therefore do not support a simple interpretation of reduced angiogenic activity with increasing *LHFPL5* expression. In LUAD, G2M Checkpoint and Mitotic Spindle scores were positively correlated with *LHFPL5* (ρ = 0.156 and 0.240, respectively), whereas the Pancreas Beta Cells score was inversely correlated (ρ = −0.127). The latter is a Hallmark transcriptional signature and does not indicate the presence of pancreatic cells in LUAD samples.

Complete gene-level results and results for all 50 Hallmark gene sets in each cancer, including nonsignificant findings, are provided in Supplementary Tables S3 and S4. Transcriptome-wide volcano plots and selected running-enrichment plots are shown in Figures S2–S5. Unadjusted transcriptome-wide correlations are reported separately from the covariate-adjusted analyses. These associations define transcriptional patterns accompanying *LHFPL5* expression but do not establish direct regulation of the corresponding genes or biological pathways by *LHFPL5*.

### 3.7. Pathway and Immune Context of LHFPL5

In KIRC, neither of the selected pathway scores was independently associated with overall survival after adjustment for clinical covariates and tumor purity, and inclusion of these scores did not materially reduce the *LHFPL5* survival coefficient (Table 3; Supplementary Table S5). In LUAD, by contrast, higher G2M Checkpoint and Mitotic Spindle scores were associated with poorer overall survival in the combined models. The corresponding estimates were HR = 1.35 (95% CI 1.14–1.59; *p* = 0.000376; *q* = 0.00113) for G2M Checkpoint and HR = 1.26 (95% CI 1.07–1.49; *p* = 0.00605; *q* = 0.00908) for Mitotic Spindle. *LHFPL5* itself remained unrelated to survival in these models. The pathway estimates remained above 1 in stage- and sex-stratified analyses, including models that allowed the purity effect to vary over time. These proliferation-related programs therefore provide clinically relevant transcriptomic context in LUAD, but they do not account for an *LHFPL5*-related survival effect because no such effect was observed in the primary LUAD analysis.

**Figure 9:**
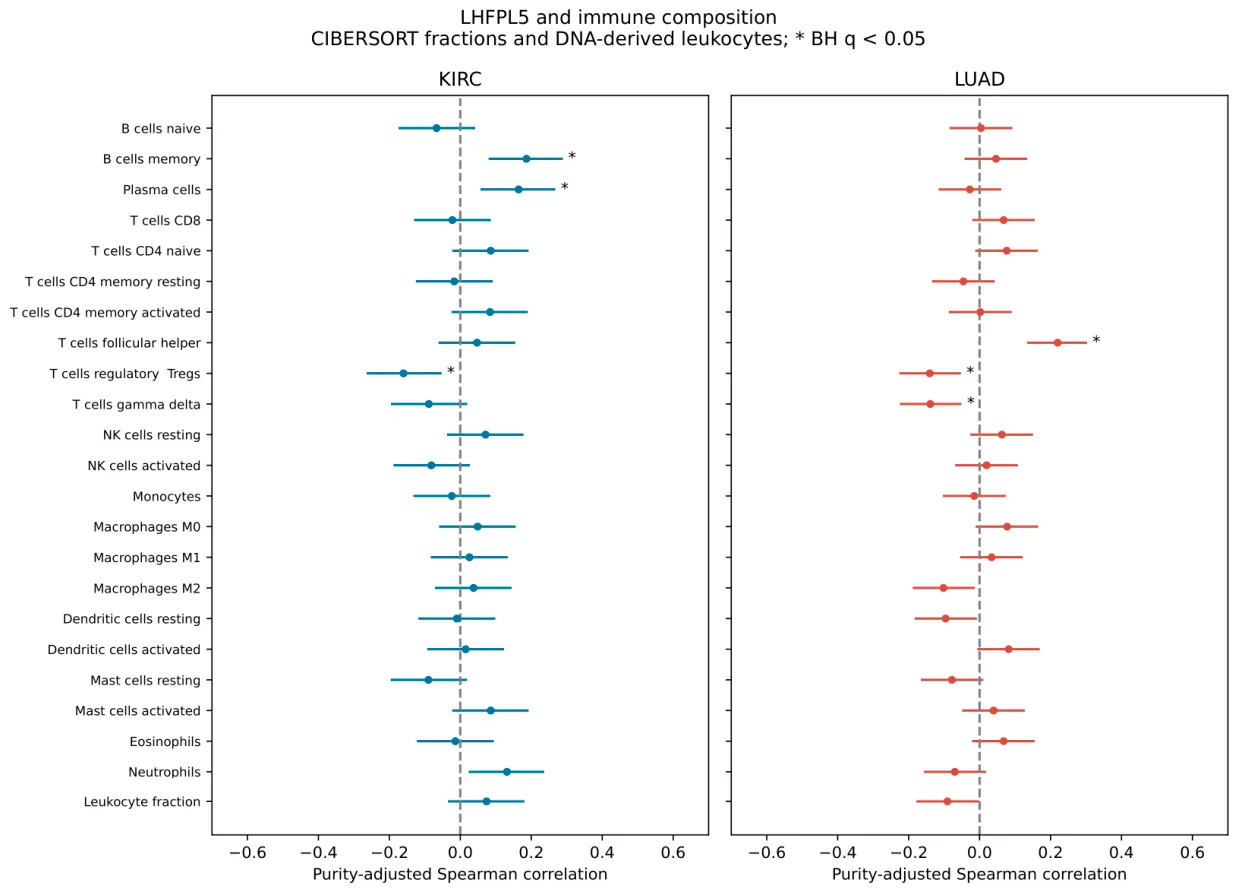
Purity-adjusted associations between *LHFPL5* expression and immune-cell composition. Partial Spearman correlations were estimated between *LHFPL5* expression and 22 published CIBERSORT relative immune-cell fractions, together with the DNA-derived leukocyte fraction, after residualizing ranked variables against ranked ABSOLUTE tumor purity. Bars indicate approximate Fisher *z*-based 95% confidence intervals, and asterisks denote associations meeting the Benjamini–Hochberg threshold of *q* < 0.05 across 23 tests within each cancer. Supplementary Table S6 provides model-specific sample sizes, nominal *p* values, adjusted *q* values, CIBERSORT quality-sensitivity analyses, complementary Bindea-signature results, and immune-feature survival models.

Tumor-purity analyses showed a weak inverse correlation between *LHFPL5* expression and ABSOLUTE purity in KIRC (Spearman ρ = −0.156; N = 328; *p* = 0.00463), whereas no corresponding relationship was observed in LUAD (ρ = 0.050; N = 490; *p* = 0.269). After adjustment for purity, KIRC *LHFPL5* expression was positively associated with memory B-cell (ρ = 0.187; *q* = 0.0161) and plasma-cell fractions (ρ = 0.164; *q* = 0.0284), and inversely associated with regulatory T-cell fractions (Tregs; ρ = −0.160; *q* = 0.0284). In LUAD, the strongest association was with follicular-helper T cells (ρ = 0.220; *q* = 2.03 × 10^−5^), with weaker inverse associations with Tregs and gamma-delta T cells (ρ = −0.140 and −0.139, respectively; both *q* = 0.0160). CD8 T-cell fractions did not remain significant after FDR correction in either cancer (Figure 9; Supplementary Table S6).

The extent to which these immune associations overlapped with the KIRC survival signal was examined in matched models comprising 324 patients and 87 deaths. Adding memory B-cell fraction reduced the *LHFPL5* HR from 1.56 to 1.44 (95% CI 1.16–1.79; *p* = 0.00103), whereas inclusion of plasma-cell fraction yielded an HR of 1.49 (95% CI 1.23–1.81; *p* = 5.25 × 10^−5^). The corresponding immune-feature estimates were HR = 1.18 for memory B cells (95% CI 1.01–1.37; *p* = 0.0344; *q* = 0.0344) and HR = 1.33 for plasma cells (95% CI 1.09–1.62; *p* = 0.00530; *q* = 0.0106). These modest changes in the *LHFPL5* coefficient are consistent with partial statistical overlap but do not establish mediation. In LUAD, the selected immune fractions were not independently associated with overall survival and did not alter the null *LHFPL5* estimate.

Agreement between immune-deconvolution approaches was limited. The inverse associations with Tregs and gamma-delta T cells in LUAD were observed with both CIBERSORT fractions and Bindea signatures, whereas the follicular-helper T-cell association did not remain FDR significant in the Bindea analysis. The KIRC Treg association was likewise not reproduced using Bindea scores. Because similarly named features from different deconvolution frameworks do not necessarily represent equivalent cell populations, and both approaches were applied to the same TCGA tumors, these comparisons were interpreted as cross-method consistency rather than independent biological replication. A validated stromal-score analysis was not available.

### 3.8. DNA Methylation and Copy-Number Associations

**Figure 10:**
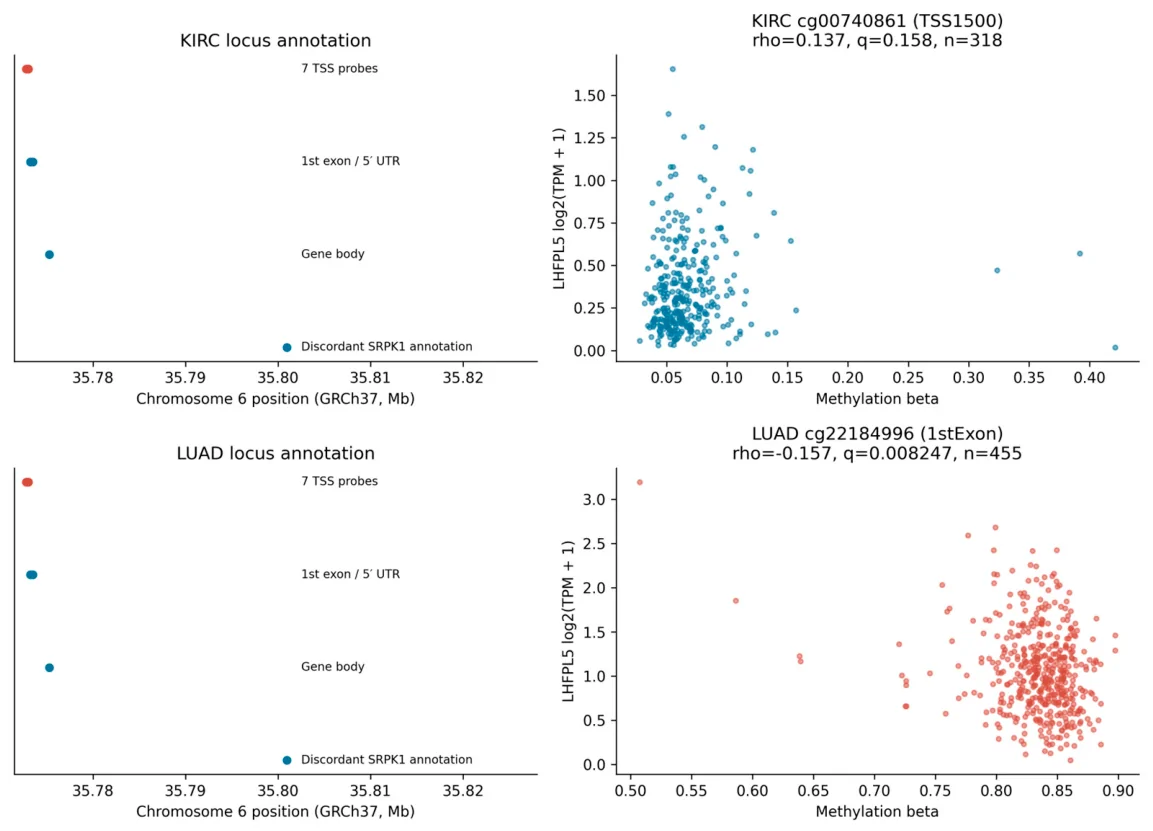
*LHFPL5* methylation-probe annotation and methylation–expression associations. Left panels show candidate probe locations on chromosome 6 (GRCh37), including the annotation-discordant *SRPK1* probe. Right panels show matched tumor associations for KIRC cg00740861, which was not FDR-significant, and LUAD cg22184996, which showed a weak inverse association with *LHFPL5* expression. The primary analysis included 11 candidate probes; full annotations, statistics, adjusted models, and the ten-probe LUAD sensitivity analysis are provided in Supplementary Table S7. The cg11529128 promoter association reached *q* < 0.05 only in the restricted sensitivity analysis, indicating testing-family dependence.

Matched DNA methylation data were available for 318 KIRC and 455 LUAD patients. None of the candidate probes in KIRC remained significant after Benjamini–Hochberg correction. In LUAD, the manifest-confirmed first-exon probe cg22184996 showed a weak inverse association with *LHFPL5* expression (Spearman ρ = −0.157; *p* = 0.000750; *q* = 0.00825). The TSS200 probe cg11529128 was borderline under the primary 11-probe correction framework (*q* = 0.05009) and was therefore not classified as significant. More broadly, promoter-region probes did not show a stronger or consistently inverse relationship with *LHFPL5* expression than non-promoter probes (Figure 10; Supplementary Table S7).

In the annotation-sensitivity analysis restricted to the ten manifest-confirmed *LHFPL5* probes, cg22184996 remained significant in LUAD (*q* = 0.00750). Under this reduced testing family, the weaker inverse association observed for the TSS200 probe cg11529128 also crossed the FDR threshold (ρ = −0.122; *p* = 0.00911; *q* = 0.0455). When the annotation-discordant *SRPK1* candidate was retained in the original 11-test family, the corresponding *q* value for cg11529128 was 0.05009. Because this probe’s classification depended on the definition of the multiple-testing family, the finding was considered threshold-sensitive and does not support a robust promoter-wide methylation pattern. The probe identifier cg00740861 was used throughout the revised analysis, correcting the earlier typographical error cg000740861. The discordant cg18451957 probe was retained for audit purposes but excluded from biological interpretation of *LHFPL5*.

The inverse association between cg22184996 methylation and *LHFPL5* expression remained evident after simultaneous adjustment for clinical covariates, tumor purity, and copy number in LUAD (N = 412; β = −0.503 expression SD per 0.1-unit increase in methylation beta; 95% CI −0.821 to −0.185; *q* = 0.00432). In contrast, several TSS probes showed positive conditional coefficients, and the exploratory seven-probe promoter mean was also positively associated with *LHFPL5* expression. This heterogeneous pattern is not consistent with a simple model in which uniform promoter hypomethylation drives higher expression. In the combined LUAD Cox model, cg22184996 was not associated with overall survival (HR per SD = 1.17, 95% CI 0.96–1.43; *p* = 0.114), and the *LHFPL5* survival estimate remained null.

Copy-number analyses showed a weak positive correlation between continuous GISTIC copy ratio and *LHFPL5* expression in KIRC (ρ = 0.095; N = 507; *p* = 0.0318; *q* = 0.0424), whereas expression did not differ significantly across categorical copy-number states (*p* = 0.139). The association was more pronounced in LUAD, where copy ratio correlated with *LHFPL5* expression (ρ = 0.289; N = 508; *p* = 3.01 × 10^−11^; *q* = 1.21 × 10^−10^), and the categorical comparison was also significant (*p* = 3.40 × 10^−10^). These locus-level associations are consistent with a possible genomic-dosage contribution to *LHFPL5* expression, but they do not establish direct causal regulation (Supplementary Table S8).

### 3.9. Integrated Molecular Correlates of LHFPL5 Expression

Integrated expression models included 238 KIRC and 412 LUAD patients (Table 4). In KIRC, the model explained only a small proportion of the variation in standardized *LHFPL5* expression (R^2^ = 0.0450; adjusted R^2^ = 0.0116), and the promoter mean, copy ratio, and tumor purity were not individually associated with expression at *p* < 0.05. The LUAD model showed greater explanatory capacity (R^2^ = 0.146; adjusted R^2^ = 0.129). Copy ratio was positively associated with *LHFPL5* expression (β = 1.080 SD per log2 copy-ratio unit; 95% CI 0.710–1.450; *p* = 1.03 × 10^−8^), as was the exploratory promoter mean (β = 0.190 SD per 0.1-unit increase in methylation beta; 95% CI 0.0677–0.313; *p* = 0.00237). Within the same LUAD patients, inclusion of copy ratio increased R^2^ by 0.112. This incremental R^2^ is descriptive of conditional model fit and should not be interpreted as a causal partitioning of expression variance.

Variance-inflation factors were <1.24, indicating limited multicollinearity among model covariates. In LUAD, the Breusch–Pagan test indicated heteroskedasticity (*p* = 0.0149), supporting HC3-robust confidence intervals. The KIRC model contained an influential observation (maximum Cook’s distance = 0.331), which further limits interpretation of the comparatively weak integrated associations in this cohort. A fully adjusted KIRC Cox model incorporating the extended methylation variables was not fitted because the overlapping dataset included 236 patients and 67 deaths, below the prespecified 80-event threshold for an eight-coefficient model. Complete model coefficients, incremental comparisons, and residual diagnostics are provided in Supplementary Table S8 and Figure S12.

### 3.10. External Reproducibility and RNA-Processing Sensitivity

**Figure 11:**
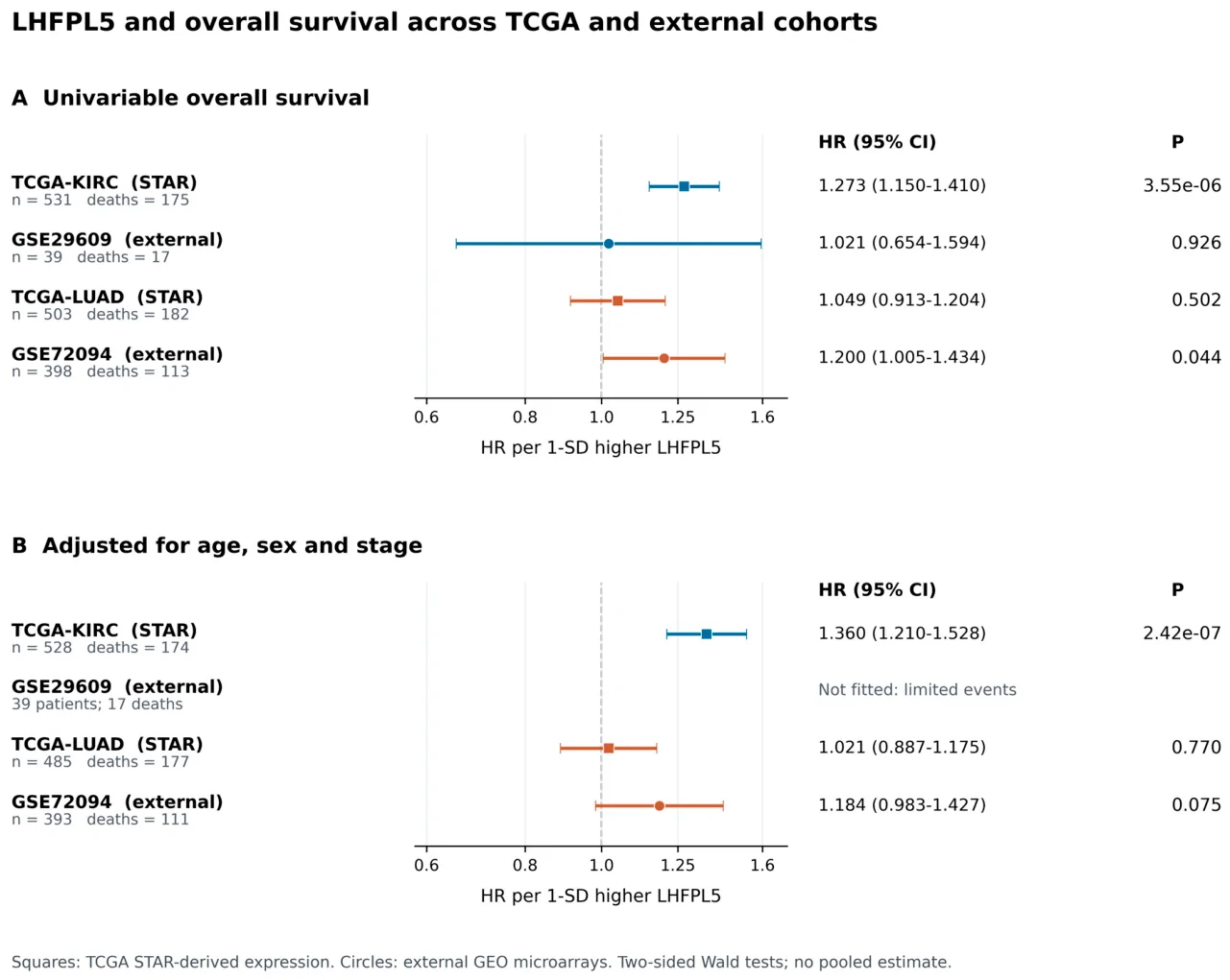
External cohort and TCGA overall-survival estimates for *LHFPL5*. (**A**) Univariate models and (**B**) age-, sex-, and stage-adjusted models. TCGA estimates use STAR-derived expression. Points represent HRs per 1-SD higher *LHFPL5* expression and bars show 95% CIs; the dashed reference line denotes HR = 1. KIRC and LUAD are shown separately, with squares for TCGA and circles for external GEO cohorts. Rows report fitted sample size, deaths, and two-sided Wald *p* values. GSE29609 adjustment was not fitted because of event and covariate limitations. Expression was standardized within each cohort, and results were not pooled. The adjusted GSE72094 estimate is inconclusive. Complete proportional-hazards diagnostics and matched complete-case comparisons are provided in Table 5 and Supplementary Table S10.

The external KIRC cohort GSE29609 included 39 patients and 17 deaths. The continuous-expression survival estimate was close to null and imprecise (HR = 1.021, 95% CI 0.654–1.594; *p* = 0.9258), and the limited number of events precluded a clinically adjusted model. In GSE72094, 398 of 442 measured LUAD samples had evaluable overall-survival data, including 113 deaths. The univariable estimate was HR = 1.200 (95% CI 1.005–1.434; *p* = 0.0442). Restriction to 393 complete cases with 111 deaths yielded an unadjusted HR of 1.190 (95% CI 0.995–1.424; *p* = 0.0571), while adjustment for age, sex, and stage gave HR = 1.184 (95% CI 0.983–1.427; *p* = 0.0754). These results indicate that both complete-case selection and covariate adjustment affected the external LUAD estimate (Figure 11; Table 5; Supplementary Table S10).

In the adjusted GSE72094 model, stage II showed evidence of nonproportional hazards (*p* = 0.0195), although the global proportional-hazards test was not significant (*p* = 0.1026). A stage-stratified model produced a similar estimate (HR = 1.189, 95% CI 0.986–1.435; *p* = 0.0703). Overall, the available external datasets did not provide convincing replication of the adjusted prognostic results. GSE68465 was excluded before outcome modeling because none of the 22,283 GPL96 probe records contained an annotated match for *LHFPL5*.

Expression measurements also showed only moderate agreement between STAR-derived and legacy RSEM TCGA data. In KIRC, Spearman ρ was 0.349, and Pearson *r* was 0.490 across 533 matched patients; in LUAD, the corresponding values were 0.389 and 0.464 across 515 patients. Agreement in median-based expression classification was 61.7% for KIRC and 66.2% for LUAD. Within identical clinical complete-case populations, KIRC retained an adverse survival association under both STAR (HR = 1.360, 95% CI 1.210–1.528; *p* = 2.42 × 10^−7^) and RSEM processing (HR = 1.216, 95% CI 1.068–1.383; *p* = 0.0031; 528 patients, 174 deaths). In LUAD, however, the estimate depended on the processing source: STAR-derived expression remained null (HR = 1.020, 95% CI 0.886–1.173; *p* = 0.7861), whereas the RSEM-based estimate was modestly elevated (HR = 1.159, 95% CI 1.003–1.339; *p* = 0.0453; 484 patients, 177 deaths). The matched LUAD population differed from the primary clinical model by one patient and is therefore reported separately (Supplementary Table S11; Figures S7 and S8).

Global proportional-hazards departures were observed in the adjusted processing-comparison models (*p* = 0.0052–0.0447). When stratified jointly by stage and sex, the adverse trend for KIRC remained consistent across both processing sources (STAR HR = 1.305; RSEM HR = 1.166), and the contrast between STAR (HR = 1.042) and RSEM (HR = 1.177) for LUAD was preserved. Stratified rank diagnostics did not indicate a violation of the proportional-hazards assumption for *LHFPL5* itself (all *p* > 0.32); however, this does not confirm strict proportionality. Expression levels in legacy KIRC also varied according to stage, whereas the STAR-derived measure did not show such variation. Since the comparison encompasses differences in quantification, annotation, processing, and potentially specimen selection, it cannot be interpreted as an effect attributable solely to the aligner. Similarly, a significant finding in one pipeline accompanied by a nonsignificant result in another does not, in itself, imply that the underlying effect estimates differ statistically.

### 3.11. Single-Cell Localization of LHFPL5

In the original ccRCC analysis, GSE159115 comprised 20,748 cells from seven patients, of which 34 were *LHFPL5*-positive. These included 31 of 9139 author-annotated tumor cells, two mural cells, and one myeloid cell. In GSE131907, *LHFPL5* was detected in 25 of 45,149 primary LUAD cells from 11 patients, including 17 of 7270 tumor-associated epithelial cells, five myeloid cells, and one cell each from the endothelial, mast-cell, and T/NK compartments. Median expression was zero across all annotated populations. All planned KIRC comparisons and most LUAD comparisons failed the prespecified requirements for detection frequency or nonzero paired differences. The only LUAD comparison meeting these criteria, epithelial versus myeloid cells, was not statistically significant (*p* = 0.09375; 11 patients; Supplementary Table S9; Figure S6).

**Figure 12:**
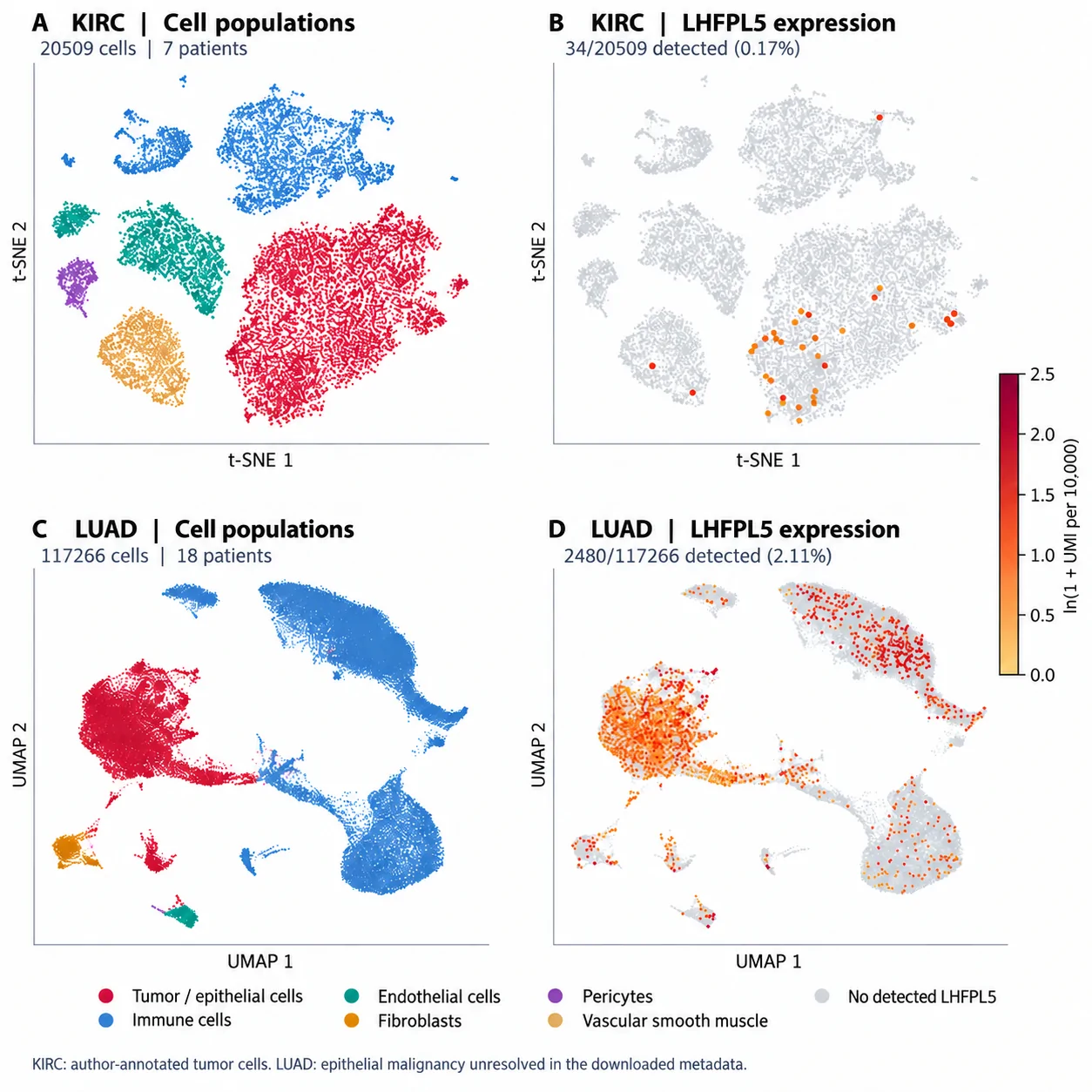
Single-cell localization of *LHFPL5* in non-TCGA ccRCC and LUAD datasets. (**A**,**B**) t-SNE representation of the curated GSE159115 ccRCC dataset, comprising 20,509 cells from seven patients, showing broad cell lineages and *LHFPL5* expression. (**C**,**D**) UMAP representation of the KAP241022 LUAD dataset, comprising 117,266 cells from 18 patients. All exported cells are displayed. Gray points indicate zero *LHFPL5* UMI counts, whereas colored points represent ln(1 + UMI per 10,000) on the displayed 0–2.5 scale. *LHFPL5*-positive kidney cells were plotted above other cells and enlarged to improve visibility. Kidney tumor labels were retained from the source annotation, whereas malignant status could not be resolved for LUAD epithelial cells in the analyzed export. Embeddings and coordinate axes are dataset-specific and should not be compared directly. These plots describe the cellular distribution of *LHFPL5* and do not constitute validation of bulk clinical associations. Results from GSE131907 are presented in Figure S6 and Supplementary Table S9.

**Figure 13:**
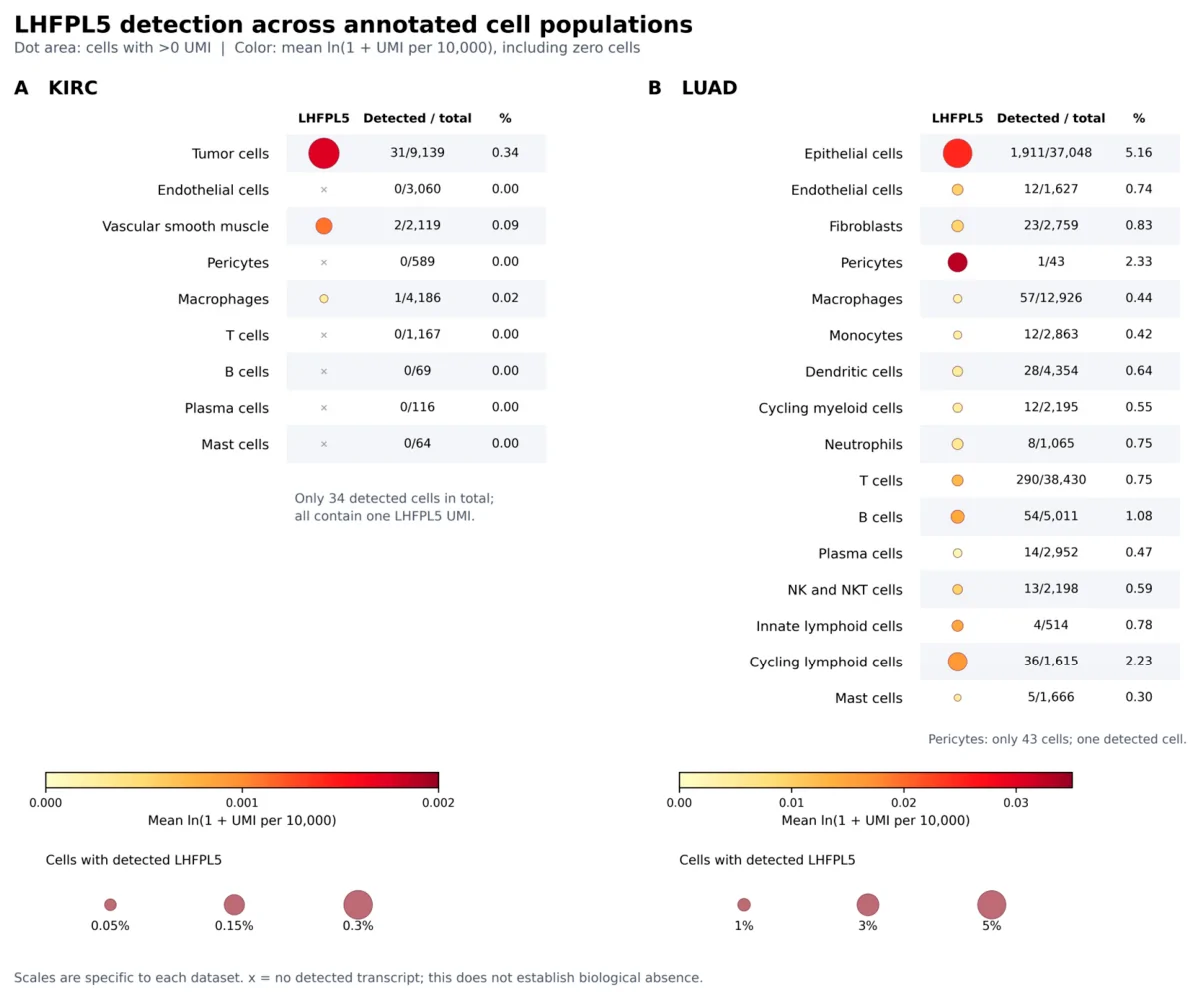
*LHFPL5* detection across annotated single-cell populations. (**A**) Curated GSE159115 ccRCC and (**B**) KAP241022 LUAD. Dot area represents the proportion of cells with >0 raw *LHFPL5* UMI; color represents the mean ln(1 + UMI per 10,000) calculated across all cells, including zeros. Numeric columns report positive/total cells and percentages. Size and color scales are specific to each dataset. A cross denotes zero detected transcripts and should not be interpreted as biological absence. The LUAD pericyte estimate is based on one positive cell among 43 cells. All cell-type medians are zero. Every positive kidney cell contained one UMI; at least two UMIs remained detectable in 245 LUAD epithelial cells. Donor-level summaries and threshold sensitivities are provided in Table S9.

The curated GSE159115 export contained 20,509 cells from the same seven ccRCC patients. The number of *LHFPL5*-positive cells was unchanged, comprising 31 tumor cells, two vascular smooth-muscle cells, and one macrophage. Positive cells were confined to three patients, and each contained a single *LHFPL5* UMI. No cells remained positive when the detection threshold was increased to two UMIs. The 20,748-cell and 20,509-cell GSE159115 datasets therefore represent alternative exports of the same patient cohort and were not considered independent replication (Figure 12 and Figure 13; Supplementary Table S9).

The KAP241022 LUAD cohort showed a distinct expression pattern and included 117,266 cells from 18 patients. *LHFPL5* was detected in 2480 cells, with epithelial cells accounting for the largest fraction of positive cells (1911/37,048; 5.16%) and 77.1% of all detections. Epithelial expression was observed in all 18 patients, with a median donor-level detection rate of 4.74% (range, 0.62–23.69%). Detection frequencies were lower in T cells (0.75%), macrophages (0.44%), endothelial cells (0.74%), and fibroblasts (0.83%). The apparent 2.33% detection rate in pericytes reflected a single positive cell among only 43 cells and should therefore be interpreted cautiously. At the more stringent threshold of at least two UMIs, 245 epithelial cells remained positive (0.66%). Median expression remained zero in every annotated cell type (Figure 12 and Figure 13).

Mean normalized expression, expressed as ln(1 + UMI per 10,000), was 0.02486 in KAP241022 epithelial cells and 0.001741 in the curated ccRCC tumor compartment. Direct quantitative comparison between datasets is limited by differences in sequencing chemistry, depth, filtering procedures, and export structure. In addition, the epithelial annotations in KAP241022 did not distinguish malignant from nonmalignant epithelial cells. The marked difference between the sparse detection observed in GSE131907 and the broader epithelial distribution in KAP241022 indicates that *LHFPL5* detection is strongly dataset-dependent. Accordingly, these single-cell analyses define the cellular distribution of *LHFPL5* in independent non-TCGA datasets but do not validate the TCGA survival or molecular associations.

## 4. Discussion

The present study identifies distinct molecular and clinical contexts for *LHFPL5* in clear cell renal cell carcinoma (KIRC) and lung adenocarcinoma (LUAD). Although *LHFPL5* expression was increased in both cancers, its prognostic implications differed. In KIRC, higher expression remained associated with poorer overall survival after clinical adjustment and across several sensitivity analyses, whereas the primary STAR-derived LUAD analysis showed no comparable survival association despite clearer relationships with copy number, DNA methylation, and proliferation-related transcriptional programs. These findings indicate that molecular correlates of *LHFPL5* expression do not necessarily translate into prognostic relevance and support a cancer-specific rather than universal interpretation of its role.

This context dependence is particularly important because *LHFPL5* is best known for its role in cochlear mechanotransduction [1,2]. Although its membrane-associated properties provide a rationale for investigating functions outside the inner ear, they do not establish a role in malignancy. Studies implicating other LHFPL family members, including LHFPL2 in renal carcinoma and LHFPL6 in gastric cancer, provide only indirect precedent [3,4]. Accordingly, the present findings should not be interpreted as evidence that LHFPL5 shares the proposed tumor-promoting or immune-related functions.

The tumor–normal expression differences also require cautious interpretation. Proteogenomic studies have documented extensive cancer-specific transcriptional remodeling in ccRCC and LUAD [26,27]. Thus, increased *LHFPL5* RNA may reflect altered tumor-cell identity or a broader tumor-associated state rather than functional dependence. This is particularly relevant because the approximately sevenfold increase in KIRC and 55-fold increase in LUAD occurred against very low normal expression, with tumor medians of only 0.020 and 0.120 TPM, respectively. Persistence in matched tumor–adjacent-normal comparisons strengthens evidence for a genuine tumor-associated RNA difference, whereas TCGA–GTEx comparisons remain susceptible to tissue-source and inter-study effects [28]. Concordance across TCGA-derived platforms therefore provides analytical support but should not be considered independent validation.

The KIRC survival association was comparatively robust. Higher *LHFPL5* expression was associated with poorer overall survival after adjustment for age, sex, and pathological stage (HR per SD = 1.36, 95% CI 1.21–1.53), with persistence after grade adjustment and in purity-adjusted and stratified analyses. Partial attenuation after mRNA-subtype adjustment suggests that *LHFPL5* captures information shared with broader molecular states while retaining a residual association. Nevertheless, persistence after covariate adjustment does not establish causality or clinical utility. Compared with multimodal biomarker studies integrating protein abundance, methylation, genomic instability, and histopathology [29], the current evidence remains limited by the absence of protein-level confirmation and convincing external replication.

In LUAD, by contrast, the adjusted HR of 1.02 (95% CI 0.89–1.18) provided no evidence of an independent adverse association in the primary STAR-derived dataset. The corresponding unadjusted estimate was also null, indicating that clinical adjustment alone does not explain the result. This is consistent with the concept that prognostic information in LUAD may reside in coordinated cellular or transcriptional states rather than in individual transcripts [30]. In our analysis, proliferation-related programs carried prognostic information, whereas *LHFPL5* itself did not reliably capture that signal.

External analyses reinforced the need for caution. GSE29609 included only 39 KIRC patients and 17 deaths, yielding an imprecise near-null estimate with a confidence interval compatible with the TCGA effect. In LUAD, the weak adverse association in GSE72094 became inconclusive after complete-case restriction and clinical adjustment. Differences in stage, genomic background, follow-up, treatment, platform characteristics, and probe specificity may contribute to these discrepancies [31], although these possibilities remain untested. The absence of a suitable *LHFPL5* probe in GSE68465 further illustrates that cohort size alone does not ensure suitability for validating a low-abundance transcript.

Processing sensitivity added another source of uncertainty. KIRC retained an adverse association under both STAR-derived and legacy RSEM expression estimates, whereas LUAD shifted from a null to a modest adverse association. Such discrepancies are biologically relevant because RNA-sequencing pipelines can generate substantial gene-specific variation [32]. In our comparison, differences may also reflect annotation, quantification procedures, and potentially different samples from the same patient. Therefore, KIRC demonstrates greater internal consistency, whereas LUAD requires more stable measurements before prognostic interpretation.

The regulatory analyses further emphasized this cancer-specific pattern. In KIRC, no tested methylation probe remained significant after multiple-testing correction. In LUAD, however, methylation at the first-exon probe cg22184996 was inversely associated with *LHFPL5* expression after adjustment for copy number, purity, and clinical variables, consistent with evidence that first-exon methylation can influence transcriptional silencing [33]. Nevertheless, the overall pattern did not support uniform promoter hypomethylation, because several TSS-associated probes showed positive conditional relationships and the exploratory promoter mean was also positively associated with expression. These findings therefore favor locus-specific epigenetic regulation rather than a simple promoter-wide mechanism.

Copy-number variation appeared more informative in LUAD. The positive copy-ratio association and incremental R^2^ of 0.112 suggest that genomic dosage contributes meaningfully to intertumoral variation in *LHFPL5* expression, consistent with broader proteogenomic observations in LUAD [27]. However, most expression variability remained unexplained, and the analysis cannot distinguish a selective *LHFPL5* event from expression changes accompanying a larger chromosomal alteration. Thus, genomic dosage represents a plausible regulatory contributor rather than evidence of an *LHFPL5*-specific oncogenic driver.

Transcriptomic analyses provided complementary insight into the biological states associated with *LHFPL5*. In KIRC, the inverse relationship with the reactive oxygen species signature was among the more consistent findings. Redox regulation is biologically relevant to ccRCC, including HIF-2α-associated dependencies on GPX4 and ferroptosis sensitivity [34]. However, a lower Hallmark ROS signature cannot be equated with reduced intracellular ROS or ferroptosis resistance. Moreover, the ROS score neither independently predicted survival nor materially attenuated the *LHFPL5* coefficient. The evidence therefore supports a redox-associated transcriptional context rather than a demonstrated mechanism linking *LHFPL5* to adverse outcome.

Angiogenesis also requires cautious interpretation. Although angiogenic programs are central to ccRCC biology [35], negative enrichment coexisted with a weak positive sample-level correlation and predominantly positive leading-edge coefficients. This pattern may reflect relative ranking effects within a globally positive transcriptional background rather than true suppression of angiogenesis. Furthermore, adjustment for the angiogenesis score did not materially reduce the survival association with *LHFPL5*. Angiogenesis is therefore relevant to the disease context but is not the established pathway mediating the observed prognostic relationship.

In LUAD, *LHFPL5* was more closely associated with G2M-checkpoint and mitotic-spindle programs, placing it within a proliferation-related transcriptional state. Such programs are biologically plausible in LUAD and may vary according to genomic background [31]. Nevertheless, enrichment was sensitive to the statistical convention used, and these pathways cannot mediate an *LHFPL5* survival effect because the primary gene-level survival association was absent. Similarly, the negatively associated Pancreas Beta Cells Hallmark set should be interpreted strictly as a transcriptional signature rather than evidence of pancreatic differentiation or cellular infiltration.

Tumor composition may further influence these bulk-transcriptomic relationships. In KIRC, weak inverse associations with purity and residual immune correlations suggest that *LHFPL5* expression may reflect both tumor-cell transcription and microenvironmental variation. Because CIBERSORT estimates relative immune fractions, changes in one inferred population may influence the apparent abundance of others. Deconvolution performance also depends on the reference and preprocessing strategy [36], and incomplete agreement with Bindea signatures therefore represents genuine analytical uncertainty.

The KIRC B-cell and plasma-cell associations illustrate this limitation. Although memory B-cell and plasma-cell fractions correlated positively with *LHFPL5* and adverse outcome, RCC immune biology is complex. B-cell maturation within tertiary lymphoid structures may contribute to antitumor immunity [37,38], indicating that abundance alone cannot define biological function. Accordingly, these correlations should not be interpreted as evidence that B cells are intrinsically tumor-promoting, are the principal source of *LHFPL5*, or are recruited directly by LHFPL5. In LUAD, the strongest CIBERSORT association involved follicular-helper T cells, but this relationship was not significant under the complementary Bindea framework. Together with the absence of robust CD8 T-cell associations, the findings provide no evidence that *LHFPL5* directly regulates immune exhaustion or checkpoint response.

The KAP241022 LUAD dataset provided stronger descriptive evidence of epithelial localization, with *LHFPL5* detections across all 18 donors and persistence in a subset of epithelial cells at a stricter threshold. However, per-cell malignancy labels were unavailable, so epithelial enrichment could not be equated with malignant-cell expression. Differences in histology, sequencing depth, chemistry, sampling, and filtering may also explain the contrast with sparse detection in GSE131907 [21,30,39]. Thus, the single-cell analyses add orthogonal cellular context but do not independently validate the bulk expression, survival, methylation, or immune findings.

Single-cell analyses offered useful but limited information regarding cellular origin. In the renal datasets, most *LHFPL5*-positive cells were classified as tumor cells, but all positive events contained only one UMI, were restricted to three donors, and disappeared under the two-UMI threshold. These findings therefore provide only tentative evidence of tumor-cell localization. Ambient RNA is a plausible explanation for isolated low-count detections [40], although genuine low-level transcription cannot be excluded because of limited single-cell capture efficiency.

Taken together, the data suggest that *LHFPL5* marks different biological states in KIRC and LUAD rather than executing a single conserved cancer function. In KIRC, its expression is associated with poorer survival and partly overlaps with broader molecular and immune states, while the regulatory determinants examined here explain relatively little of its expression. In LUAD, copy number and regional methylation show clearer relationships with expression, and proliferation programs carry prognostic information even though *LHFPL5* itself does not show a stable survival association. This contrast is consistent with the distinct genomic, metabolic, epigenetic, and microenvironmental architectures of renal and lung adenocarcinomas [26,27,29,31].

Several strengths support the present analysis, including integration of genomic, epigenetic, transcriptomic, clinical, immune, and single-cell data; continuous multivariable survival modeling; and explicit evaluation of external reproducibility and processing sensitivity. Nevertheless, the retrospective design, post-screening focus on KIRC and LUAD, incomplete clinical variables, differing complete-case populations, limited external events, measurement sensitivity, sparse single-cell expression, and absence of direct functional experiments limit causal interpretation. Most importantly, RNA-level associations do not establish corresponding protein abundance or biological activity.

Future studies should therefore establish measurement reliability and cellular origin using transcript-specific, spatial, and protein-level assays. Independent cohorts should evaluate a prespecified continuous-expression model and determine whether *LHFPL5* adds prognostic information beyond established clinical variables. Functional perturbation and rescue experiments in renal and lung models are also required to test effects on proliferation, oxidative-stress responses, and membrane-associated phenotypes. In LUAD, copy-number-aware experiments and locus-specific methylation editing may help distinguish direct regulatory effects from broader tumor-state associations. Overall, the current evidence supports a persistent within-TCGA adverse survival association in KIRC and a distinct, processing-sensitive molecular context in LUAD, positioning *LHFPL5* as a candidate for further investigation rather than an established prognostic biomarker or therapeutic target.

## 5. Conclusions

This integrative multi-omics study demonstrates that *LHFPL5* has distinct biological and clinical contexts in KIRC and LUAD rather than a uniform cancer-associated role. In KIRC, higher *LHFPL5* expression showed a persistent adverse association with overall survival across several adjusted and sensitivity analyses, suggesting that it may mark a clinically relevant tumor state. In contrast, LUAD showed stronger relationships between LHFPL5 expression and copy-number variation, locus-specific DNA methylation, and proliferation-related transcriptional programs, but no stable prognostic association in the primary analysis. Immune deconvolution and single-cell analyses further indicated that the cellular and microenvironmental context of *LHFPL5* is cancer-dependent and cannot be reduced to a single conserved mechanism.

Collectively, these findings position *LHFPL5* as a context-dependent molecular marker whose significance differs between renal and lung adenocarcinomas. The KIRC survival association warrants independent validation, whereas the LUAD findings highlight potentially relevant regulatory mechanisms that require functional confirmation. Further studies integrating spatial and protein-level validation, independent clinical cohorts, and direct perturbation experiments are needed to determine whether *LHFPL5* has biological, prognostic, or therapeutic relevance. At present, the data support *LHFPL5* as a promising candidate for mechanistic investigation rather than an established biomarker or therapeutic target.

## Figures and Tables

**Table 1:** Clinical characteristics of the nested KIRC and LUAD analysis populations.

Characteristic	KIRC Clinical	LUAD Clinical	KIRC Purity	LUAD Purity
Patients	528	485	324	462
Deaths	174	177	87	164
Age, years; median [IQR]	61.0 [52.0, 70.0]	66.0 [59.0, 72.0]	60.0 [51.0, 69.0]	66.0 [59.0, 72.0]
Male, n (%)	342 (64.8)	220 (45.4)	204 (63.0)	213 (46.1)
Stage I, n (%)	265 (50.2)	263 (54.2)	164 (50.6)	251 (54.3)
Stage II, n (%)	57 (10.8)	118 (24.3)	37 (11.4)	112 (24.2)
Stage III, n (%)	123 (23.3)	79 (16.3)	78 (24.1)	75 (16.2)
Stage IV, n (%)	83 (15.7)	25 (5.2)	45 (13.9)	24 (5.2)
Grade available, n	520	0	318	0
mRNA subtype available, n	416	0	244	0
ABSOLUTE purity available, n	324	462	324	462
Pack-years available, n	20	333	19	319

Clinical and purity-complete populations are nested complete-case subsets rather than independent cohorts. IQR, interquartile range. Grade, subtype, and pack-years were used only in prespecified sensitivity models; KIRC pack-years are shown for availability only. Full sample flow, missingness, and patient membership are reported in Table S1.

**Table 2:** Continuous *LHFPL5* expression and overall survival in TCGA-KIRC and TCGA-LUAD.

Population	Model	N	Deaths	HR per SD (95% CI)	*p*
KIRC clinical	M0	528	174	1.27 (1.15–1.41)	3.98 × 10^−6^
KIRC clinical	M1	528	174	1.33 (1.19–1.48)	2.60 × 10^−7^
KIRC clinical	M2	528	174	1.36 (1.21–1.53)	2.42 × 10^−7^
KIRC purity	M0	324	87	1.49 (1.22–1.81)	6.89 × 10^−5^
KIRC purity	M1	324	87	1.54 (1.26–1.88)	2.44 × 10^−5^
KIRC purity	M2	324	87	1.55 (1.28–1.87)	5.08 × 10^−6^
KIRC purity	M3	324	87	1.56 (1.28–1.91)	1.01 × 10^−5^
LUAD clinical	M0	485	177	1.05 (0.91–1.20)	0.500
LUAD clinical	M1	485	177	1.05 (0.92–1.21)	0.471
LUAD clinical	M2	485	177	1.02 (0.89–1.18)	0.770
LUAD purity	M0	462	164	1.05 (0.91–1.21)	0.543
LUAD purity	M1	462	164	1.05 (0.91–1.21)	0.514
LUAD purity	M2	462	164	1.03 (0.89–1.20)	0.691
LUAD purity	M3	462	164	1.01 (0.87–1.18)	0.855

M0, *LHFPL5* alone; M1, plus age per 10 years and sex; M2, plus pathological stages II–IV versus I; M3, plus ABSOLUTE purity. Each nested model family contains the same patients. *LHFPL5* expression was standardized using all unique primary tumors within each cancer.

**Table 3:** Selected Hallmark programs and combined overall-survival models.

Cancer and Pathway	rho	NES	BH *q*/GSEA FDR	Pathway HR (95% CI); *p*	LHFPL5 HR (95% CI); *p*
KIRC Angiogenesis	0.130	−1.950	0.005/0.037	1.09 (0.84–1.42); 0.499	1.58 (1.29–1.93); 8.33 × 10^−6^
KIRC Reactive Oxygen Species Pathway	−0.343	−2.093	0.005/0.000	1.04 (0.80–1.35); 0.774	1.59 (1.27–1.99); 5.32 × 10^−5^
LUAD G2M Checkpoint	0.156	1.274	0.017/0.258	1.35 (1.14–1.59); 3.76 × 10^−4^	0.95 (0.82–1.11); 0.542
LUAD Mitotic Spindle	0.240	1.319	0.010/0.267	1.26 (1.07–1.49); 0.006	0.95 (0.81–1.11); 0.493
LUAD Pancreas Beta Cells	−0.127	−1.623	0.010/0.000	0.87 (0.75–1.01); 0.067	1.00 (0.86–1.16); 0.972

Correlations include 326 KIRC or 466 LUAD patients. Combined Cox models include standardized *LHFPL5* expression, standardized pathway score, age, sex, stage, and purity (KIRC, N = 324 with 87 deaths; LUAD, N = 462 with 164 deaths). BH *q*-values follow the archived doubled-nominal-*p* convention; conventional GSEA FDR is shown separately. Software-reported zero FDR values reflect finite permutation resolution. LUAD G2M-checkpoint and mitotic-spindle enrichment is method-dependent. Full pathway selection, score-only models, survival-adjusted *q* values, and diagnostics are in Tables S4 and S5.

**Table 4:** Multivariable linear regression analysis of *LHFPL5* expression in relation to promoter DNA methylation, copy-number alterations, tumor purity, and clinicopathological characteristics in KIRC and LUAD.

Covariate	KIRC β (95% CI)	*p*	LUAD β (95% CI)	*p*
Promoter mean per 0.1 β	0.520 (−0.745–1.785)	0.421	0.190 (0.068–0.313)	0.002
Copy ratio per log2 unit	0.454 (−0.016–0.924)	0.058	1.080 (0.710–1.450)	1.03 × 10^−8^
ABSOLUTE purity per unit	−0.824 (−1.659–0.011)	0.053	0.050 (−0.519–0.618)	0.864
Age per 10 years	−0.043 (−0.131–0.045)	0.340	−0.043 (−0.137–0.052)	0.373
Male vs. female	−0.092 (−0.329–0.145)	0.445	0.062 (−0.130–0.253)	0.529
Stage II vs. I	0.098 (−0.269–0.464)	0.602	−0.065 (−0.293–0.163)	0.578
Stage III vs. I	0.060 (−0.193–0.313)	0.641	−0.174 (−0.417–0.069)	0.161
Stage IV vs. I	0.099 (−0.262–0.461)	0.591	0.294 (−0.172–0.759)	0.216

Outcome: standardized *LHFPL5* expression. KIRC, N = 238, R^2^ = 0.0450, adjusted R^2^ = 0.0116; LUAD, N = 412, R^2^ = 0.146, adjusted R^2^ = 0.129. Confidence intervals use HC3 robust standard errors. The promoter mean is an exploratory average of seven TSS probes and is not a validated regulatory score. Coefficients represent conditional associations. Intercepts, incremental R^2^, variance-inflation factors, and residual diagnostics are provided in Table S8.

**Table 5:** External cohort analyses of *LHFPL5* and overall survival.

Cohort	Model	N	Deaths	HR per SD (95% CI)	*p*	PH Gene/Global *p*
GSE29609	Univariable	39	17	1.021 (0.654–1.594)	0.926	0.875/0.882
GSE29609	Adjusted	39	17	Not fitted	NA	NA/NA
GSE72094	Univariable	398	113	1.200 (1.005–1.434)	0.044	0.086/0.081
GSE72094	Univariable CC	393	111	1.190 (0.995–1.424)	0.057	0.083/0.081
GSE72094	Adjusted	393	111	1.184 (0.983–1.427)	0.075	0.067/0.103
GSE72094	Stage stratified	393	111	1.189 (0.986–1.435)	0.070	0.079/0.287

Univariable CC denotes the same complete-case population used in the adjusted model. GSE72094 adjustment includes age, sex, and stage; the stage-stratified model adjusts for age and sex with stage-specific baseline hazards. The GSE29609 adjusted model was not fitted because event and covariate availability did not satisfy the retained modeling criterion; N and deaths on that row describe the available population only. PH gene denotes the scaled Schoenfeld rank test, and global denotes the joint Efron score test. GSE68465 was excluded before modeling. Full coefficients and participant lists are reported in Table S10.

## Data Availability

The datasets analyzed in this study are publicly available from The Cancer Genome Atlas (TCGA) through the UCSC Xena platform and from the NCBI Gene Expression Omnibus (GEO), including GSE29609, GSE72094, GSE159115, and GSE131907. Additional single-cell RNA-sequencing data were obtained from the Korean Nucleotide Archive under accession KAP241022. Analysis-ready data extracts, source records, model-specific patient lists, software and session information, and complete statistical outputs are provided in Supplementary Tables S1–S12 and the accompanying reproducibility files. This study involved no new patient recruitment or biospecimen collection.

## Supplementary Materials

The following supporting information can be downloaded at: https://www.mdpi.com/article/10.3390/medicina62101971/s1.

## Abbreviations

The following abbreviations are used in this manuscript:

ABSOLUTE	Algorithm for estimating tumor purity and ploidy
BH	Benjamini–Hochberg
ccRCC	Clear cell renal cell carcinoma
CI	Confidence interval
CNV	Copy-number variation
FDR	False discovery rate
GDC	Genomic Data Commons
GEO	Gene Expression Omnibus
GISTIC	Genomic Identification of Significant Targets in Cancer
GSEA	Gene set enrichment analysis
HC3	Heteroskedasticity-consistent covariance estimator, type 3
HR	Hazard ratio
IQR	Interquartile range
KIRC	Kidney renal clear cell carcinoma
LHFPL5	LHFPL tetraspan subfamily member 5
LUAD	Lung adenocarcinoma
MSigDB	Molecular Signatures Database
NES	Normalized enrichment score
OS	Overall survival
PH	Proportional hazards
RNA	Ribonucleic acid
RNA-seq	RNA sequencing
ROS	Reactive oxygen species
RSEM	RNA-Seq by Expectation Maximization
scRNA-seq	Single-cell RNA sequencing
SD	Standard deviation
ssGSEA	Single-sample gene set enrichment analysis
TCGA	The Cancer Genome Atlas
TCGA-CDR	The Cancer Genome Atlas Clinical Data Resource
TMHS	Tetraspan membrane protein of hair cell stereocilia
TPM	Transcripts per million
Treg	Regulatory T cell
TSS	Transcription start site
UCSC	University of California, Santa Cruz
UMAP	Uniform manifold approximation and projection
UMI	Unique molecular identifier
VIF	Variance inflation factor
