# Supplementary material for: Integrative Molecular Profiling and Single-Cell Characterization of LHFPL5 Reveal Cancer-Specific Regulatory Features in Clear Cell Renal Cell Carcinoma and Lung Adenocarcinoma
Source: Medicina (Kaunas). 2026 Oct 12;62(10):1971. doi: 10.3390/medicina62101971 (PMC13706886; doi:10.3390/medicina62101971)
Supplement: Supplementary file 1 [file medicina-62-01971-s001.zip › medicina-4532327-supplementary.pdf]

Supplementary Materials for the Focused LHFPL5 Study

Survival and Molecular Associations of *LHFPL5* in Clear Cell Renal Cell Carcinoma and Lung Adenocarcinoma

These materials provide the detailed methods, full numerical outputs, cohort and export definitions, model diagnostics, and supporting figures for the focused KIRC/LUAD study. Tables S1–S12 are complete electronic table collections; the compact tables below identify their contents and key denominators. Supplementary_File_Index.csv maps every electronic component to its original source and checksum.

Supplementary Methods

SM1. Study design and patient matching

KIRC and LUAD were selected for focused analysis because the preceding pan-cancer screen suggested adverse OS associations. This selection was retrospective; the focused analyses were not a prospectively specified prognostic validation study. Data were accessed on 23 September 2026. STAR-derived RNA-seq TPM matrices and clinical metadata were obtained from the UCSC Xena GDC hub. The RNA files were already transformed as log2(TPM+1), used GENCODE v36/GRCh38 identifiers, and carried the Xena version string "05-10-2024." Their metadata linked to GDC release 40. A separately retrieved current GDC service status was not used to relabel the downloaded RNA release. OS was obtained from the TCGA Clinical Data Resource (TCGA-CDR). [9, 7]

Only primary tumors were included. When multiple primary sample barcodes occurred for a patient, the lexicographically first was retained independently of expression and outcome. Aliquots had already been collapsed within the Xena sample representation. Modalities were joined by patient barcode after this rule was applied within each source. *LHFPL5* expression was standardized using the mean and sample SD among all eligible unique primary tumors within each cancer, with these constants retained across nested models. Complete-case populations were defined separately for each model family; no imputation was performed. Patients with zero, missing, or invalid OS follow-up were excluded from survival analyses.

ABSOLUTE purity, GISTIC copy-number estimates, published CIBERSORT relative fractions, DNA methylation-derived leukocyte fractions, mRNA subtypes, and published Bindea immune signature scores were obtained from GDC-hosted Pan-Cancer Atlas resources. Individual 450K methylation probes were retrieved from the Xena legacy TCGA hub and checked against GPL13534 annotations (GRCh37). RNA and methylation coordinates were not intersected across builds; integration used verified gene/probe identifiers and patient barcodes. Sources, sample flow, model populations, and version records are provided in Supplementary Tables S1 and S12. These are patient-matched modalities; identical tissue aliquots cannot be assumed. [10, 11, 12, 6, 13]

The study used existing public datasets without participant recruitment or new biospecimen collection. Overall survival (OS) was the only outcome retained for the focused survival analysis; death from any cause was the event. The earlier disease-free survival (DFS) analyses were withdrawn because their duplicated panels did not provide verifiable cohort-specific evidence. DFS was not reconstructed or relabeled as another TCGA endpoint.

SM2. Survival models and diagnostic analyses

The primary endpoint was OS. The primary exposure was continuous *LHFPL5* expression, with HRs reported per SD. Cox models used Efron handling of tied events. Model 0 included *LHFPL5* alone; Model 1 added age per 10 years and sex (male versus female); Model 2 added pathological stage (II, III, and IV versus I); and Model 3 added continuous ABSOLUTE purity. Models 0–2 were compared on the same clinical complete cases, and Models 0–3 on the same purity-complete cases. A maximal univariable analysis was retained separately.

Additional models considered KIRC grade (III/IV versus I/II), KIRC mRNA subtype, and LUAD smoking exposure per 20 pack-years when coverage and event counts were adequate. Additional full models required approximately ten deaths per fitted coefficient.

Proportional hazards (PH) were assessed using scaled Schoenfeld residuals, covariate-specific rank-time tests, and a joint Efron risk-set score test for time interactions. Diagnostic plots were examined. Stage and sex stratification addressed categorical PH violations; remaining flags were investigated with counting-process models allowing selected coefficients to differ before and after three years. Natural cubic splines with three degrees of freedom, quadratic terms, and exclusion of the highest expression percentile assessed functional-form and influence sensitivity. Primary Cox coefficients were independently checked using statsmodels. Secondary Kaplan–Meier curves used the median across all unique eligible primary tumors in each cancer, with values above the median assigned to the high group and no optimized cutpoint.

All Cox estimates are accompanied by model-specific sample sizes, deaths, 95% Wald confidence intervals, and two-sided Wald P values. Log-rank P values refer only to the Kaplan–Meier group comparisons. Binary-expression Cox estimates are retained as secondary sensitivity analyses in Supplementary Table S2 and are not interchangeable with continuous per-SD HRs. Missing covariates were not imputed. Reliable treatment covariates and LUAD molecular-subtype annotations were unavailable; their potential confounding effects could not be assessed.

SM3. Stage grade and molecular subtype

Stage, grade, and subtype differences in expression were evaluated by Kruskal–Wallis tests, with group sizes, medians, and epsilon-squared effect sizes. The four global tests formed one BH family. Pairwise subtype comparisons were performed only after a significant global test and corrected across the six comparisons. Stage/grade attenuation was evaluated within matched patient subsets; changes in HR were not interpreted as causal mediation.

SM4. Transcriptome associations and gene set enrichment

Within each cancer, gene-level log2(TPM+1) expression was modeled against standardized *LHFPL5*, age, sex, stage, and purity using ordinary least squares with HC3 heteroskedasticity-robust standard errors. Genes required TPM $\geq$1 in at least 20% of the modeling population. *LHFPL5* was excluded from the ranked gene list. Gene-level two-sided tests used t statistics with residual degrees of freedom; BH correction was applied within each cancer. These are normalized-expression association models, not count models and not evidence that *LHFPL5* regulates the identified genes.

Supplementary Table S3 also contains the completed, unadjusted transcriptome-wide Spearman analysis using all unique primary tumors (533 KIRC and 516 LUAD), with BH correction within cancer. These correlations are reported separately from the clinically and purity-adjusted gene models used for enrichment.

Preranked GSEA used MSigDB Hallmark v2026.1.Hs, signed HC3 t statistics, GSEApy 1.3.1, 10,000 gene-set permutations, seed 20260923, and gene-set sizes of 15–500. Duplicate gene symbols were resolved by highest mean expression, independently of significance. All 50 Hallmark sets were retained. For a conservative two-sided reporting convention, the software's directional nominal P was doubled, with a tail floor of 1/10001 and an upper bound of one, followed by BH correction across 50 sets per cancer. This executed convention differs from the initial protocol's proposed absolute-NES permutation calculation; both original GSEApy statistics and the reported two-sided/BH values are supplied. Leading-edge genes are included for every set. The reported BH values and the conventional GSEA permutation-based FDR q values are distinct statistics. Both are retained; a pathway meeting the former but not the latter is described as method-dependent rather than uniformly supported. [14, 15, 16, 17]

SM5. Pathway scores and survival integration

Up to three Hallmark sets per cancer were selected by absolute NES among sets with BH q <0.05, before examining pathway survival. This identified two sets in KIRC and three in LUAD. ssGSEA used sample-wise expression ranks, and scores were standardized. Spearman correlations with *LHFPL5* and separate pathway-only and combined Cox models adjusted for age, sex, stage, and purity were fitted on identical complete cases. BH correction was applied within each selected-pathway family. Rank-based enrichment and sample-score correlation were interpreted separately when directions differed.

SM6. Tumor purity and immune composition

Purity-adjusted immune associations used Spearman partial correlations obtained by residualizing ranked variables on ranked ABSOLUTE purity. Two-sided tests and approximate Fisher-z confidence intervals were reported. BH correction covered 22 CIBERSORT fractions plus leukocyte fraction per cancer. CIBERSORT P <0.05 provided a quality sensitivity analysis. Published Bindea scores supplied a complementary 25-signature analysis, corrected within cancer; these scores are not additional patient cohorts or a second set of cell fractions. The two strongest FDR-significant primary immune associations per cancer were selected by absolute correlation before survival modeling. Feature-only and combined Cox models used the same patients, and immune-feature survival tests were corrected within their model families. No validated stromal score was available for this execution. The Bindea signatures were taken from the published immune resource [18, 6].

SM7. Methylation copy number and integrated expression models

All 11 candidate probes returned by the *LHFPL5* Xena mapping were examined; ten agreed with the original *LHFPL5* manifest assignment and one annotation-discordant probe was explicitly flagged. Probe ID, chromosome, GRCh37 position, gene region, and CpG-island context were retained. Spearman methylation–expression correlations used matched primary tumors only, with BH correction across the 11-probe candidate family. Separate HC3 sensitivity regressions added copy number, purity, age, sex, and stage. GISTIC continuous copy ratios were correlated with *LHFPL5*; deposited categorical states were compared using Kruskal–Wallis tests.

The focused analysis retained the original 11-candidate BH family, including the annotation-discordant cg18451957 result for transparency. The latter is assigned to SRPK1 in the original manifest and is excluded from verified *LHFPL5* biological inference. A completed LUAD annotation sensitivity analysis applied BH correction to the ten manifest-confirmed *LHFPL5* probes. The family choice and both adjusted P values are reported explicitly in Supplementary Table S7; the threshold-sensitive promoter result is not presented as robust.

Because no individual promoter probe met the initial correlation FDR criterion, a post hoc annotation-defined mean of seven TSS probes was used for exploratory integrated expression models, requiring at least 80% observed probes. Standardized *LHFPL5* expression was regressed on this mean, copy ratio, purity, age, sex, and stage. HC3 confidence intervals, R^2, adjusted R^2, variance inflation factors, residual diagnostics, and incremental R^2 on identical patients were reported. This averaging extension was not selected using survival outcomes. Selected methylation features were also evaluated in survival models when event counts permitted.

SM8. Independent single cell localization

Three distinct non-TCGA single-cell datasets were evaluated: GSE159115 for ccRCC, GSE131907 for primary LUAD, and KRA accession KAP241022 for primary LUAD [19, 20, 21]. GSE159115 was represented by two exports of the same underlying seven-patient cohort; these exports are not independent cohorts and were not pooled. The original focused analysis used 20,748 ccRCC cells and 45,149 cells from eleven GSE131907 patients. The subsequent curated CELLxGENE exports contained 20,509 ccRCC cells from the same seven GSE159115 patients and 117,266 KAP241022 cells from eighteen treatment-naive patients. Benign kidney, chromophobe RCC, and renal metastases misclassified by anatomical site were excluded from the ccRCC/LUAD comparison. Dataset and export counts are distinguished in Supplementary Table S9.

For the original focused analysis, raw UMI counts were normalized to counts per 10,000 and log1p-transformed. Published tumor, tumor-associated epithelial, immune, vascular, and other compartment labels were retained. Detection required at least one *LHFPL5* UMI. Patients were the inferential units: paired compartment tests required at least five eligible patient pairs, sufficient compartment cells, at least twenty positive cells across the comparison, and at least five nonzero paired differences. Two-sided paired Wilcoxon tests were reported only when these criteria were met.

The subsequent localization analysis reused author quality control, labels, and embeddings from the curated exports accessed on 24 September 2026. Raw integer UMI counts were extracted from raw.X for ccRCC and X for LUAD, with *LHFPL5* uniquely mapped to ENSG00000197753. Normalized expression was ln[1 + 10,000 × *LHFPL5* UMI / total exported UMI per cell]. All exported cells were retained without new clustering, imputation, batch correction, or ambient-RNA correction. Detection at greater than zero UMI and a sensitivity threshold of at least two UMIs were summarized by annotated population and donor. Kidney t-SNE and lung UMAP coordinates were supplied by the authors; the embeddings are not comparable axes.

Kidney Tumor labels were retained. The KAP241022 study performed malignancy inference, but per-cell malignancy calls were absent from the analyzed export, so its epithelial cells were not reclassified as malignant. These subsequent comparisons are descriptive and use no cell-level significance testing. Independent refers to the origin of the single-cell cohorts relative to TCGA; localization does not independently validate bulk differential expression, survival, methylation, immune associations, or drug response.

SM9. External cohorts and RNA processing sensitivity

External OS analyses used GSE29609 (KIRC; GPL1708 probe 41466/A_32_P494620) and GSE72094 (LUAD; GPL15048 probe merck-NM_182548_at) [22, 23]. GSE68465 was excluded before outcome testing because its GPL96 annotation contained no *LHFPL5*, TMHS, Entrez 222662, or NM_182548 match [24]. This was an annotation-based measurability decision; probe-sequence reannotation was not performed. Deposited normalized expression was retained without another logarithm and standardized using all measured samples in each cohort. Cox models used Efron handling of tied deaths, 95% Wald intervals, and two-sided nominal tests. Age, sex, and categorical stage were included when available and the retained ten-deaths-per-coefficient guard was met. This guard is a modeling convention rather than proof of adequate power.

External PH diagnostics used scaled Schoenfeld rank tests and a joint Efron rank-time score test. Categorical PH departures were examined using stratification. GSE29609 had only seventeen deaths, no deposited sex or directly usable overall-stage field, and insufficient events even for the planned age-only adjusted model. No adjusted estimate was produced. Complete-case and stage-stratified GSE72094 results are reported in Supplementary Table S10.

The completed processing comparison matched TCGA STAR-derived log2(TPM+1) and legacy log2(RSEM normalized count+1) by patient and primary-tumor sample type. Spearman and Pearson correlations and median-group agreement described expression concordance. Identical-patient Cox models used each pipeline standardized within the common expression-matched cohort; those constants differ from the primary-model constants only when the eligible expression population differs. Legacy identifiers did not establish vial or aliquot identity. These analyses evaluate combined quantification, annotation, processing, and possible specimen differences, rather than an isolated aligner effect. They reuse TCGA patients and are processing-sensitivity analyses, not external validation (Supplementary Table S11).

SM10. Statistical interpretation and reproducibility

Tests were two-sided. BH families were defined by analysis domain and cancer, except the explicitly stated four-test severity and copy-number families and two-test purity family. Single prespecified exposure coefficients and PH diagnostic tests were not unnecessarily FDR corrected. All focused pathway, immune, and regulatory analyses remain exploratory because cancer and feature selection occurred within a discovery framework. Selection files preceded downstream feature-survival analyses, but this does not eliminate same-

cohort selection bias. Python 3.12.14, lifelines 0.30.3, statsmodels 0.15.0, SciPy 1.18.1, and GSEApy 1.3.1 were used. Scripts, package/session versions, analysis-ready extracts, source records, model-specific patient lists, and complete statistical outputs are supplied in Supplementary Tables S1–S12 and the accompanying reproducibility files. No new biological analysis was performed during manuscript assembly.

TIMER2.0, GEPIA2, and GSCA analyses using TCGA represent cross-platform consistency, not independent patient validation. The earlier broad portal, CancerSEA, pan-cancer mutation, and GDSC/CTRP drug-correlation analyses were not retained as evidence in this focused study. No treatment-prediction, calibration, or experimental mechanism was tested.

Supplementary table collections

Table S1. Cohort flow clinical characteristics and matching

Counts refer to the stated analysis population. Clinical and purity subsets are nested. Stage/grade/subtype tests use expression-complete populations, which differ from OS-complete populations. Patient-level membership and missingness are supplied separately.

Component file	Contents
Table_S1_01.tsv	S1 covariate missingness.tsv
Table_S1_02.tsv	S1 multiomics flow.tsv
Table_S1_03.tsv	S1 sample flow.tsv
Table_S1_04.tsv	Table1 clinical characteristics.tsv
Table_S1_05.tsv	Table1 clinical summaries.tsv
Table_S1_06.tsv	clinicopathological global.tsv
Table_S1_07.tsv	clinicopathological pairwise.tsv

Sample-flow step	KIRC N	LUAD N
RNA_samples	610	589
RNA_primary_samples	537	528
unique_primary_patients	533	516
valid_OS	531	503
clinical_complete	528	485
purity_complete	324	462

Table S2. Complete survival models and diagnostics

HRs are model-specific per-SD effects unless explicitly labeled binary high versus low. All available covariate coefficients, events, Wald intervals, two-sided P values, global/covariate PH tests, matched base models, stratification, time-varying terms, splines, and influence analyses are retained. Source-defined model labels are preserved in the machine-readable files.

Component file	Contents
Table_S2_01.tsv	KIRC spline curve.tsv

Component file	Contents
Table_S2_02.tsv	KM results.tsv
Table_S2_03.tsv	LUAD spline curve.tsv
Table_S2_04.tsv	S2 PH tests.tsv
Table_S2_05.tsv	S2 S10 additional stratified global PH.tsv
Table_S2_06.tsv	S2 S5 timevarying Cox.tsv
Table_S2_07.tsv	S2 S5 timevarying effects.tsv
Table_S2_08.tsv	S2 complete Cox results.tsv
Table_S2_09.tsv	S2 extended Cox results.tsv
Table_S2_10.tsv	S2 extended PH tests.tsv
Table_S2_11.tsv	S2 matched processing Cox.tsv
Table_S2_12.tsv	S2 processing and influence Cox.tsv
Table_S2_13.tsv	S2 processing and influence PH.tsv
Table_S2_14.tsv	Table2 primary Cox.tsv
Table_S2_15.tsv	nonlinearity tests.tsv
Table_S2_16.json	survival diagnostics.json
Table_S2_17.csv	Table R1 Univariable Multivariable Cox.csv
Table_S2_18.tsv	Kaplan Meier number at risk.tsv
Table_S2_19.json	Survival summary.json

Table S3. Transcriptome wide associations and correlations

Adjusted gene models use normalized expression and HC3 uncertainty with age, sex, stage, and purity. Unadjusted Spearman correlations use all unique primary tumors and are labeled separately. Neither analysis identifies genes regulated by *LHFPL5*. Full outputs are provided without truncating nonsignificant genes.

Component file	Contents
Table_S3_01.tsv	KIRC top associated genes.tsv
Table_S3_02.tsv	LUAD top associated genes.tsv
Table_S3_03.tsv.gz	S3 KIRC all gene associations.tsv.gz
Table_S3_04.tsv.gz	S3 LUAD all gene associations.tsv.gz
Table_S3_05.tsv	transcriptome counts.tsv
Table_S3_06.csv	Gene Correlation Counts.csv
Table_S3_07.csv.gz	Table R2 KIRC All Gene Correlations.csv.gz
Table_S3_08.csv.gz	Table R2 LUAD All Gene Correlations.csv.gz

Component file	Contents
Table_S3_09.csv	Table R2 Strongest Correlated Genes.csv

Table S4. All Hallmark enrichment results

All fifty sets per cancer are retained. BH_q is the archived doubled-nominal-P convention; FDR q-val is the distinct conventional GSEA statistic. Leading-edge genes and ranking files are supplied. Finite-permutation zero values must not be interpreted as probabilities of exactly zero.

Component file	Contents
Table_S4_01.tsv	S4 all Hallmark GSEA.tsv

Cancer and set	NES	BH q	Conventional FDR
KIRC REACTIVE OXYGEN SPECIES PATHWAY	-2.093	0.005	0.000
KIRC ANGIOGENESIS	-1.950	0.005	0.037
LUAD PANCREAS BETA CELLS	-1.623	0.010	0.000
LUAD MITOTIC SPINDLE	1.319	0.010	0.267
LUAD G2M CHECKPOINT	1.274	0.017	0.258

Table S5. Pathway scores and survival models

Selected pathway-only and combined models use identical complete cases. Scores are standardized. Changes in expression HR are statistical overlap, not mediation. Selection occurred in the same cohorts, before downstream pathway-survival testing, and remains exploratory.

Component file	Contents
Table_S5_01.tsv	S5 S6 stratified extended Cox.tsv
Table_S5_02.tsv	S5 S6 stratified extended PH.tsv
Table_S5_03.tsv	S5 extended PH tests.tsv
Table_S5_04.tsv	S5 pathway and methylation Cox.tsv
Table_S5_05.tsv	S5 pathway correlations.tsv
Table_S5_06.tsv	Table3 pathway survival.tsv

Table S6. Purity and immune composition analyses

Primary correlations are purity-adjusted Spearman estimates for twenty-two relative CIBERSORT fractions and leukocyte fraction; the BH family is twenty-three tests per cancer. Complementary Bindea signatures and CIBERSORT quality sensitivity reuse the same patients. Approximate Fisher-z intervals and feature-survival models are included.

Component file	Contents
Table_S6_01.tsv	S6 Bindea crossmethod.tsv
Table_S6_02.tsv	S6 KIRC CIBERSORT quality sensitivity.tsv

Component file	Contents
Table_S6_03.tsv	S6 LUAD CIBERSORT quality sensitivity.tsv
Table_S6_04.tsv	S6 crossmethod comparison.tsv
Table_S6_05.tsv	S6 immune results.tsv
Table_S6_06.tsv	S6 immune survival Cox.tsv
Table_S6_07.tsv	purity results.tsv

Table S7. Probe level methylation analyses

All eleven candidate probes, including the SRPK1-discordant cg18451957, are documented. The focused BH family uses eleven candidates. Completed LUAD ten-manifest-probe correction is separately labeled and shows threshold sensitivity for cg11529128. Primary biological inference excludes the discordant probe. GRCh37 annotation, patient matching, adjusted models, and corrected cg00740861 identifier are retained.

Component file	Contents
Table_S7_01.tsv	S7 adjusted probe sensitivities.tsv
Table_S7_02.tsv	S7 methylation probes.tsv
Table_S7_03.csv	Completed LUAD ten-probe annotation sensitivity: Complete Probe Correlation Results.csv
Table_S7_04.csv	Completed LUAD ten-probe annotation sensitivity: LHFPL5 Primary Probe Correlations.csv

Table S8. Copy number and integrated expression models

Continuous GISTIC copy ratio and categorical states are distinct analyses. Integrated models include the post hoc seven-TSS-probe mean, copy ratio, purity, age, sex, and stage. HC3 coefficients, intercepts, R^2, incremental R^2, VIF, residual, heteroskedasticity, and influence records are supplied. KIRC methylation-extended survival did not meet the event guard.

Component file	Contents
Table_S8_01.tsv	S8 copy number.tsv
Table_S8_02.tsv	Table4 integrated expression models.tsv
Table_S8_03.json	expression model diagnostics.json
Table_S8_04.json	integration uncompleted.json

Table S9. Single cell datasets exports and localization

Three underlying single-cell datasets are represented. Two GSE159115 exports overlap and are not independent replication. Original GEO-based localization and the subsequent CELLxGENE localization use their own deposited labels and denominators. Raw-UMI detection, normalized expression, cell totals, donor summaries, embedding coordinates, and threshold sensitivity are included. Most original donor contrasts were ineligible; newer comparisons are descriptive.

Component file	Contents
Table_S9_01.tsv	S9 singlecell compartments.tsv
Table_S9_02.tsv	S9 singlecell patient summaries.tsv
Table_S9_03.tsv	S9 singlecell patient tests.tsv
Table_S9_04.csv.gz	Curated CELLxGENE export: KIRC LHFPL5 cell data.csv.gz
Table_S9_05.csv	Curated CELLxGENE export: KIRC author cell statistics.csv
Table_S9_06.csv	Curated CELLxGENE export: KIRC donor cell statistics.csv
Table_S9_07.csv	Curated CELLxGENE export: KIRC major cell statistics.csv
Table_S9_08.csv	Curated CELLxGENE export: LHFPL5 donor cell statistics.csv
Table_S9_09.csv	Curated CELLxGENE export: LHFPL5 major cell statistics.csv
Table_S9_10.csv	Curated CELLxGENE export: LHFPL5 sample details.csv
Table_S9_11.csv.gz	Curated CELLxGENE export: LUAD LHFPL5 cell data.csv.gz
Table_S9_12.csv	Curated CELLxGENE export: LUAD author cell statistics.csv
Table_S9_13.csv	Curated CELLxGENE export: LUAD donor cell statistics.csv
Table_S9_14.csv	Curated CELLxGENE export: LUAD major cell statistics.csv
Table_S9_15.json	Curated CELLxGENE export: analysis manifest.json

Dataset and export	Patients	Cells	LHFPL5-positive cells
GSE159115 original GEO-derived ccRCC export	7	20,748	34
GSE159115 curated CELLxGENE ccRCC export; overlapping patients	7	20,509	34
GSE131907 primary LUAD	11	45,149	25
KAP241022 curated LUAD	18	117,266	2,480

Table S10. External clinical cohort analyses

Latest completed external models use GSE29609 and GSE72094. Total measured samples, valid OS, known vital status, and adjusted complete cases are separate populations. GSE68465 is excluded on annotation-based measurability before outcome testing. GSE29609 has no adjusted fit. All fitted coefficients, PH diagnostics, source scaling, probe records, and membership are included.

Component file	Contents
Table_S10_01.csv	Cohort eligibility.csv
Table_S10_02.csv	Cohort flow.csv
Table_S10_03.csv	Cox all coefficients.csv
Table_S10_04.csv	Cox results.csv
Table_S10_05.csv	Expression standardization.csv

Component file	Contents
Table_S10_06.csv	Figure1 plotted results.csv
Table_S10_07.csv	Model patient membership.csv
Table_S10_08.csv	PH diagnostics.csv
Table_S10_09.csv	Analysis-ready data: GSE29609.csv
Table_S10_10.csv	Analysis-ready data: GSE72094.csv

Table S11. Matched RNA processing sensitivity

Latest matched TCGA processing comparisons use identical clinical complete cases within cancer, with pipeline-specific standardization in the common expression population. These are neither independent cohorts nor an isolated aligner comparison. Primary clinical LUAD N = 485 differs from matched-pipeline N = 484. Archived influence/processing outputs are identified separately from the latest matched tables.

Component file	Contents
Table_S11_01.tsv	processing sensitivity.tsv
Table_S11_02.csv	FigureS1 plotted results.csv
Table_S11_03.csv	Processing Cox results.csv
Table_S11_04.csv	Processing correlations.csv
Table_S11_05.csv	Analysis-ready data: TCGA KIRC.csv
Table_S11_06.csv	Analysis-ready data: TCGA KIRC matched expression.csv
Table_S11_07.csv	Analysis-ready data: TCGA LUAD.csv
Table_S11_08.csv	Analysis-ready data: TCGA LUAD matched expression.csv

Table S12. Provenance software and completion records

Source records retain original analysis dates, release labels, software versions, checksums, analysis protocol and deviations. Completion registers record unavailable components, not negative findings. The assembly index maps source files to final supplement identifiers. No biological statistical model was rerun for this revision.

Component file	Contents
Table_S12_01.tsv	ANALYSIS COMPLETION.tsv
Table_S12_02.json	RNA input verification.json
Table_S12_03.json	final quality checks.json
Table_S12_04.tsv	verification checks.tsv
Table_S12_05.tsv	source inventory.tsv
Table_S12_06.json	analysis protocol.json
Table_S12_07.json	session info.json
Table_S12_08.md	README.md

Component file	Contents
Table_S12_09.md	ANALYSIS LOG.md

References

1. Xiong, W.; Grillet, N.; Elledge, H.M.; Wagner, T.F.J.; Zhao, B.; Johnson, K.R.; Kazmierczak, P.; Müller, U. TMHS is an integral component of the mechanotransduction machinery of cochlear hair cells. Cell 2012, 151, 1283–1295. doi:10.1016/j.cell.2012.10.041.

2. Qiu, X.; Liang, X.; Llongueras, J.P.; Cunningham, C.; Müller, U. The tetraspan *LHFPL5* is critical to establish maximal force sensitivity of the mechanotransduction channel of cochlear hair cells. Cell Rep. 2023, 42, 112245. doi:10.1016/j.celrep.2023.112245.

3. Gong, X.; Liu, Y.; Zhang, Q.; Liang, K.; Wei, J.; Du, H. LHFPL2 serves as a potential biomarker for M2 polarization of macrophages in renal cell carcinoma. Int. J. Mol. Sci. 2024, 25, 6707. doi:10.3390/ijms25126707.

4. Liu, Y.J.; Yin, S.Y.; Zeng, S.H.; Hu, Y.D.; Wang, M.Q.; Huang, P.; Li, J.P. Prognostic value of LHFPL tetraspan subfamily member 6 in gastric cancer: A study based on bioinformatics analysis and experimental validation. Pharmgenomics Pers. Med. 2021, 14, 1483–1504. doi:10.2147/PGPM.S332345.

5. Dagogo-Jack, I.; Shaw, A.T. Tumour heterogeneity and resistance to cancer therapies. Nat. Rev. Clin. Oncol. 2018, 15, 81–94. doi:10.1038/nrclinonc.2017.166.

6. Thorsson, V.; Gibbs, D.L.; Brown, S.D.; Wolf, D.; Bortone, D.S.; Ou Yang, T.H.; Porta-Pardo, E.; Gao, G.F.; Plaisier, C.L.; Eddy, J.A.; et al. The immune landscape of cancer. Immunity 2018, 48, 812–830.e14. doi:10.1016/j.immuni.2018.03.023.

7. Liu, J.; Lichtenberg, T.; Hoadley, K.A.; Poisson, L.M.; Lazar, A.J.; Cherniack, A.D.; Kovatich, A.J.; Benz, C.C.; Levine, D.A.; Lee, A.V.; et al. An integrated TCGA pan-cancer clinical data resource to drive high-quality survival outcome analytics. Cell 2018, 173, 400–416.e11. doi:10.1016/j.cell.2018.02.052.

8. Baylin, S.B.; Jones, P.A. Epigenetic determinants of cancer. Cold Spring Harb. Perspect. Biol. 2016, 8, a019505. doi:10.1101/cshperspect.a019505.

9. Goldman, M.J.; Craft, B.; Hastie, M.; Repečka, K.; McDade, F.; Kamath, A.; Banerjee, A.; Luo, Y.; Rogers, D.; Brooks, A.N.; et al. Visualizing and interpreting cancer genomics data via the Xena platform. Nat. Biotechnol. 2020, 38, 675–678. doi:10.1038/s41587-020-0546-8.

10. Carter, S.L.; Cibulskis, K.; Helman, E.; McKenna, A.; Shen, H.; Zack, T.; Laird, P.W.; Onofrio, R.C.; Winckler, W.; Weir, B.A.; et al. Absolute quantification of somatic DNA alterations in human cancer. Nat. Biotechnol. 2012, 30, 413–421. doi:10.1038/nbt.2203.

11. Mermel, C.H.; Schumacher, S.E.; Hill, B.; Meyerson, M.L.; Beroukhim, R.; Getz, G. GISTIC2.0 facilitates sensitive and confident localization of the targets of focal somatic copy-number alteration in human cancers. Genome Biol. 2011, 12, R41. doi:10.1186/gb-2011-12-4-r41.

12. Newman, A.M.; Liu, C.L.; Green, M.R.; Gentles, A.J.; Feng, W.; Xu, Y.; Hoang, C.D.; Diehn, M.; Alizadeh, A.A. Robust enumeration of cell subsets from tissue expression profiles. Nat. Methods 2015, 12, 453–457. doi:10.1038/nmeth.3337.

13. NCBI Gene Expression Omnibus. GPL13534: Illumina HumanMethylation450 BeadChip. Available online: https://www.ncbi.nlm.nih.gov/geo/query/acc.cgi?acc=GPL13534 (accessed on 23 September 2026).

14. Subramanian, A.; Tamayo, P.; Mootha, V.K.; Mukherjee, S.; Ebert, B.L.; Gillette, M.A.; Paulovich, A.; Pomeroy, S.L.; Golub, T.R.; Lander, E.S.; Mesirov, J.P. Gene set enrichment analysis: A knowledge-based

approach for interpreting genome-wide expression profiles. Proc. Natl. Acad. Sci. USA 2005, 102, 15545–15550. doi:10.1073/pnas.0506580102.

15. Liberzon, A.; Birger, C.; Thorvaldsdóttir, H.; Ghandi, M.; Mesirov, J.P.; Tamayo, P. The Molecular Signatures Database hallmark gene set collection. Cell Syst. 2015, 1, 417–425. doi:10.1016/j.cels.2015.12.004.

16. Fang, Z.; Liu, X.; Peltz, G. GSEApy: A comprehensive package for performing gene set enrichment analysis in Python. Bioinformatics 2023, 39, btac757. doi:10.1093/bioinformatics/btac757.

17. Broad Institute. MSigDB Human Hallmark gene sets, release 2026.1.Hs. Available online: https://data.broadinstitute.org/gsea-msigdb/msigdb/release/2026.1.Hs/h.all.v2026.1.Hs.symbols.gmt (accessed on 23 September 2026).

18. Bindea, G.; Mlecnik, B.; Tosolini, M.; Kirilovsky, A.; Waldner, M.; Obenauf, A.C.; Angell, H.; Fredriksen, T.; Lafontaine, L.; Berger, A.; et al. Spatiotemporal dynamics of intratumoral immune cells reveal the immune landscape in human cancer. Immunity 2013, 39, 782–795. doi:10.1016/j.immuni.2013.10.003.

19. Zhang, Y.; Narayanan, S.P.; Mannan, R.; Raskind, G.; Wang, X.; Vats, P.; Su, F.; Hosseini, N.; Cao, X.; Kumar-Sinha, C.; et al. Single-cell analyses of renal cell cancers reveal insights into tumor microenvironment, cell of origin, and therapy response. Proc. Natl. Acad. Sci. USA 2021, 118, e2103240118. doi:10.1073/pnas.2103240118.

20. Kim, N.; Kim, H.K.; Lee, K.; Hong, Y.; Cho, J.H.; Choi, J.W.; Lee, J.I.; Suh, Y.L.; Ku, B.M.; Eum, H.H.; et al. Single-cell RNA sequencing demonstrates the molecular and cellular reprogramming of metastatic lung adenocarcinoma. Nat. Commun. 2020, 11, 2285. doi:10.1038/s41467-020-16164-1.

21. Lee, J.; Jeong, J.Y.; Hong, M.J.; Choi, Y.H.; Kim, J.Y.; Lee, J.H.; Choi, J.E.; Kim, M.; Do, Y.W.; Lee, E.B.; et al. Defining the cellular and molecular identities of histologic subtypes in lung adenocarcinoma. Exp. Hematol. Oncol. 2026, 15, 12. doi:10.1186/s40164-025-00740-6.

22. NCBI Gene Expression Omnibus. GSE29609: Clear-cell renal cell carcinomas tumors. Available online: https://www.ncbi.nlm.nih.gov/geo/query/acc.cgi?acc=GSE29609 (accessed on 23 September 2026).

23. NCBI Gene Expression Omnibus. GSE72094: Lung adenocarcinoma gene-expression and clinical cohort. Available online: https://www.ncbi.nlm.nih.gov/geo/query/acc.cgi?acc=GSE72094 (accessed on 23 September 2026).

24. NCBI Gene Expression Omnibus. GPL96: Affymetrix Human Genome U133A Array. Available online: https://www.ncbi.nlm.nih.gov/geo/query/acc.cgi?acc=GPL96 (accessed on 23 September 2026).

25. Pickup, M.W.; Mouw, J.K.; Weaver, V.M. The extracellular matrix modulates the hallmarks of cancer. EMBO Rep. 2014, 15, 1243–1253. doi:10.15252/embr.201439246.

Figure S1. Pathological stage and expression

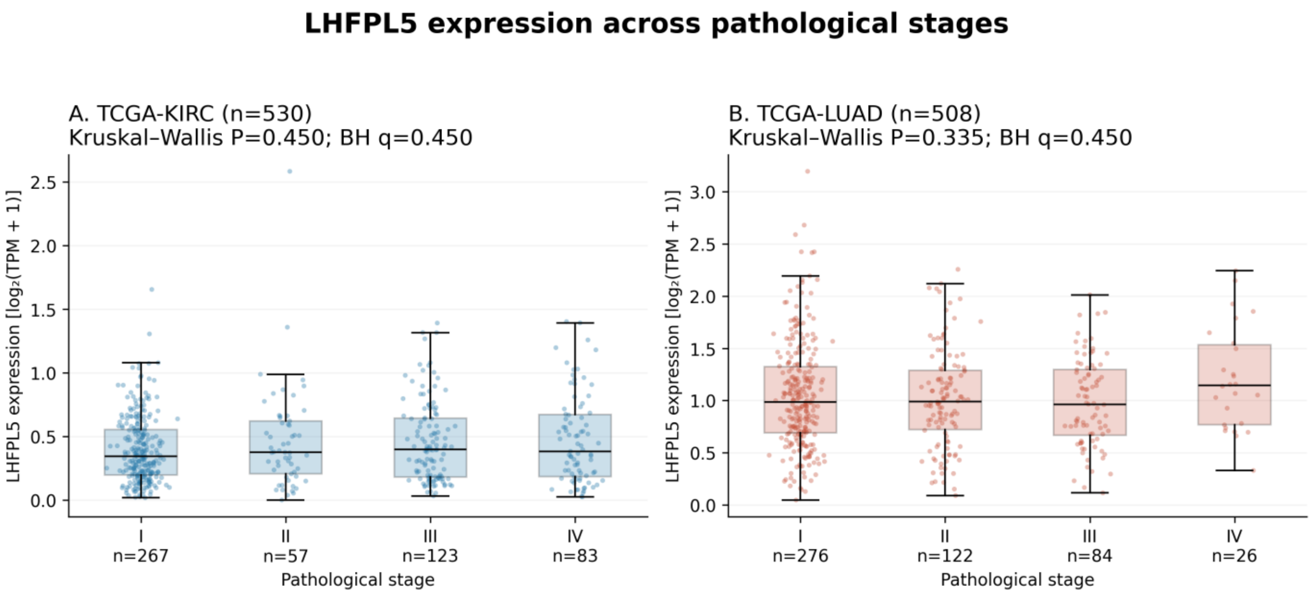

Primary STAR-derived *LHFPL5* log2(TPM+1) across pathological stages. Kruskal–Wallis P = 0.450 for KIRC and 0.335 for LUAD. Group sizes and effect sizes are in Table S1; these plots do not support a stage-associated increase.

Figure S2. KIRC transcriptome associations

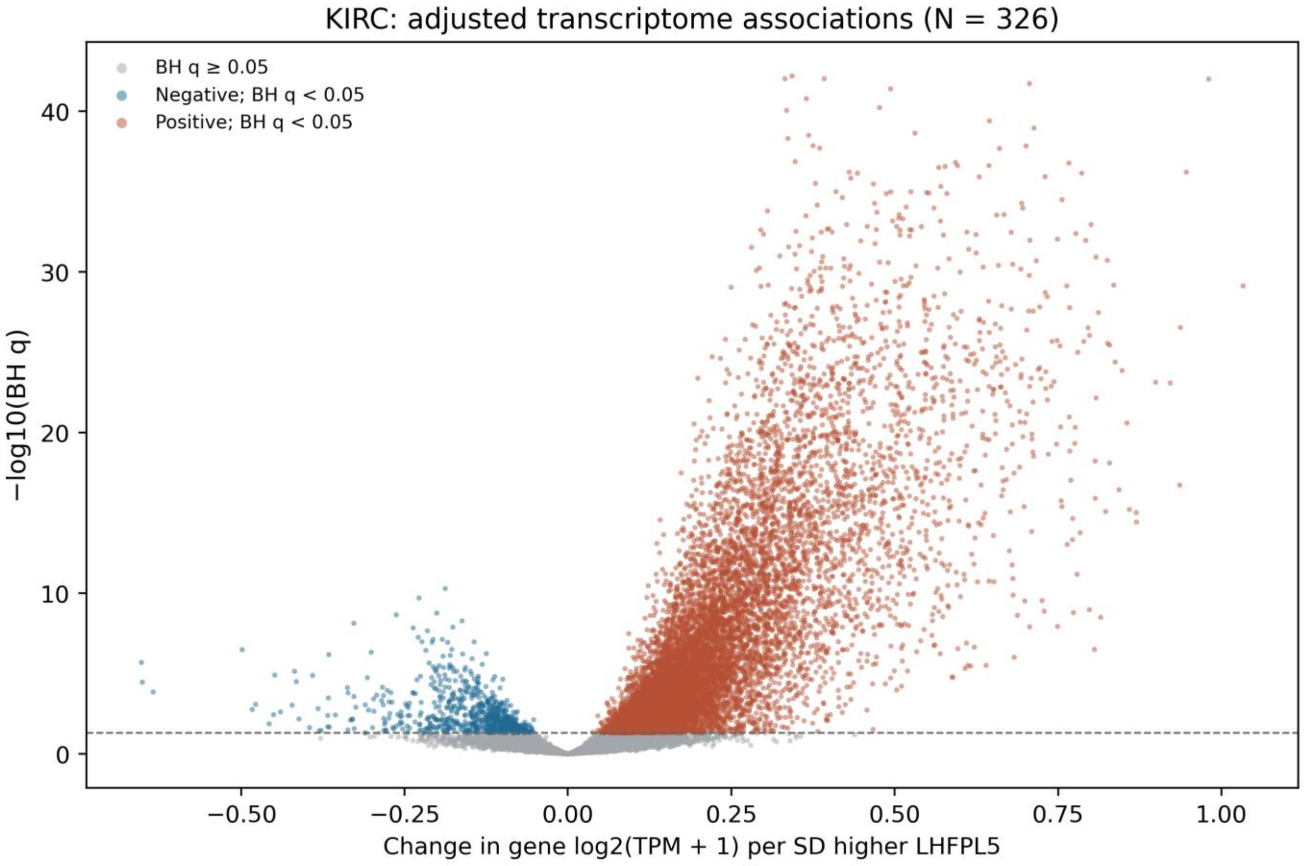

HC3 covariate-adjusted gene-expression associations with standardized *LHFPL5* in 326 KIRC patients. Models adjust for age, sex, stage, and purity. Complete coefficients, P and BH q values are in Table S3.

Figure S3. LUAD transcriptome associations

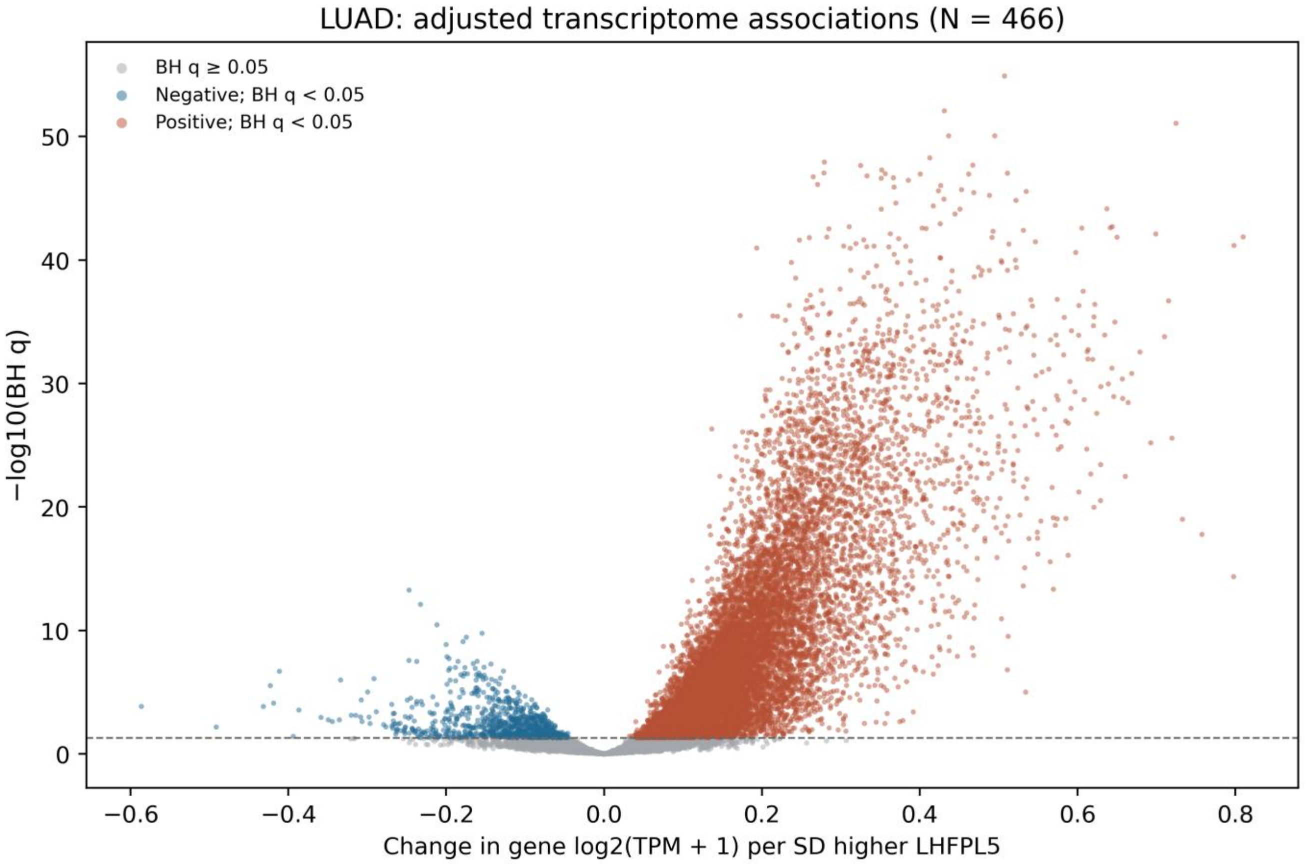

HC3 covariate-adjusted gene-expression associations with standardized *LHFPL5* in 466 LUAD patients. Models adjust for age, sex, stage, and purity. Complete outputs are in Table S3.

Figure S4. KIRC selected running enrichment curves

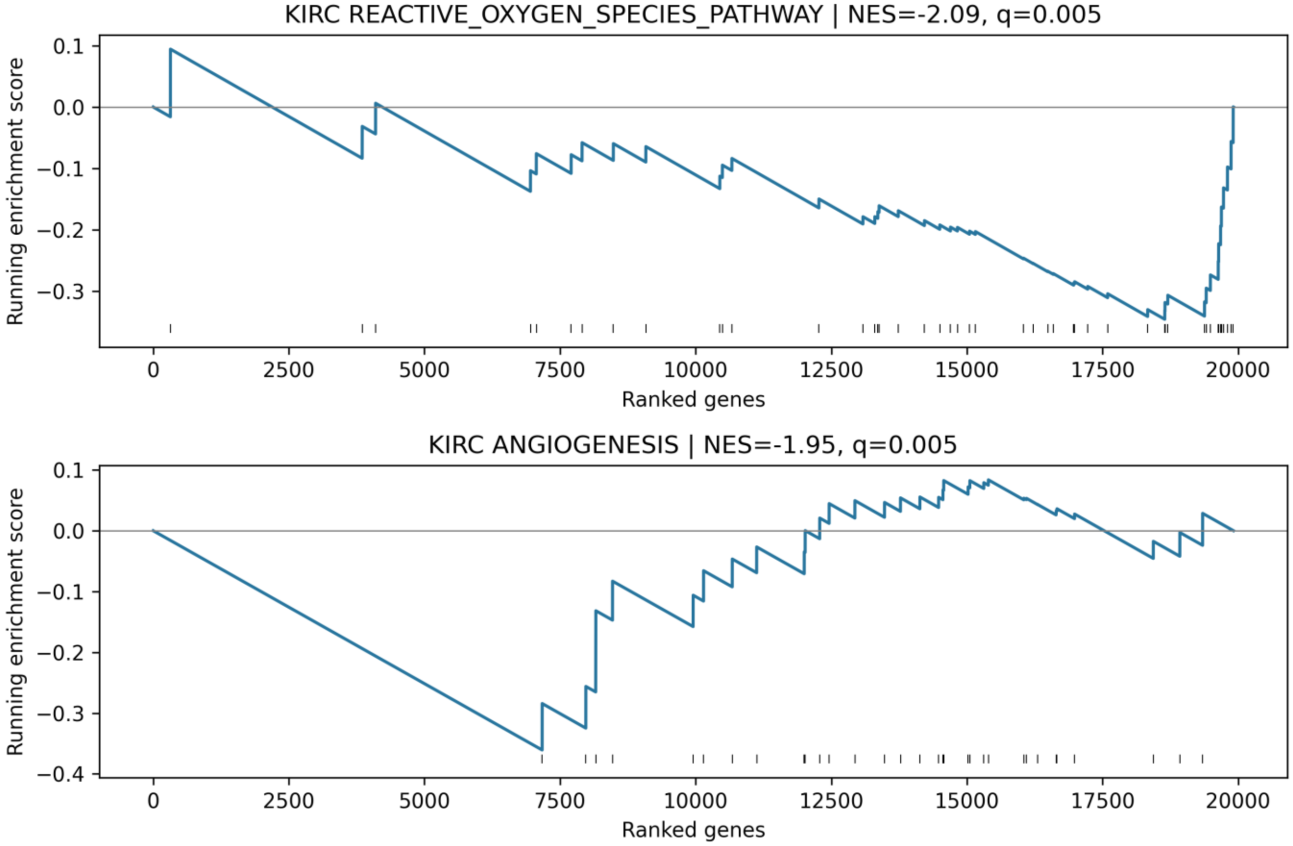

ROS and angiogenesis enrichment against signed HC3 t-statistic rankings. Tick marks denote set members. Reported q follows the archived doubled-nominal-P/BH convention. All original GSEA values and leading edges are in Table S4. Negative angiogenesis NES does not imply a negative sample-score correlation.

Figure S5. LUAD selected running enrichment curves

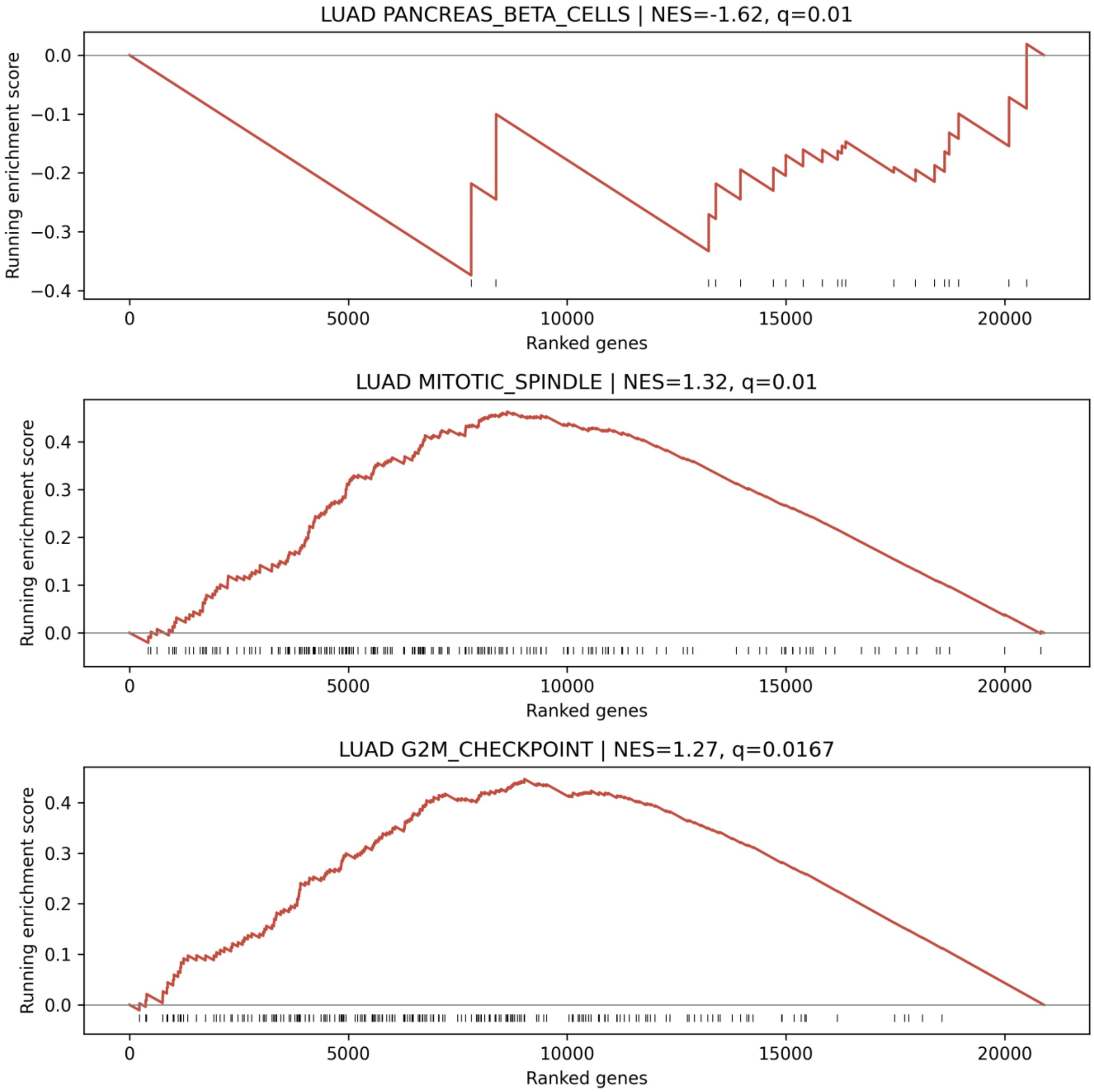

Pancreas beta-cell, mitotic-spindle, and G2M checkpoint gene sets. Reported q follows the archived doubled-nominal-P/BH convention. Conventional GSEA FDR is 0.267 for spindle and 0.258 for G2M, so those enrichment findings are method-dependent. All statistics are in Table S4.

Figure S6. Original focused single cell export results

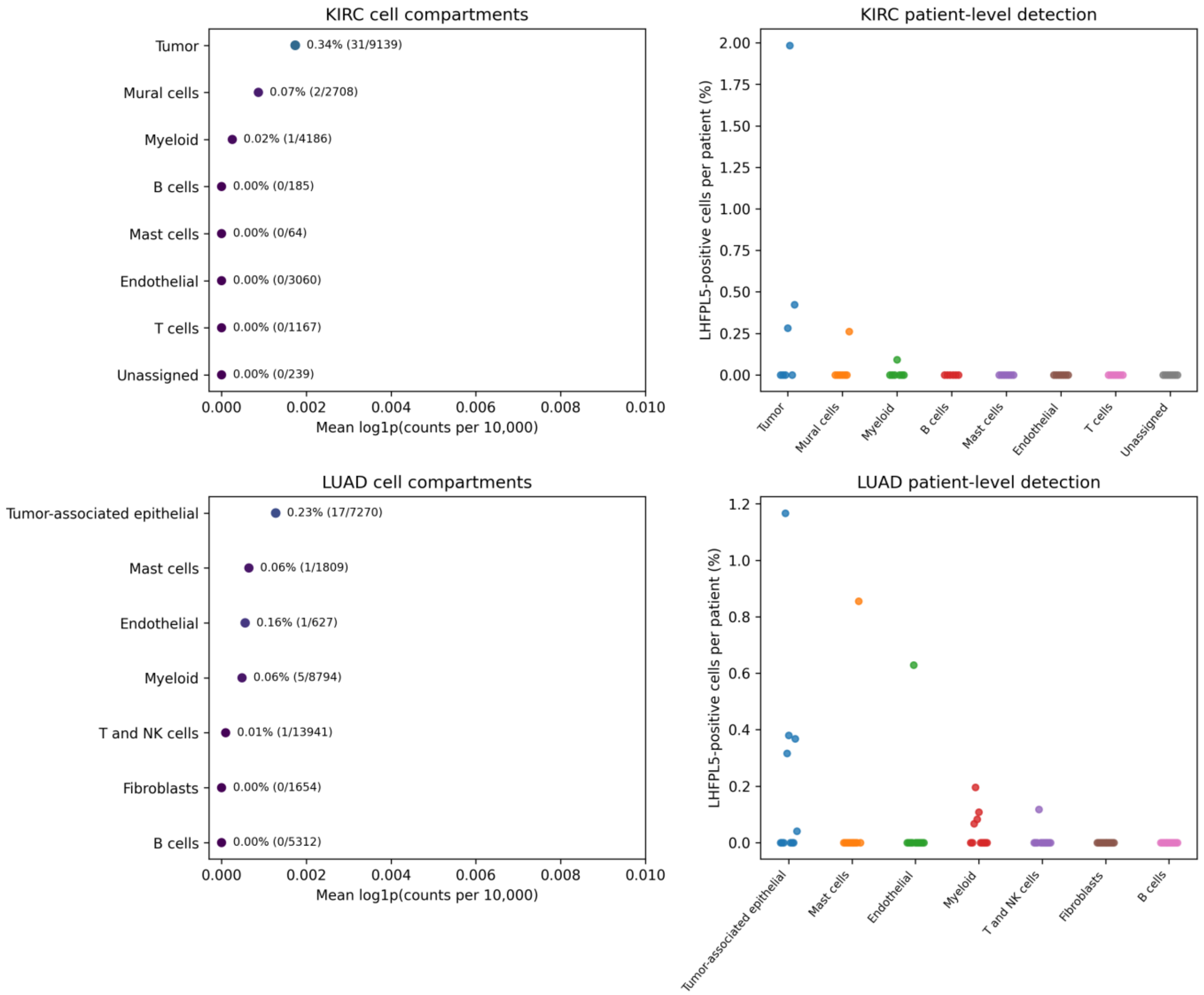

GSE159115: 20,748 cells from seven ccRCC patients, thirty-four positive cells. GSE131907: 45,149 primary LUAD cells from eleven patients, twenty-five positive cells. Positivity is at least one raw UMI. Published compartment labels were retained. Patient-level comparisons use eligible matched donors; most failed minimum detection criteria. The eligible LUAD epithelial-versus-myeloid comparison has P = 0.09375. These are sparse exploratory results. The renal cohort overlaps Figures 6–7 and is not independent replication.

Figure S7. Matched TCGA expression processing comparison

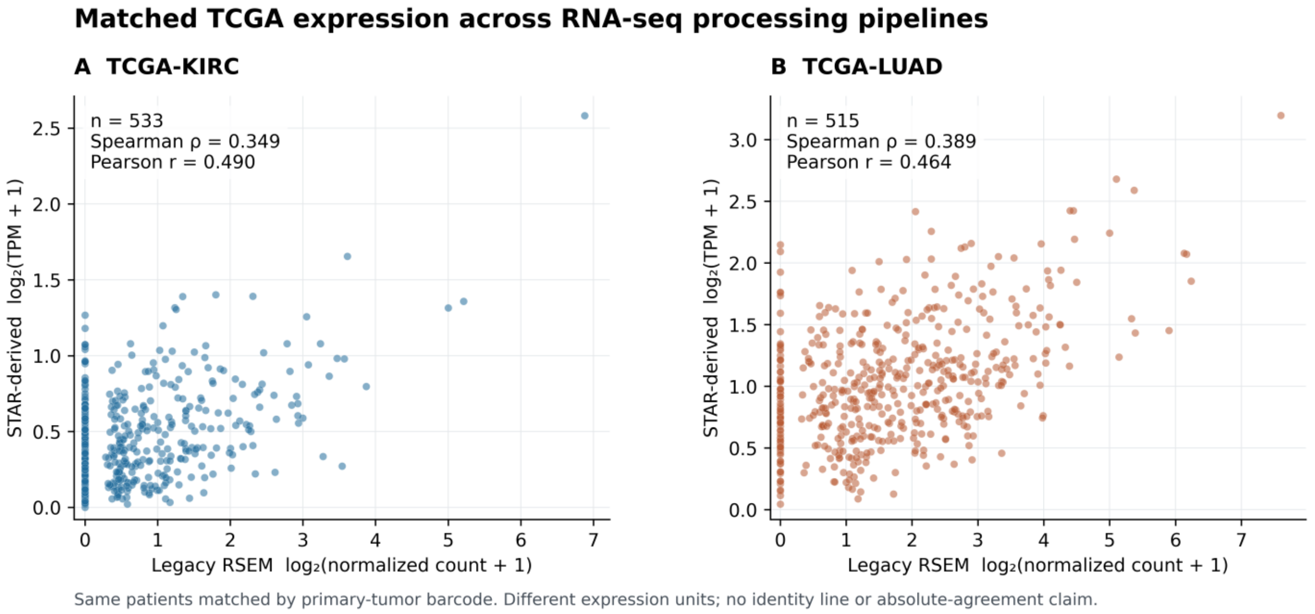

Each point is a patient represented by primary-tumor expression under STAR and legacy RSEM: 533 KIRC and 515 LUAD. Source scales are retained without a second logarithm. Spearman and Pearson coefficients describe correlation, not absolute agreement. Vial/aliquot identity is unavailable in the legacy identifiers. Table S11 contains correlations, group agreement, and scaling constants.

Figure S8. Processing sensitivity of overall survival

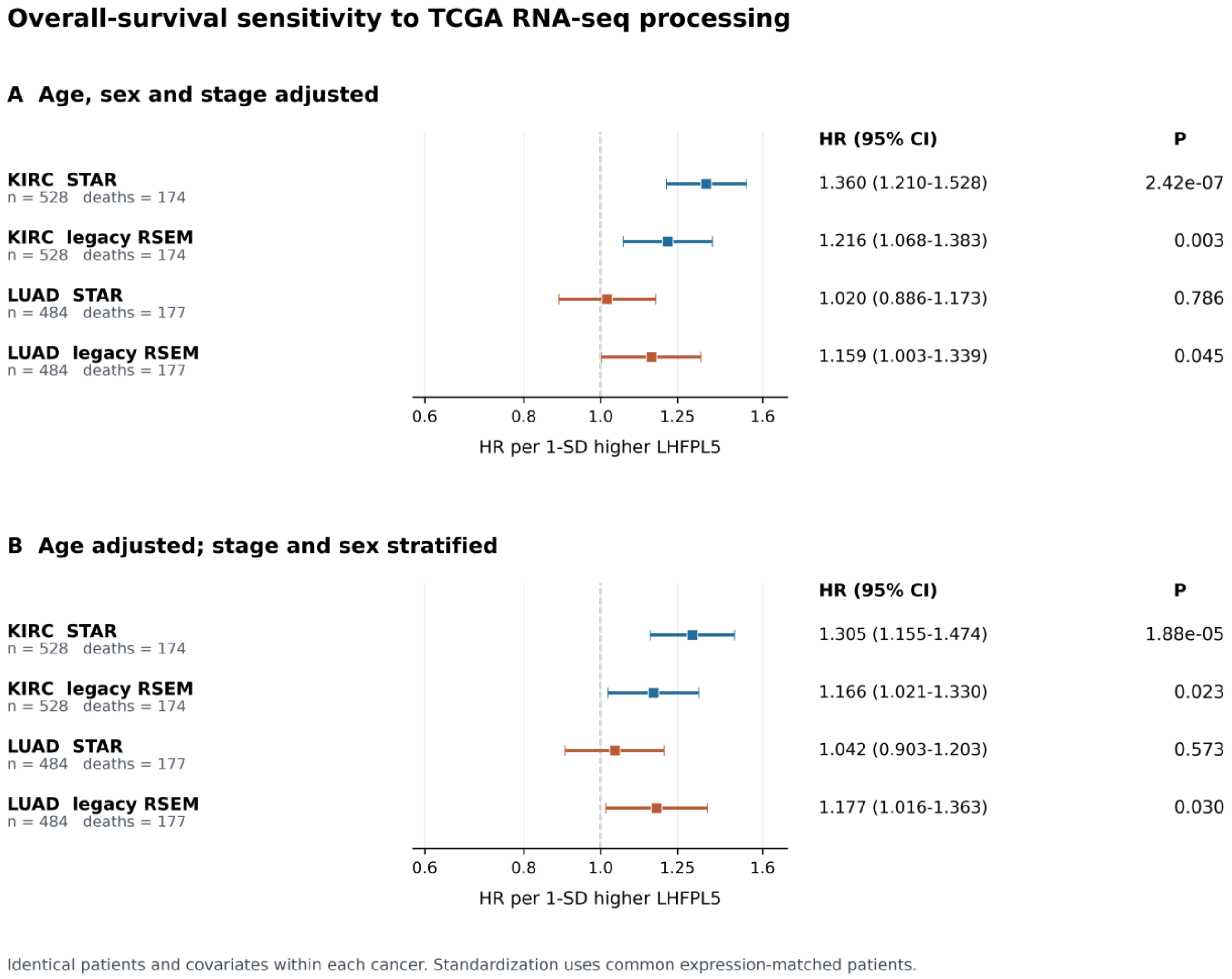

Identical clinical complete cases within each cancer: KIRC 528/174 and LUAD 484/177 patients/deaths. Panel A adjusts for age, sex, and stage; panel B adjusts for age and stratifies by stage and sex. HRs and 95% CIs use each pipeline's SD within the common expression population. Two-sided Wald P values are shown. These are repeated TCGA analyses, not external validation or a formal test that coefficients differ.

Figure S9. Primary model proportional hazards diagnostics — panel page 1

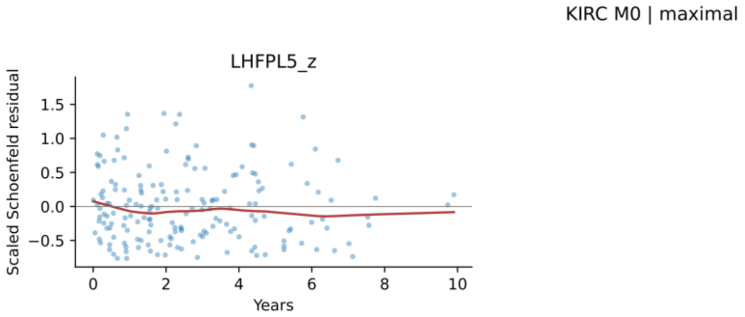

Scaled Schoenfeld diagnostic plots for primary KIRC and LUAD Cox models. Covariate and global test definitions and results are in Table S2. Significant departures are disclosed in Results 3.3; lack of significance does not prove proportionality.

Figure S9. Primary model proportional hazards diagnostics — panel page 2

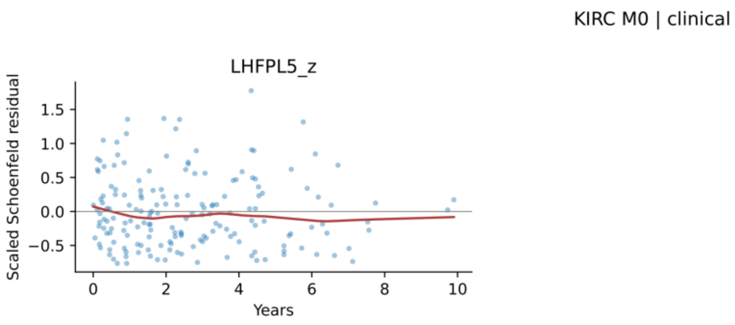

Continuation of Figure S9. See the legend on its first page.

Figure S9. Primary model proportional hazards diagnostics — panel page 3

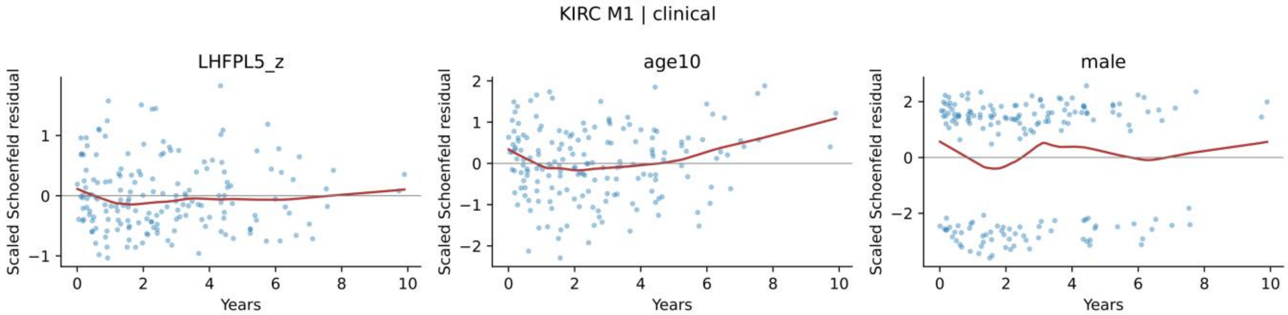

Continuation of Figure S9. See the legend on its first page.

Figure S9. Primary model proportional hazards diagnostics — panel page 4

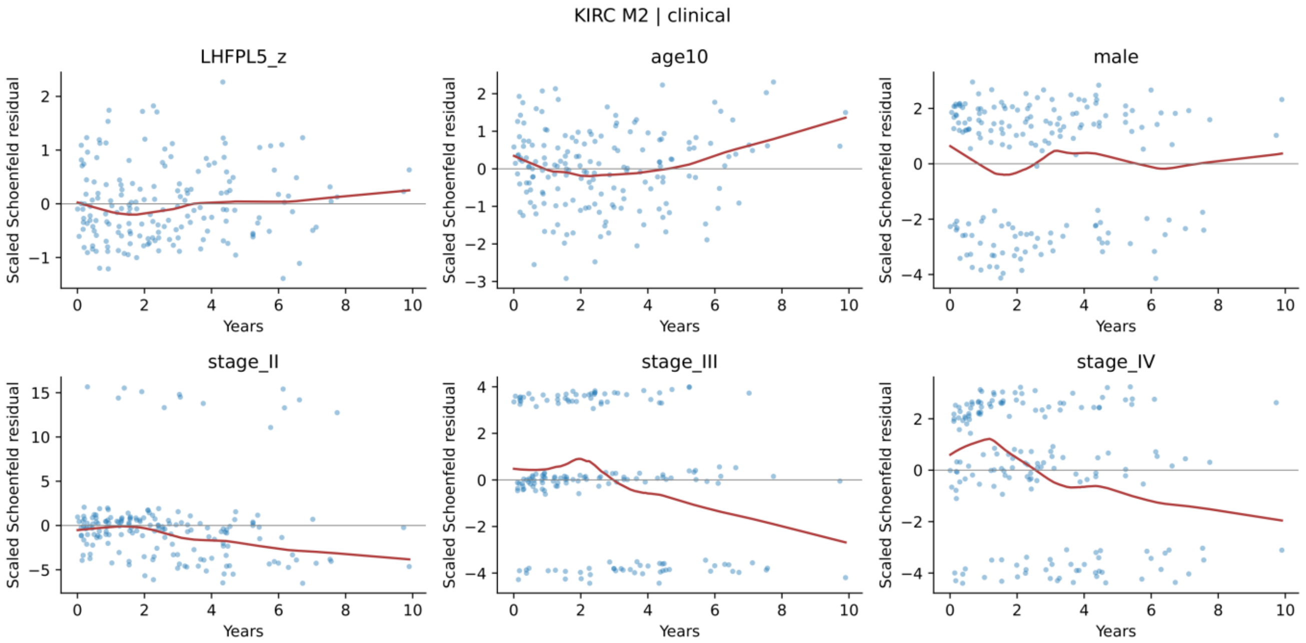

Continuation of Figure S9. See the legend on its first page.

Figure S9. Primary model proportional hazards diagnostics — panel page 5

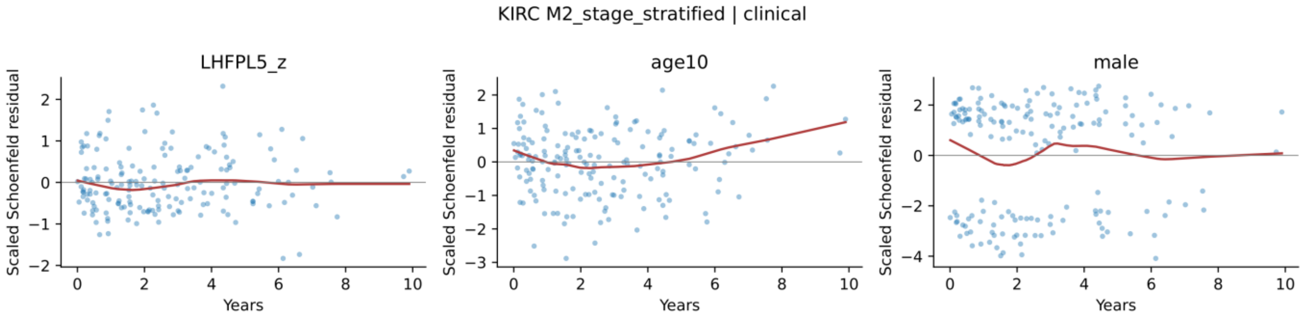

Continuation of Figure S9. See the legend on its first page.

Figure S9. Primary model proportional hazards diagnostics — panel page 6

KIRC M0 | purity

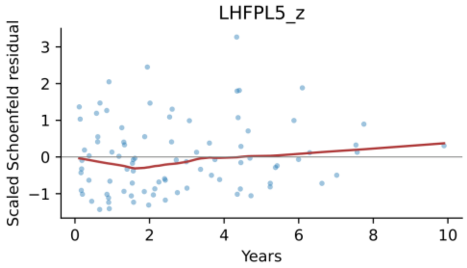

Continuation of Figure S9. See the legend on its first page.

Figure S9. Primary model proportional hazards diagnostics — panel page 7

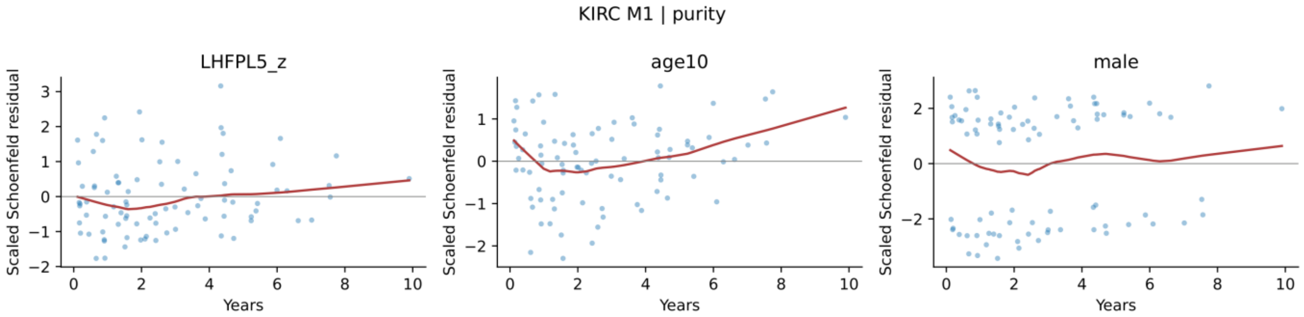

Continuation of Figure S9. See the legend on its first page.

Figure S9. Primary model proportional hazards diagnostics — panel page 8

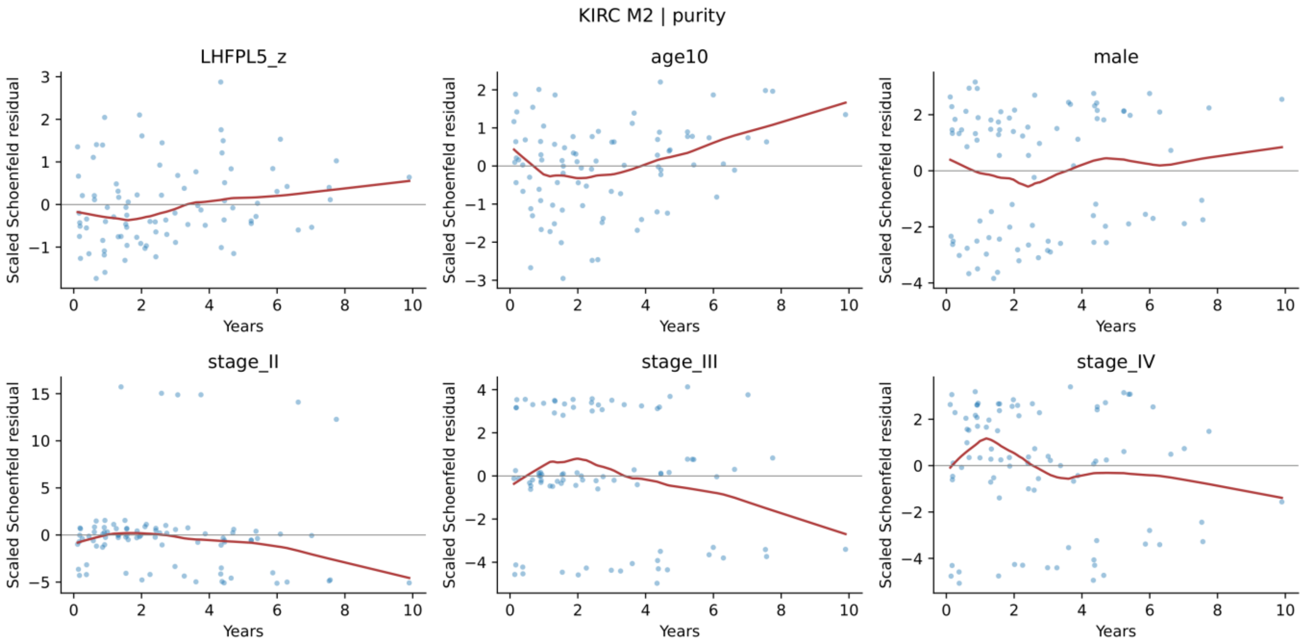

Continuation of Figure S9. See the legend on its first page.

Figure S9. Primary model proportional hazards diagnostics — panel page 9

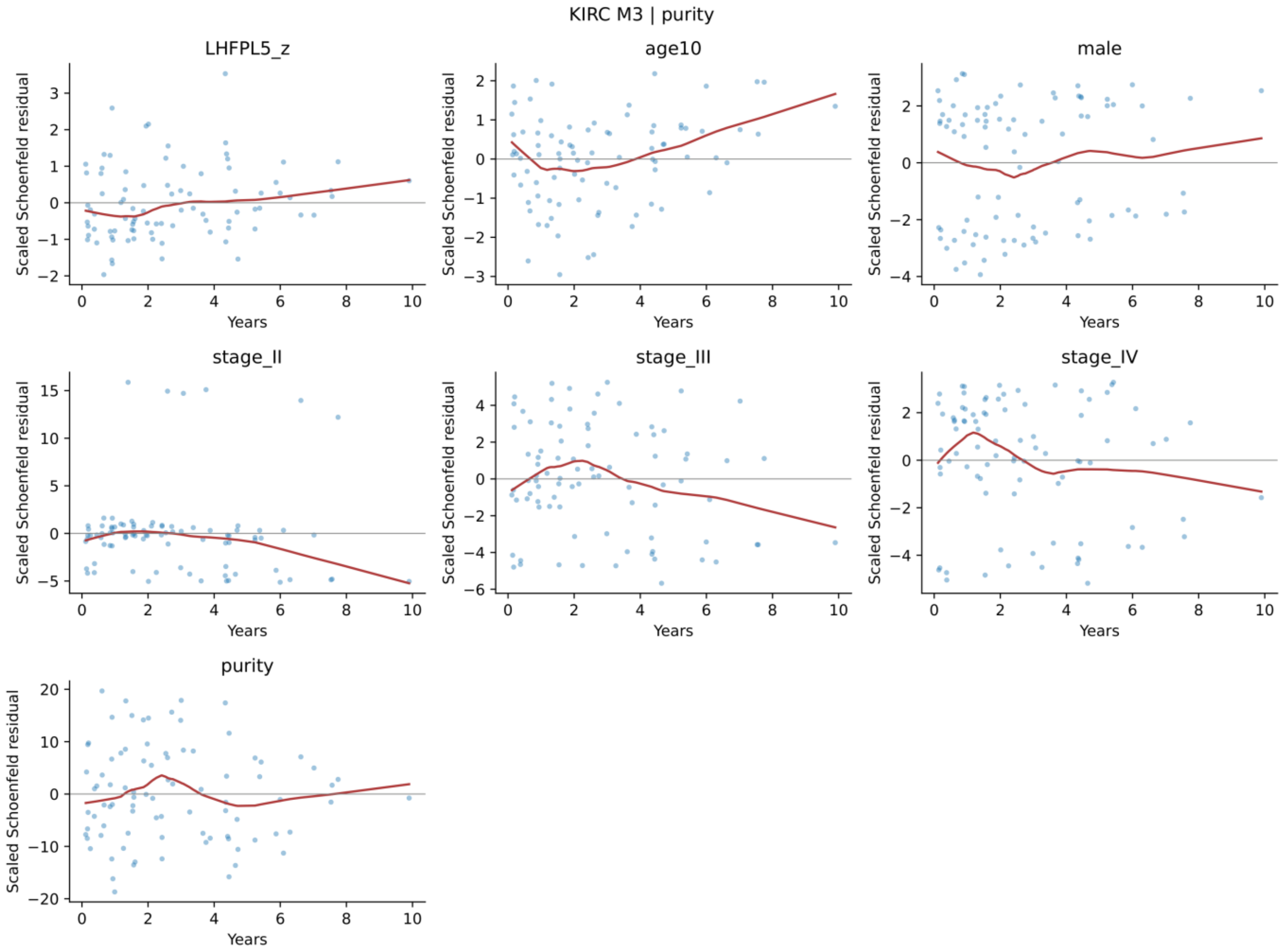

Continuation of Figure S9. See the legend on its first page.

Figure S9. Primary model proportional hazards diagnostics — panel page 10

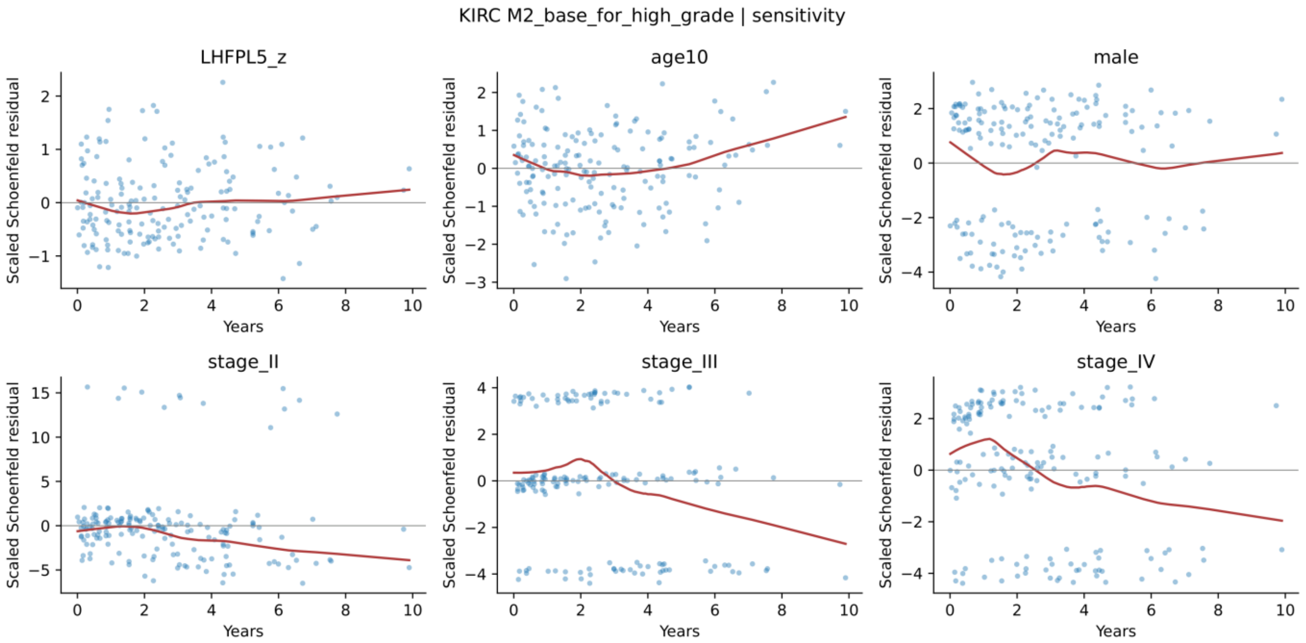

Continuation of Figure S9. See the legend on its first page.

Figure S9. Primary model proportional hazards diagnostics — panel page 11

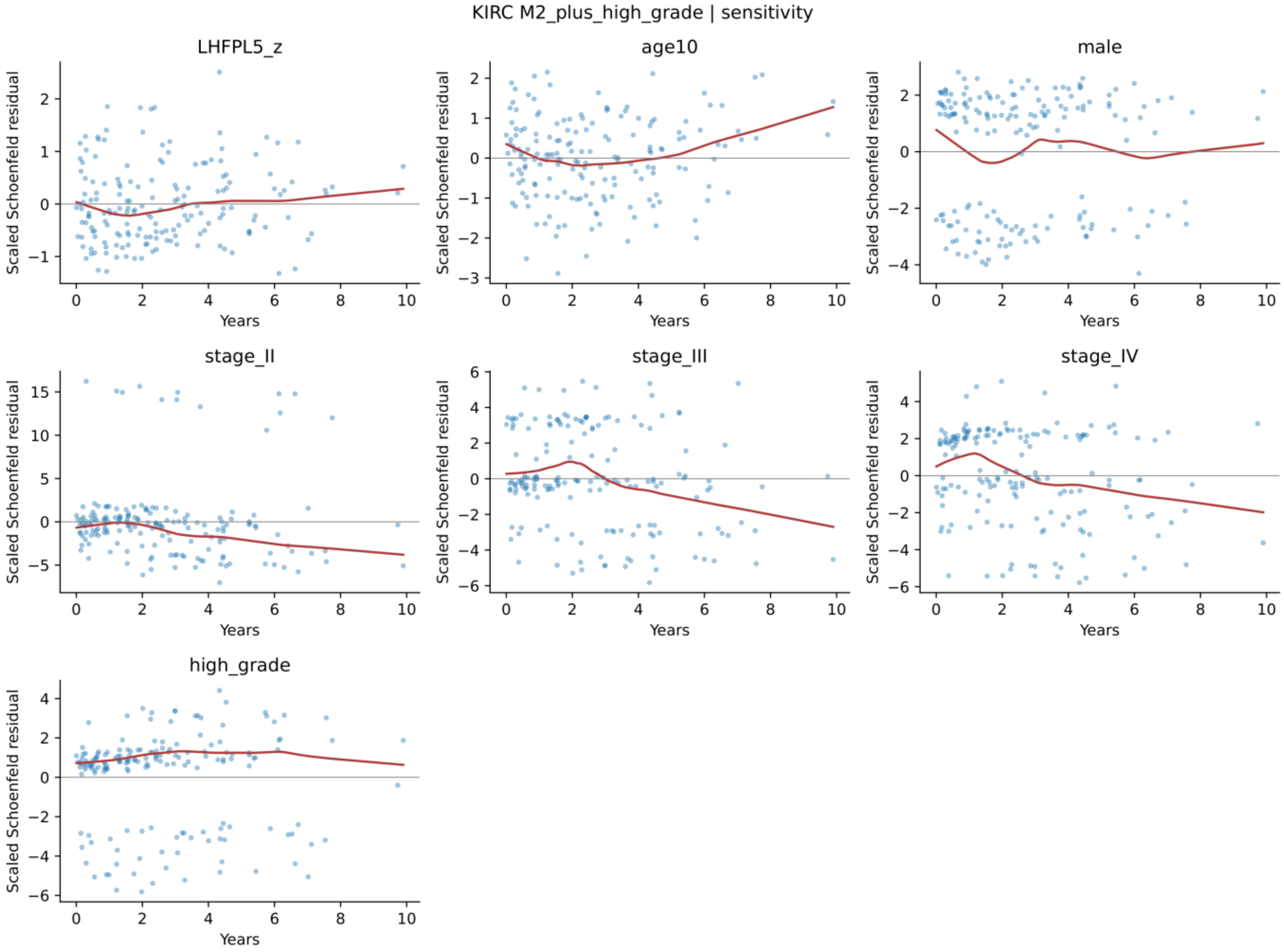

Continuation of Figure S9. See the legend on its first page.

Figure S9. Primary model proportional hazards diagnostics — panel page 12

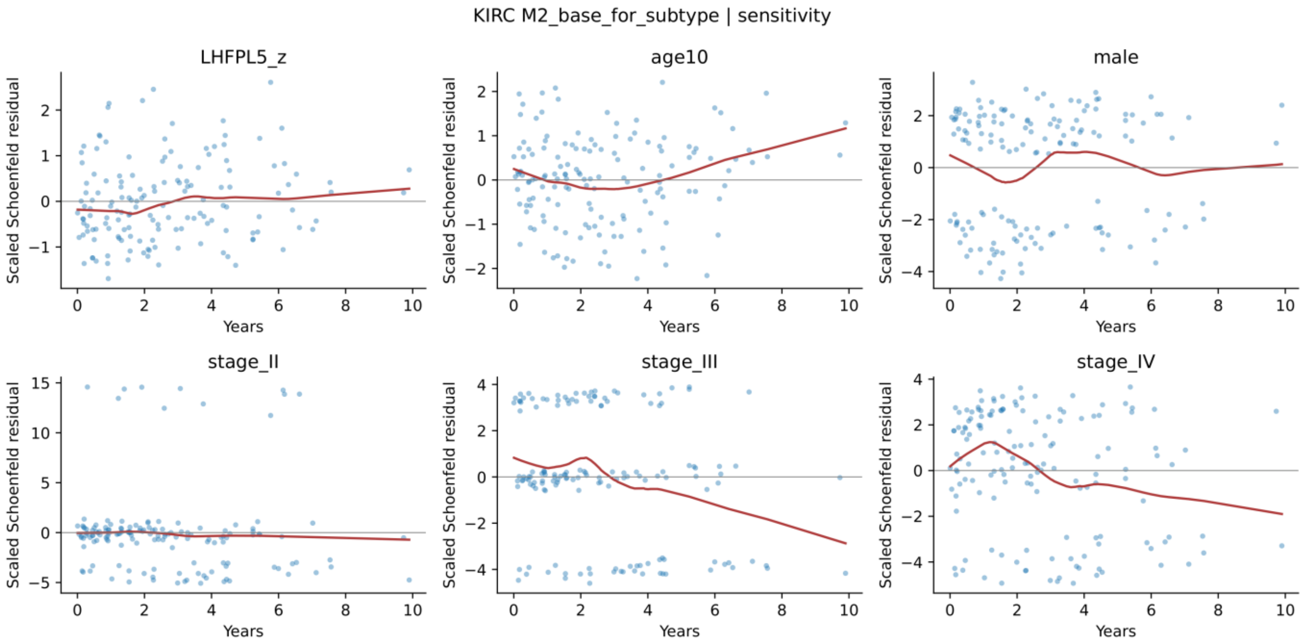

Continuation of Figure S9. See the legend on its first page.

Figure S9. Primary model proportional hazards diagnostics — panel page 13

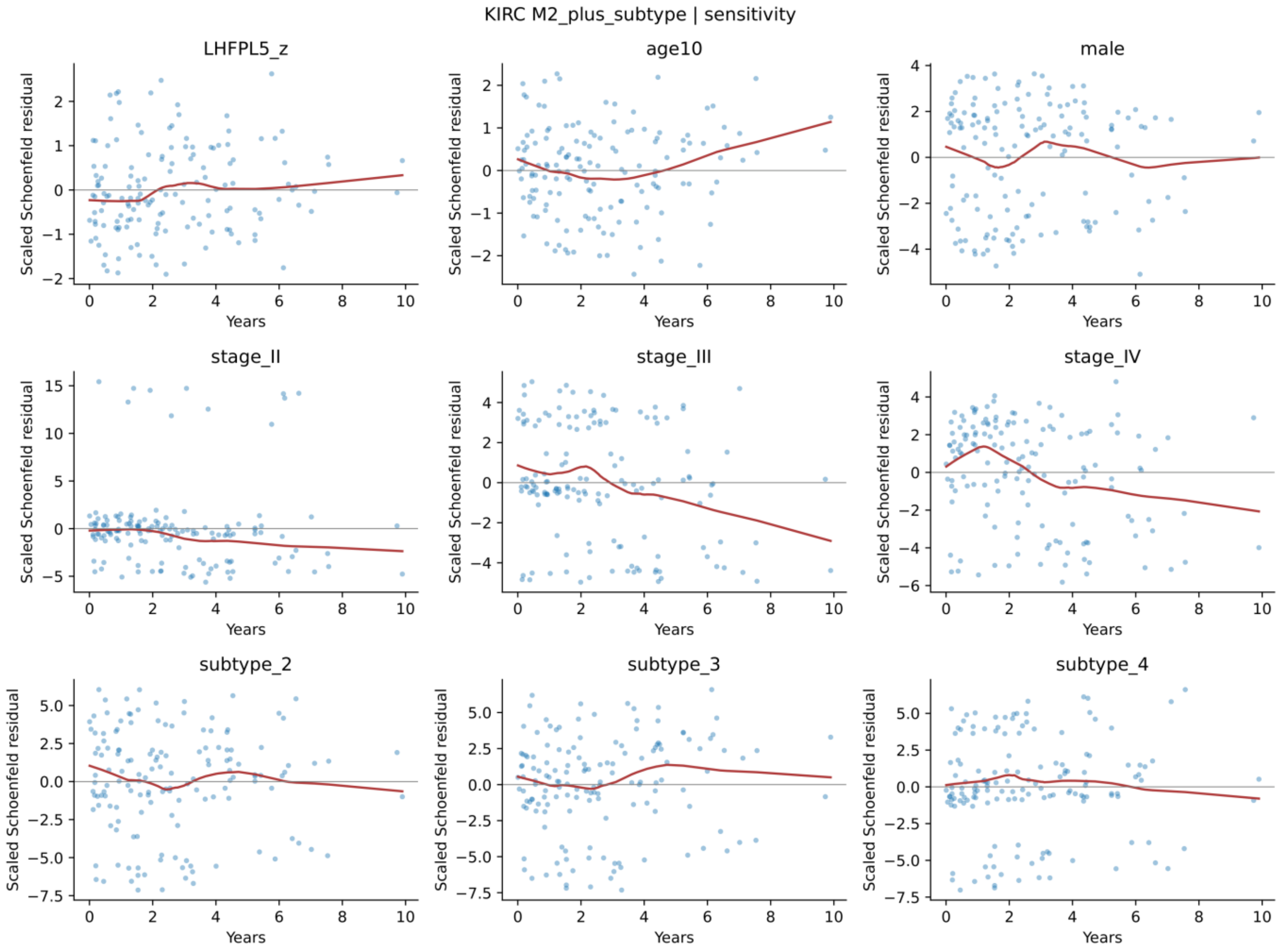

Continuation of Figure S9. See the legend on its first page.

Figure S9. Primary model proportional hazards diagnostics — panel page 14

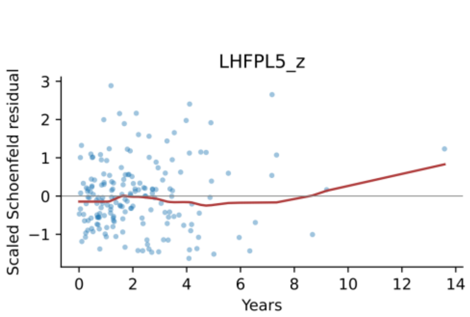

Continuation of Figure S9. See the legend on its first page.

Figure S9. Primary model proportional hazards diagnostics — panel page 15

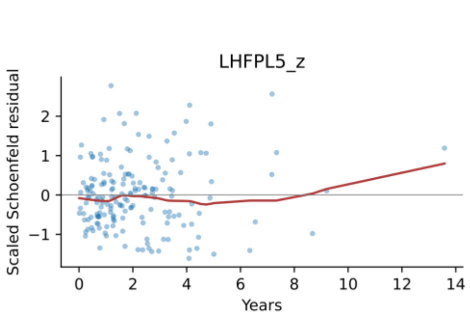

Continuation of Figure S9. See the legend on its first page.

Figure S9. Primary model proportional hazards diagnostics — panel page 16

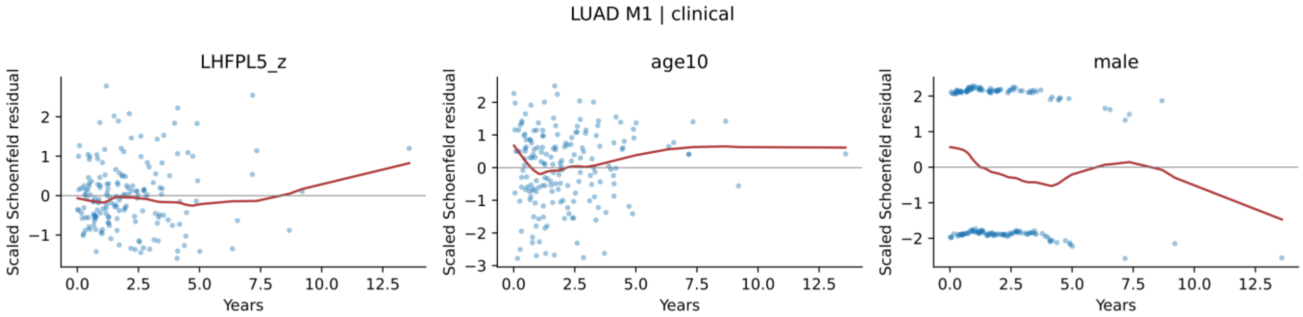

Continuation of Figure S9. See the legend on its first page.

Figure S9. Primary model proportional hazards diagnostics — panel page 17

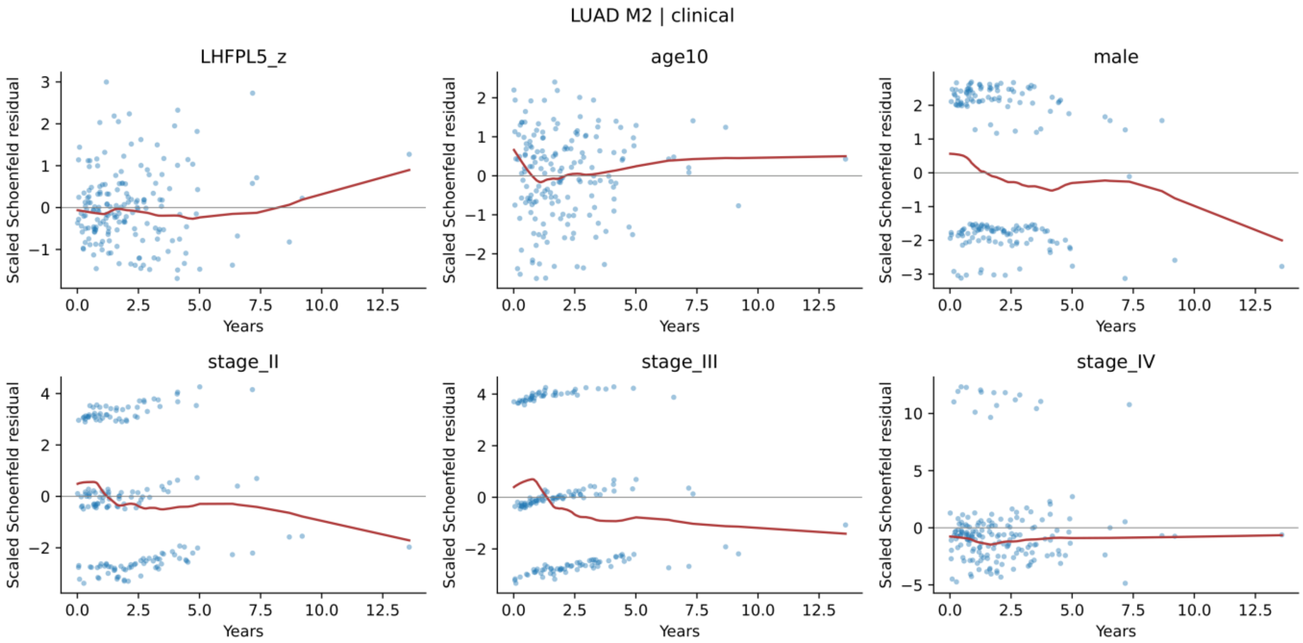

Continuation of Figure S9. See the legend on its first page.

Figure S9. Primary model proportional hazards diagnostics — panel page 18

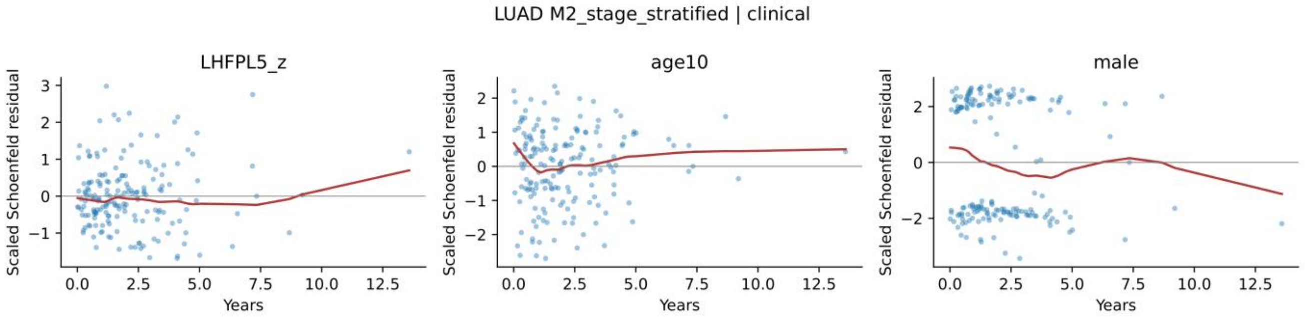

Continuation of Figure S9. See the legend on its first page.

Figure S9. Primary model proportional hazards diagnostics — panel page 19

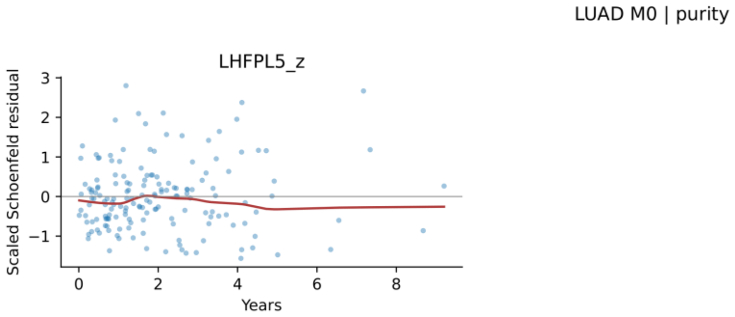

Continuation of Figure S9. See the legend on its first page.

Figure S9. Primary model proportional hazards diagnostics — panel page 20

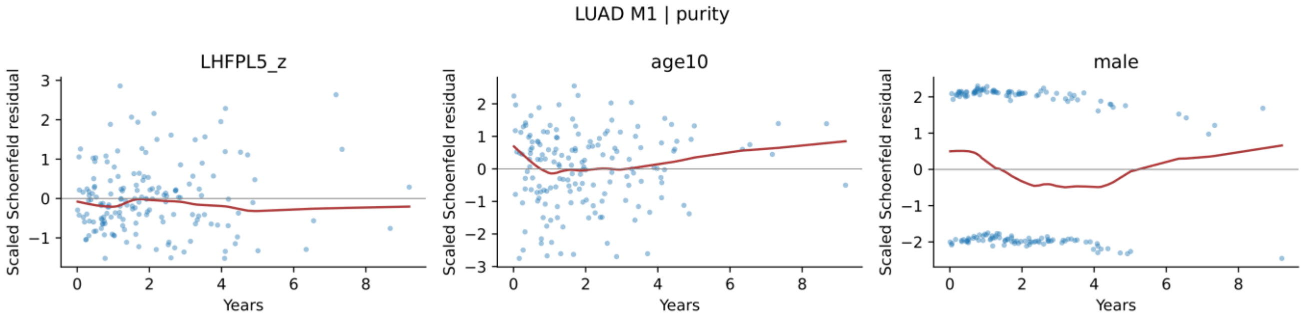

Continuation of Figure S9. See the legend on its first page.

Figure S9. Primary model proportional hazards diagnostics — panel page 21

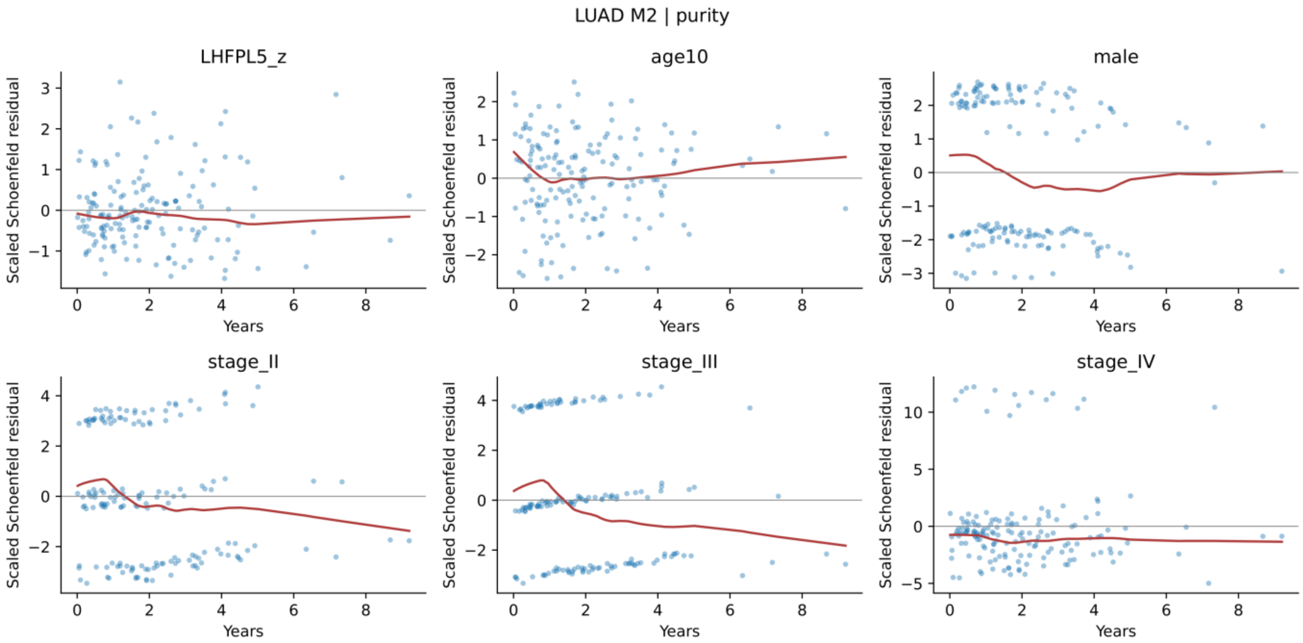

Continuation of Figure S9. See the legend on its first page.

Figure S9. Primary model proportional hazards diagnostics — panel page 22

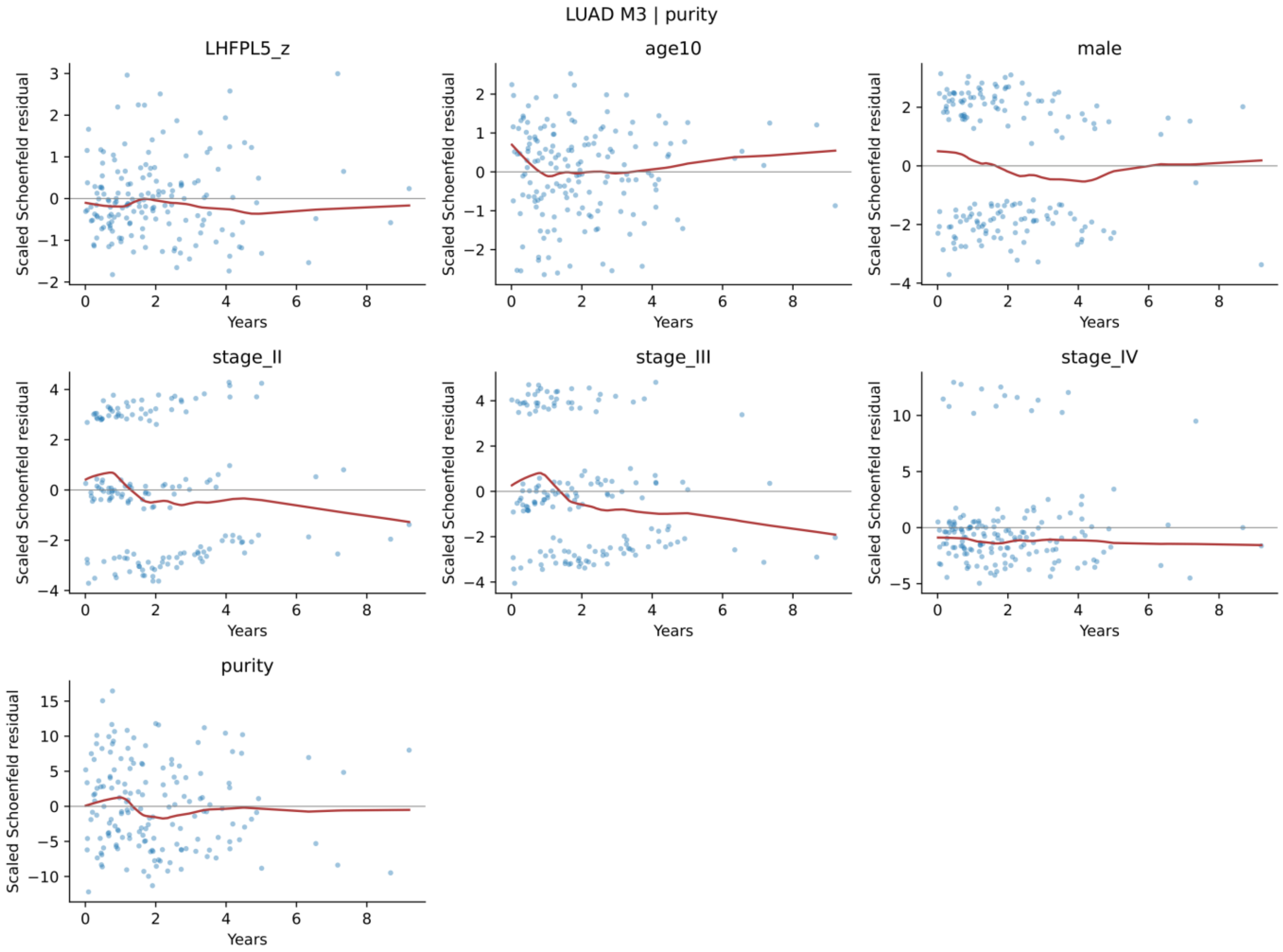

Continuation of Figure S9. See the legend on its first page.

Figure S9. Primary model proportional hazards diagnostics — panel page 23

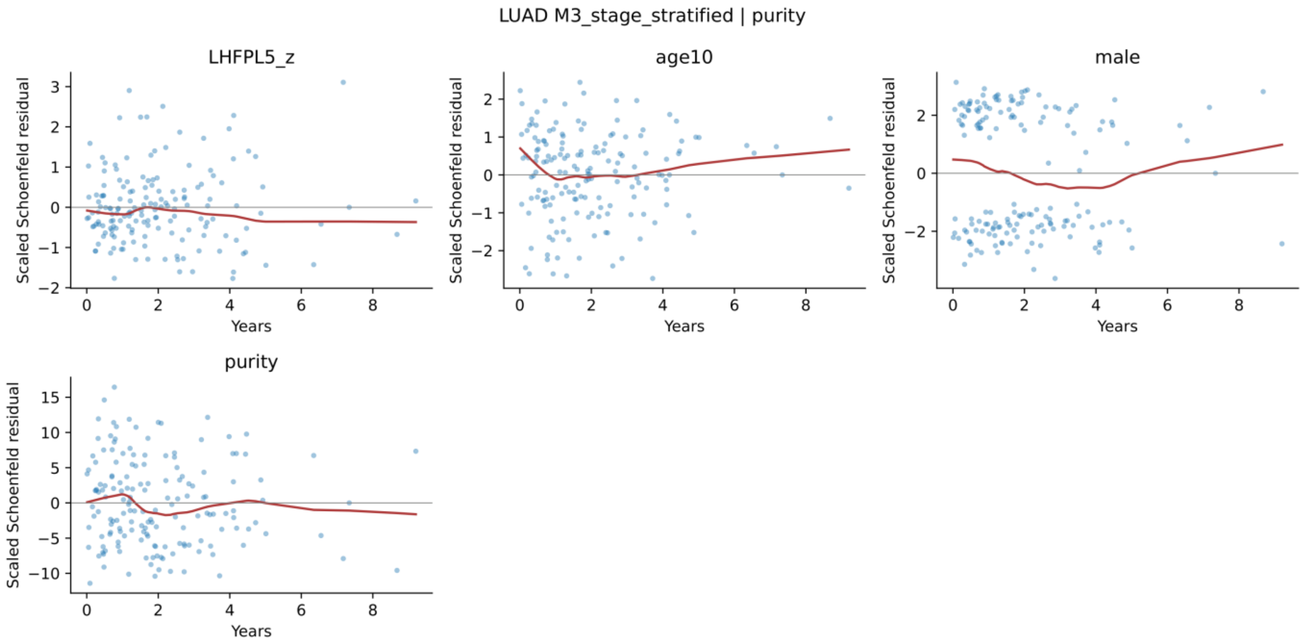

Continuation of Figure S9. See the legend on its first page.

Figure S9. Primary model proportional hazards diagnostics — panel page 24

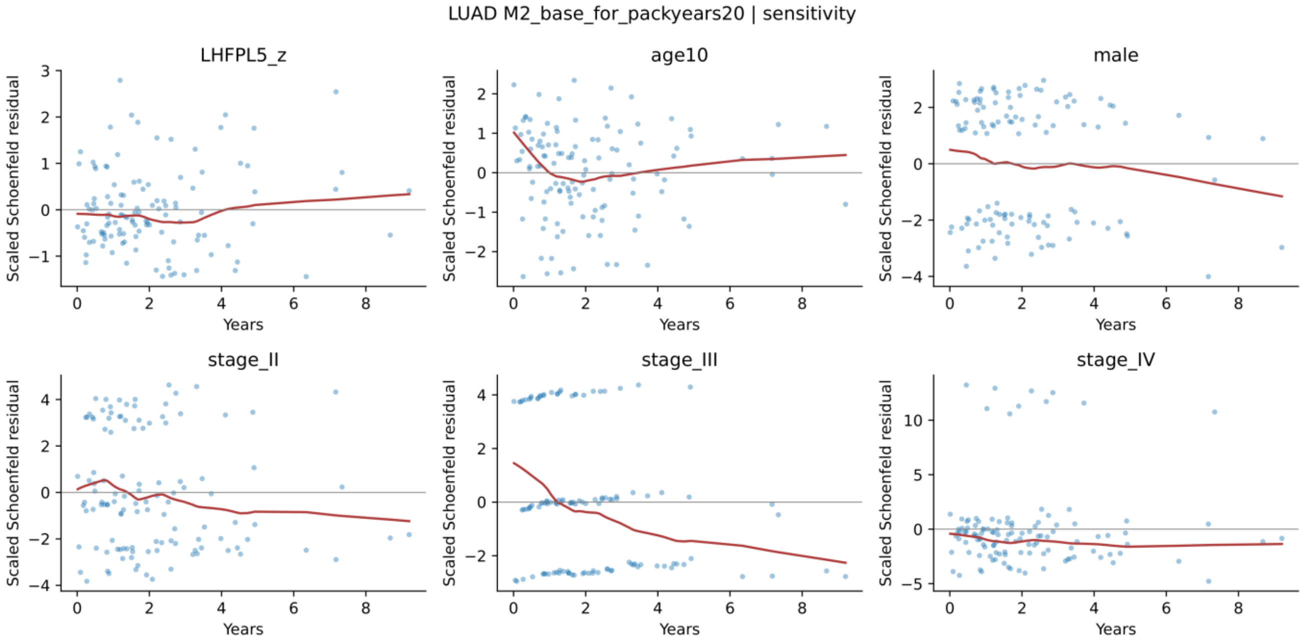

Continuation of Figure S9. See the legend on its first page.

Figure S9. Primary model proportional hazards diagnostics — panel page 25

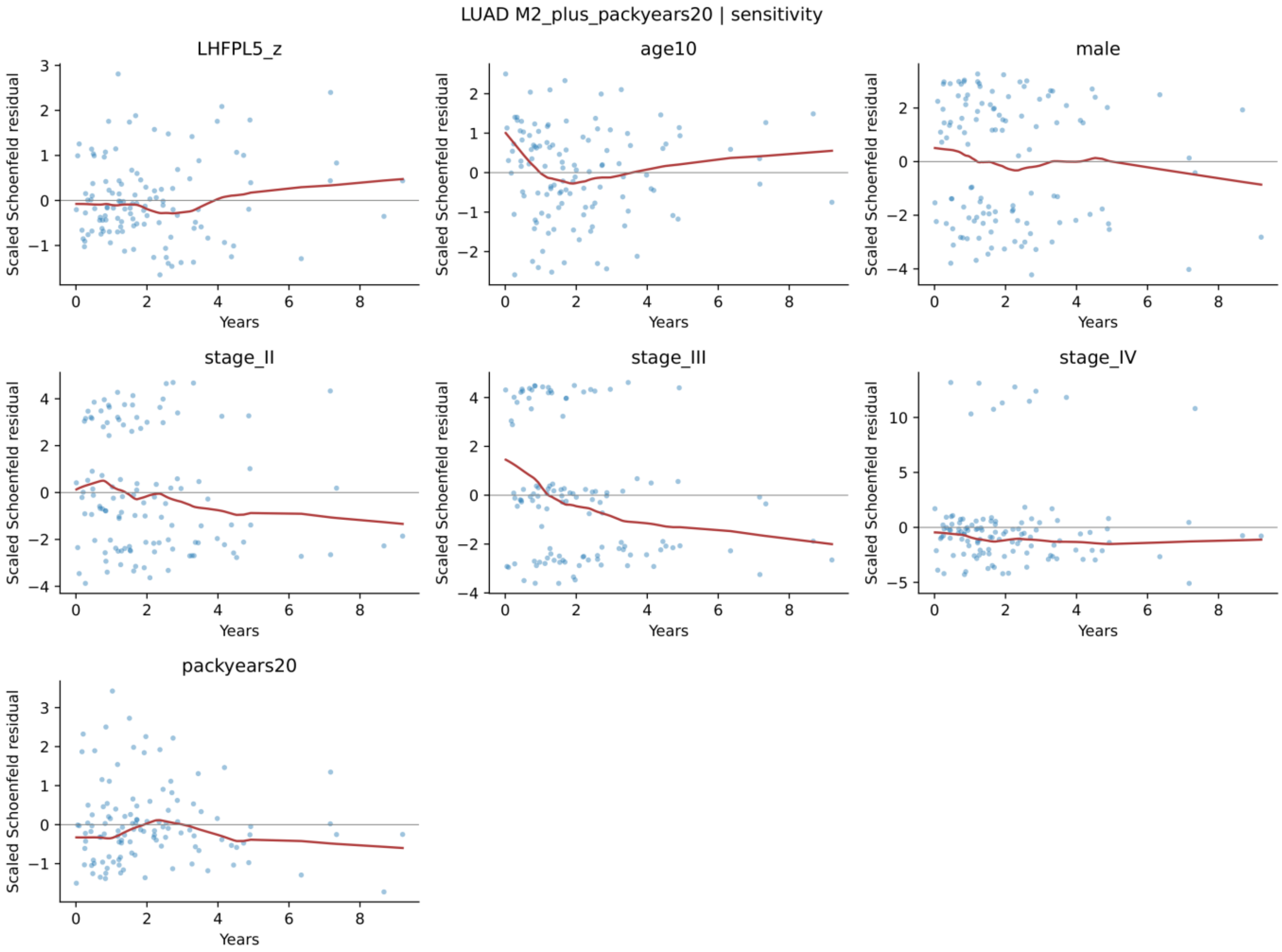

Continuation of Figure S9. See the legend on its first page.

Figure S10. Extended model proportional hazards diagnostics — panel page 1

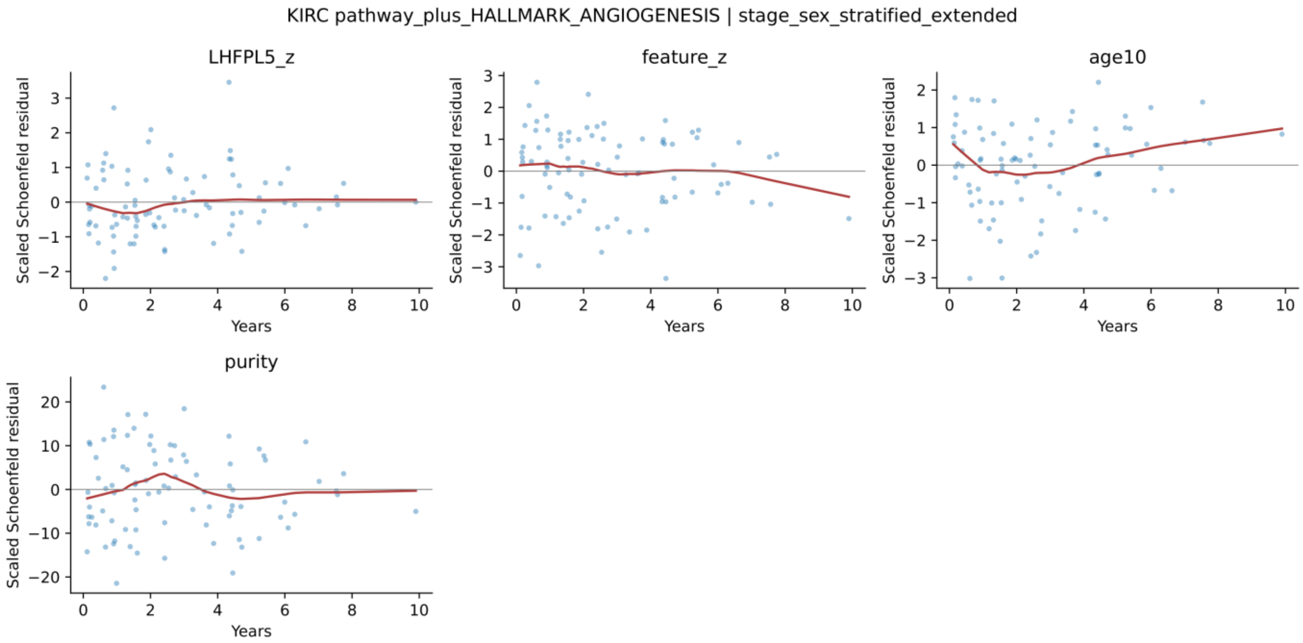

Diagnostic panels for selected pathway, immune, and methylation models. Panel titles identify models and cancers; corresponding coefficients, populations, and PH tests are in Tables S2 and S5–S7. Time-varying and stratified sensitivities are reported separately. Superseded external diagnostic panels were omitted; the latest external model diagnostics are in Table S10.

Figure S10. Extended model proportional hazards diagnostics — panel page 2

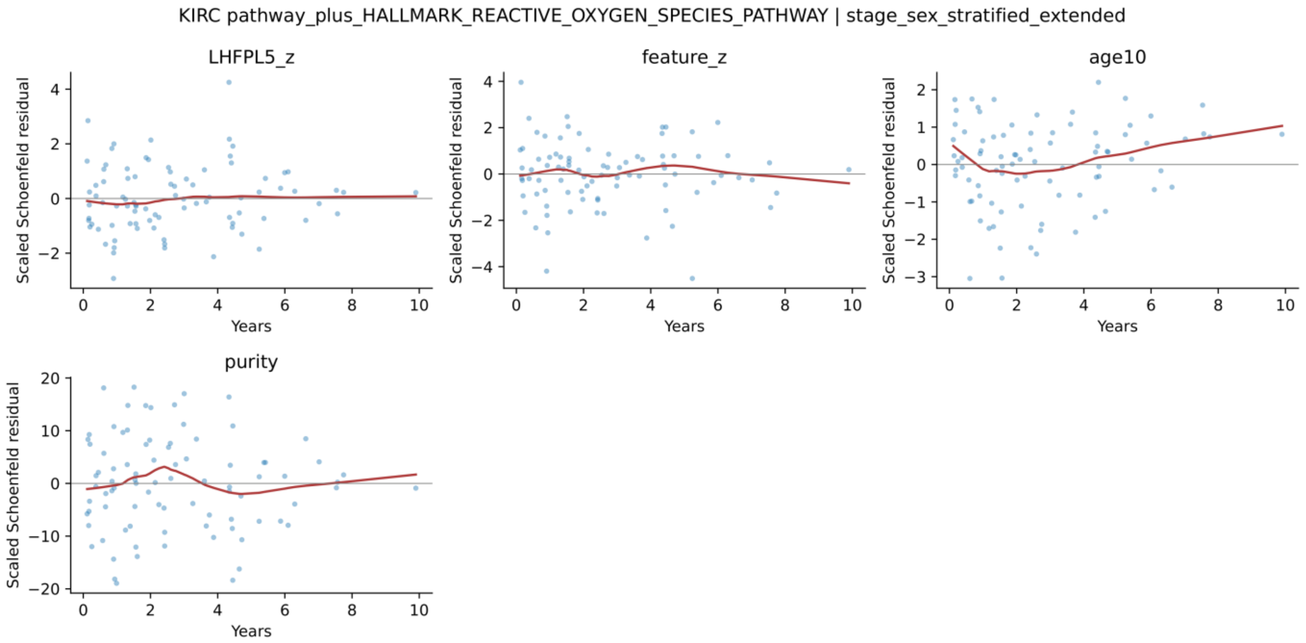

Continuation of Figure S10. See the legend on its first page.

Figure S10. Extended model proportional hazards diagnostics — panel page 3

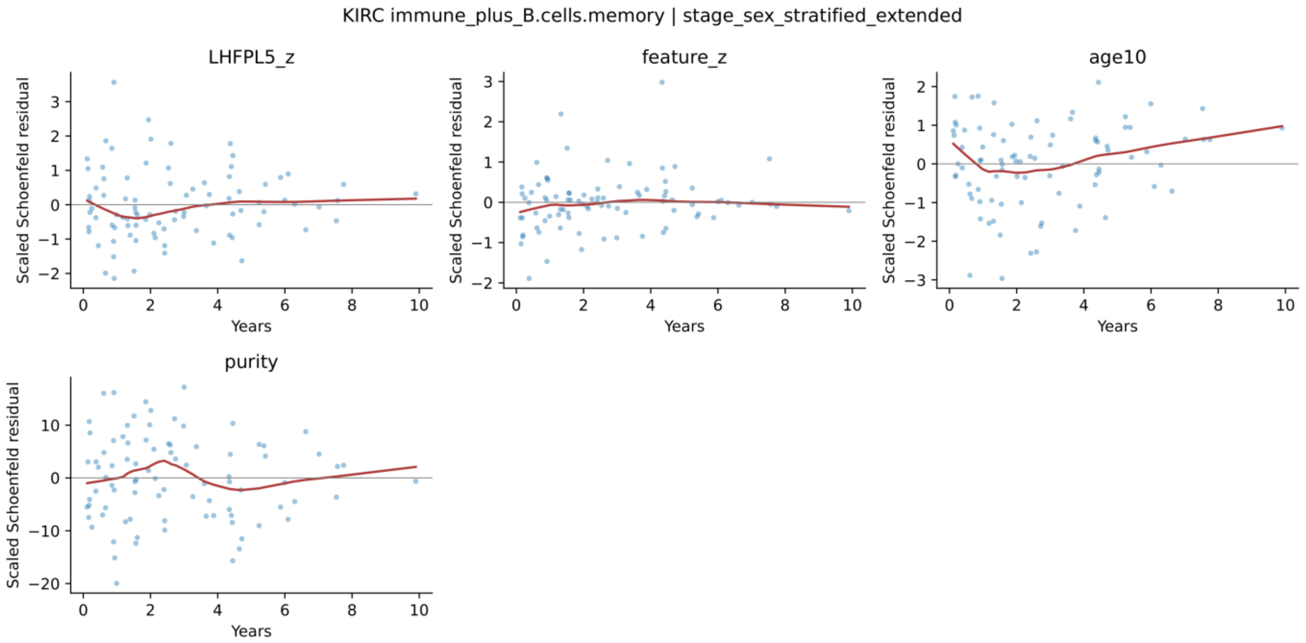

Continuation of Figure S10. See the legend on its first page.

Figure S10. Extended model proportional hazards diagnostics — panel page 4

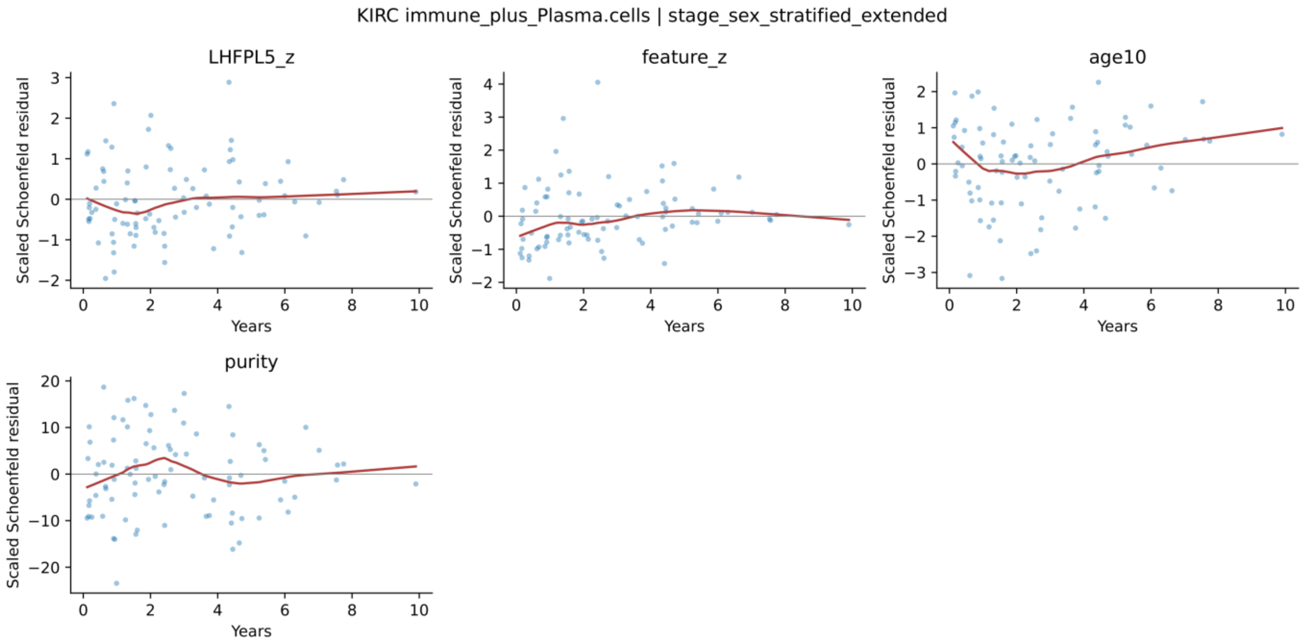

Continuation of Figure S10. See the legend on its first page.

Figure S10. Extended model proportional hazards diagnostics — panel page 5

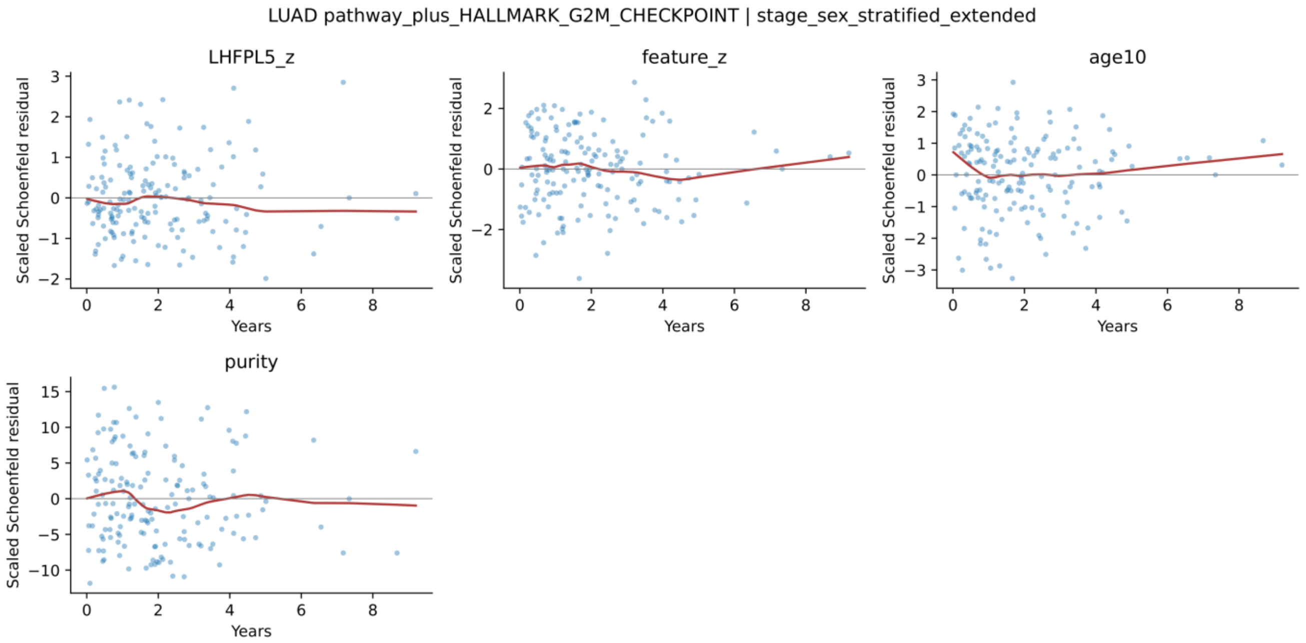

Continuation of Figure S10. See the legend on its first page.

Figure S10. Extended model proportional hazards diagnostics — panel page 6

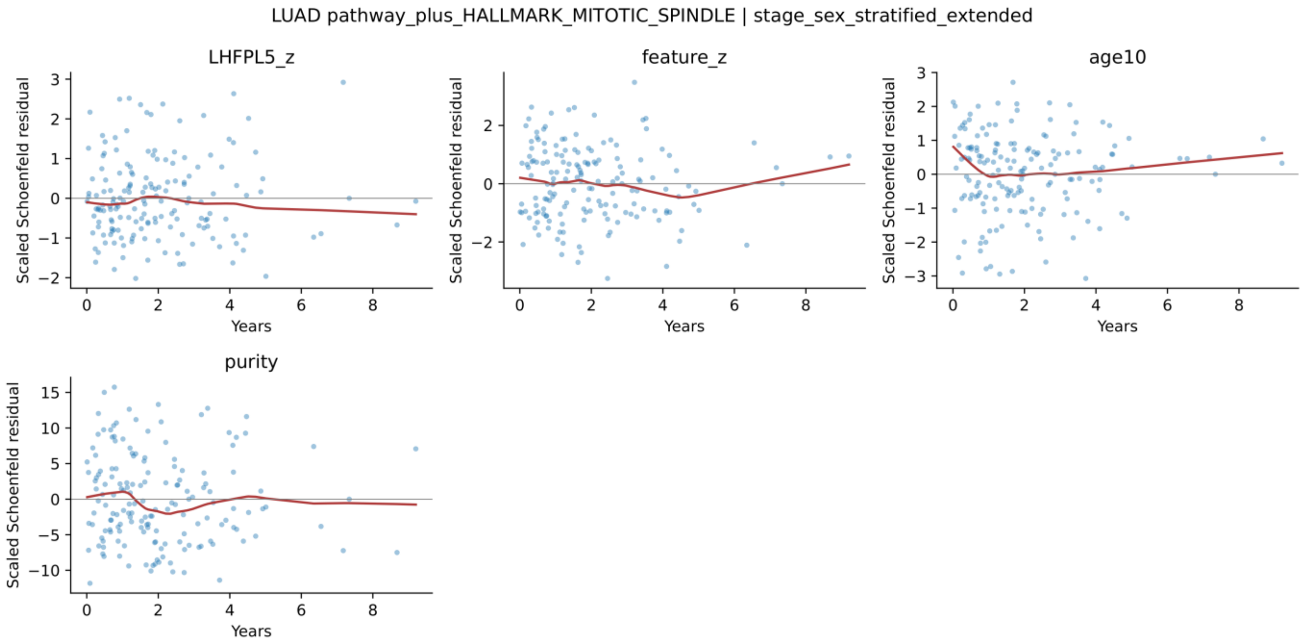

Continuation of Figure S10. See the legend on its first page.

Figure S10. Extended model proportional hazards diagnostics — panel page 7

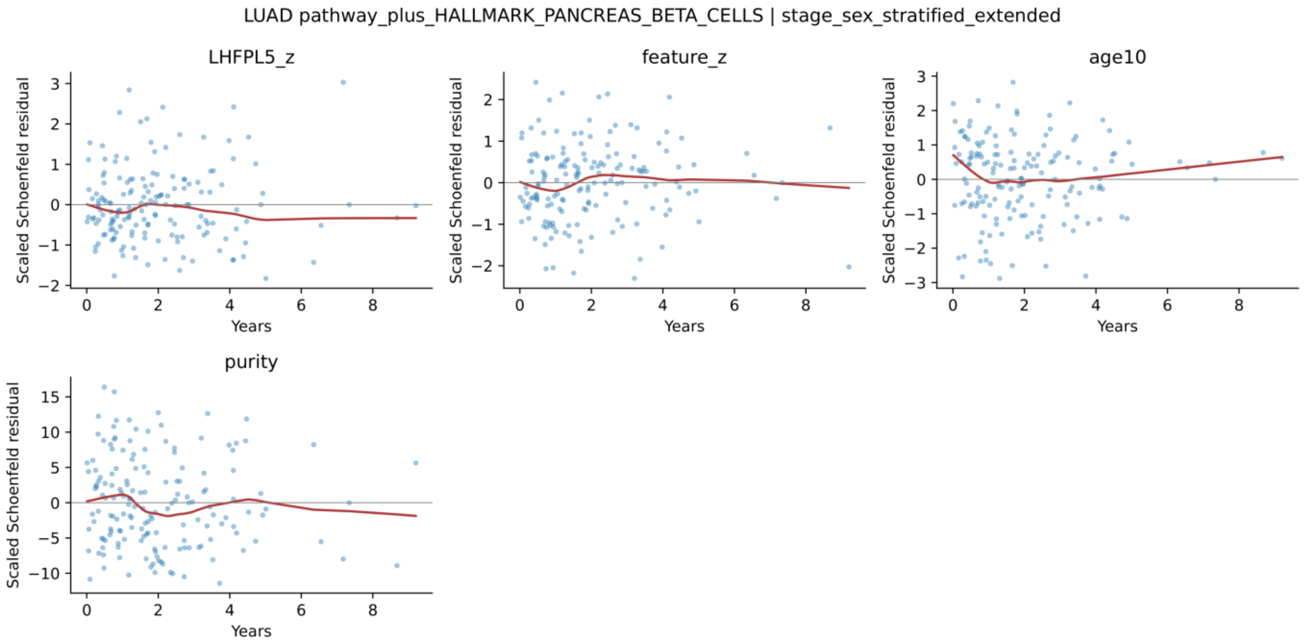

Continuation of Figure S10. See the legend on its first page.

Figure S10. Extended model proportional hazards diagnostics — panel page 8

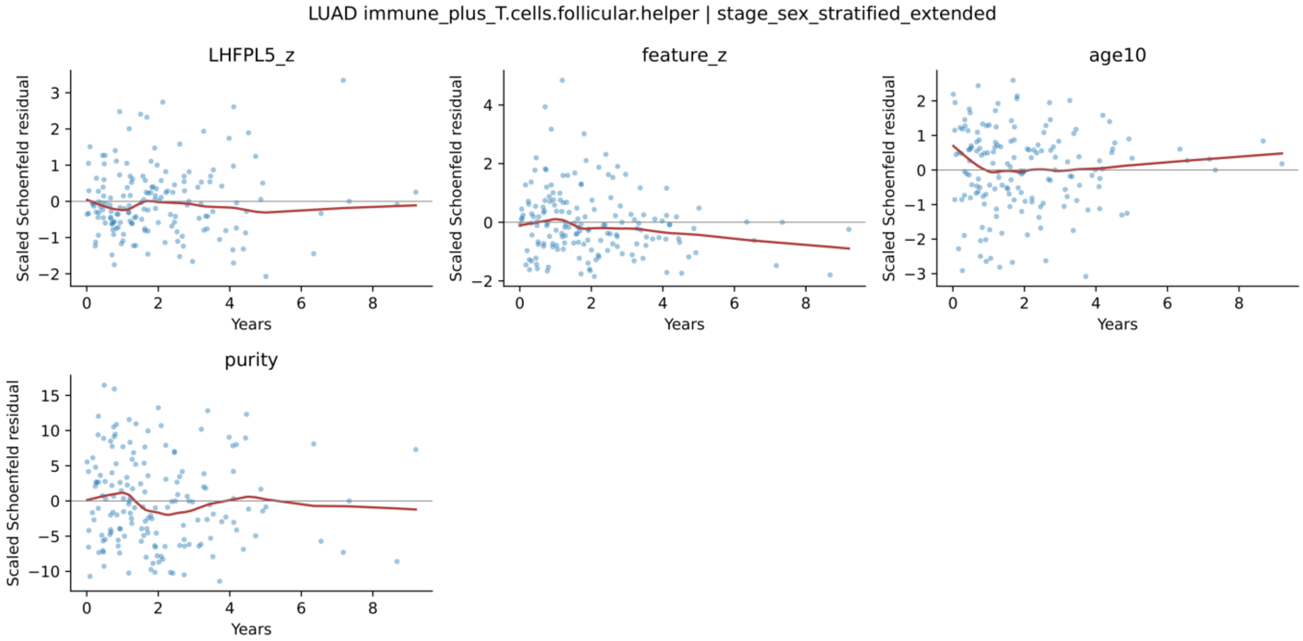

Continuation of Figure S10. See the legend on its first page.

Figure S10. Extended model proportional hazards diagnostics — panel page 9

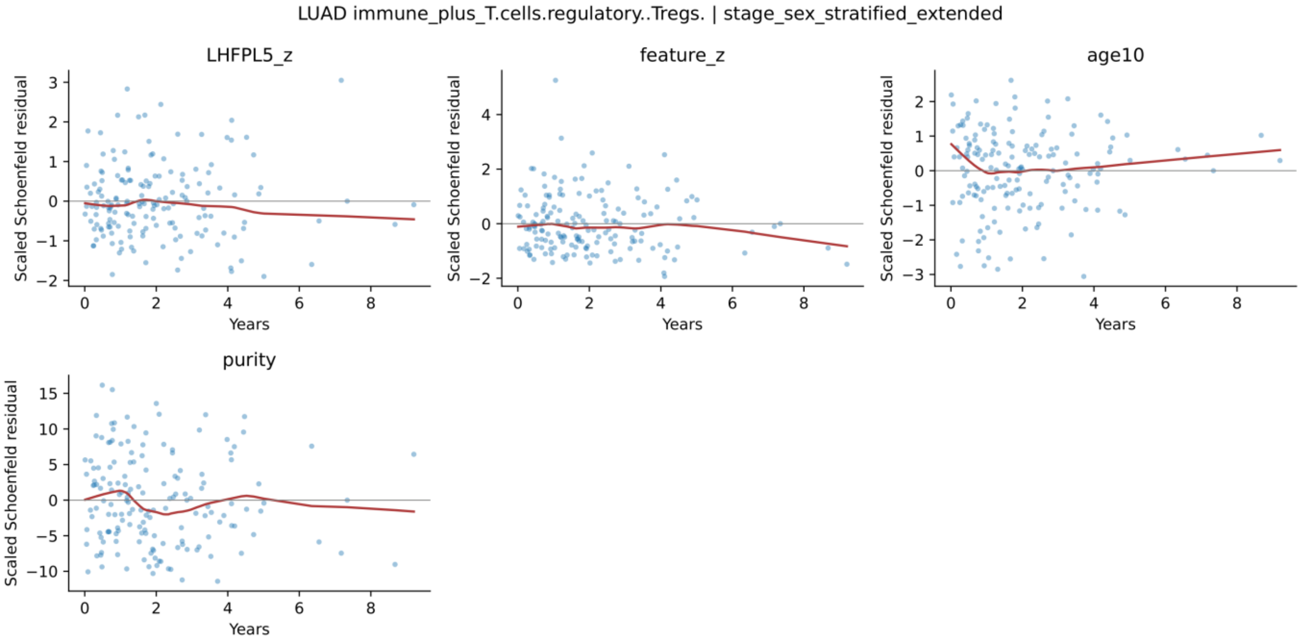

Continuation of Figure S10. See the legend on its first page.

Figure S10. Extended model proportional hazards diagnostics — panel page 10

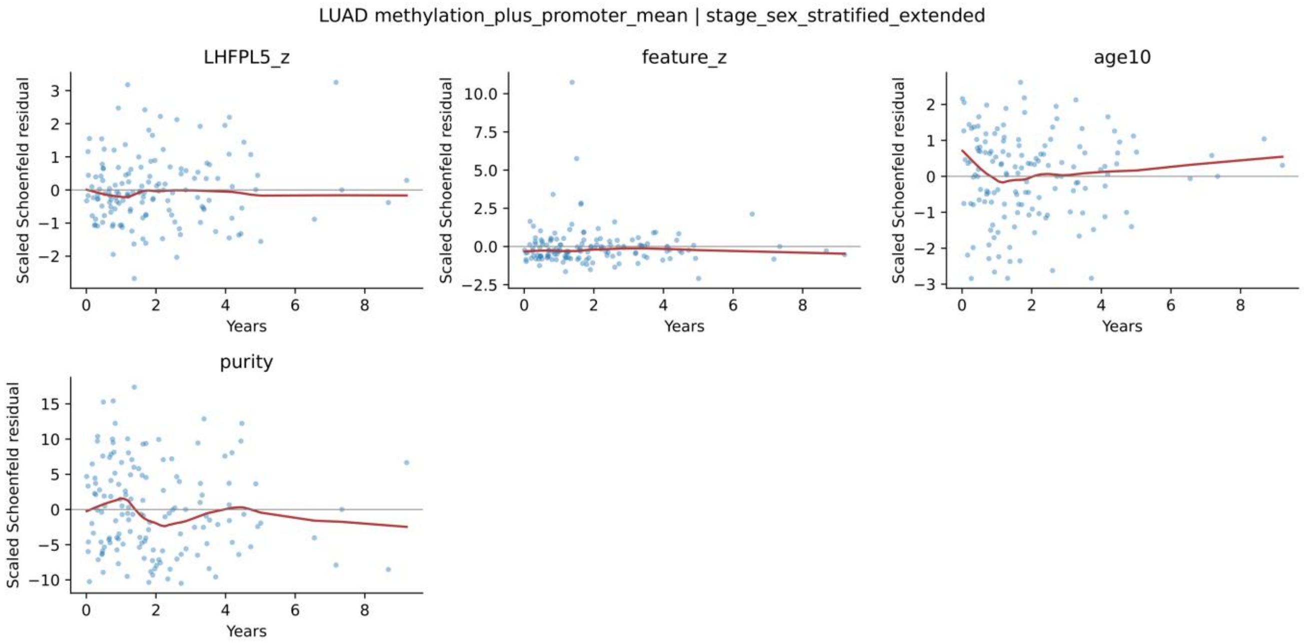

Continuation of Figure S10. See the legend on its first page.

Figure S10. Extended model proportional hazards diagnostics — panel page 11

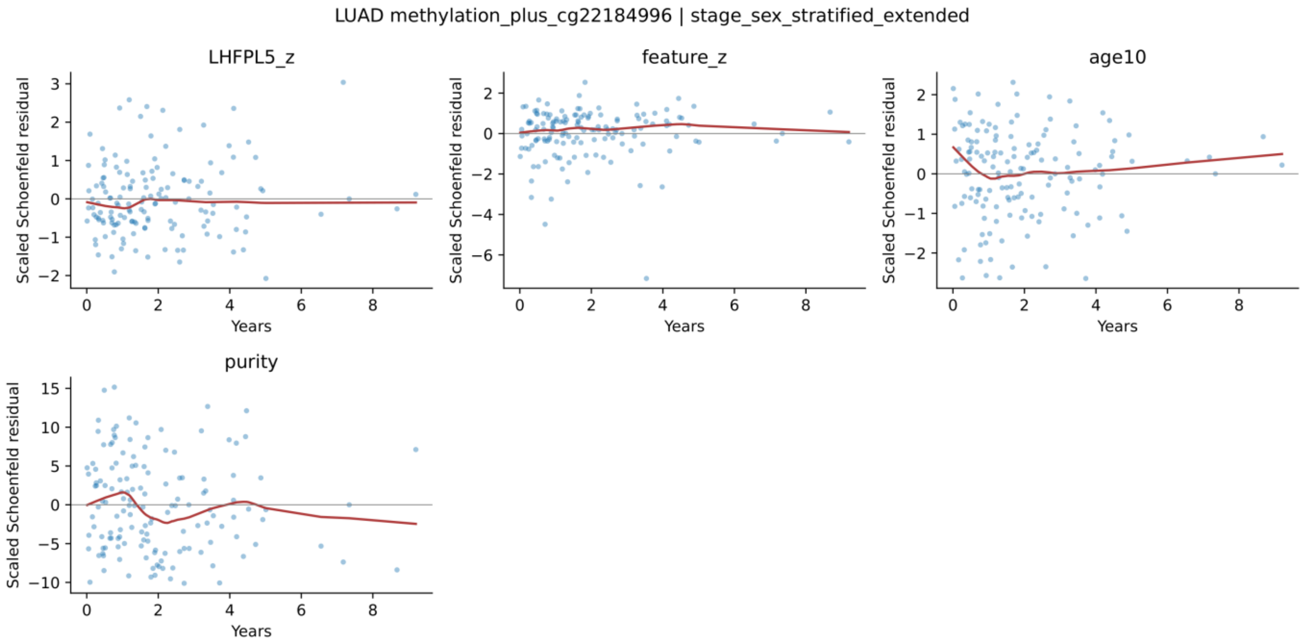

Continuation of Figure S10. See the legend on its first page.

Figure S11. Expression functional form sensitivity

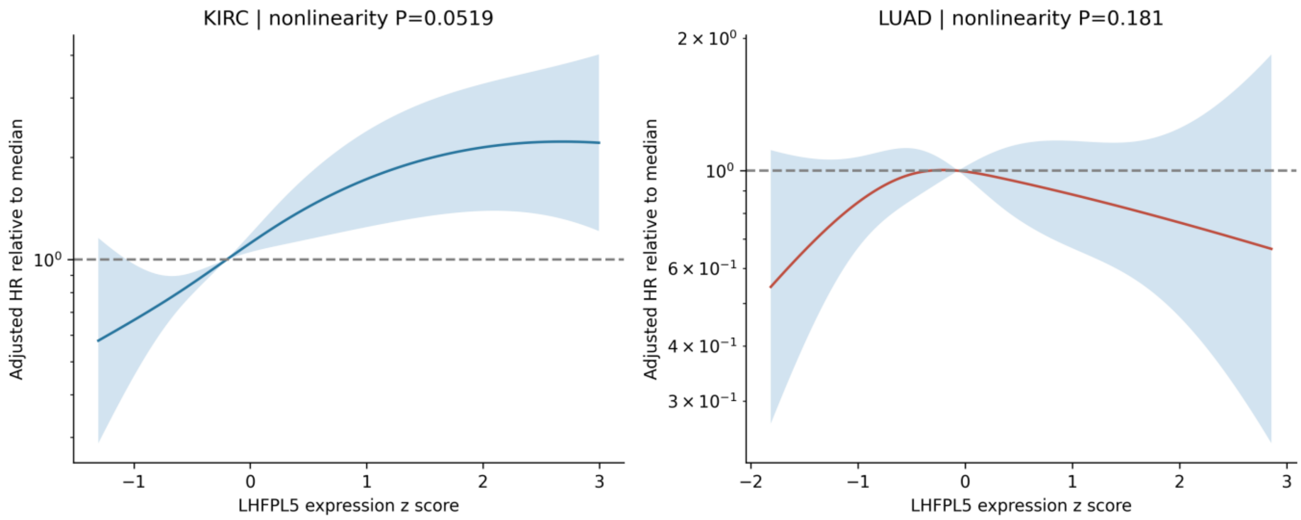

Natural cubic spline HR curves relative to median expression with three degrees of freedom, examined alongside quadratic terms. KIRC evidence depends on specification (quadratic P = 0.0152; spline nonlinearity P = 0.0519); LUAD spline P = 0.181. Full archived curves and tests are in Table S2.

Figure S12. Integrated expression model diagnostics

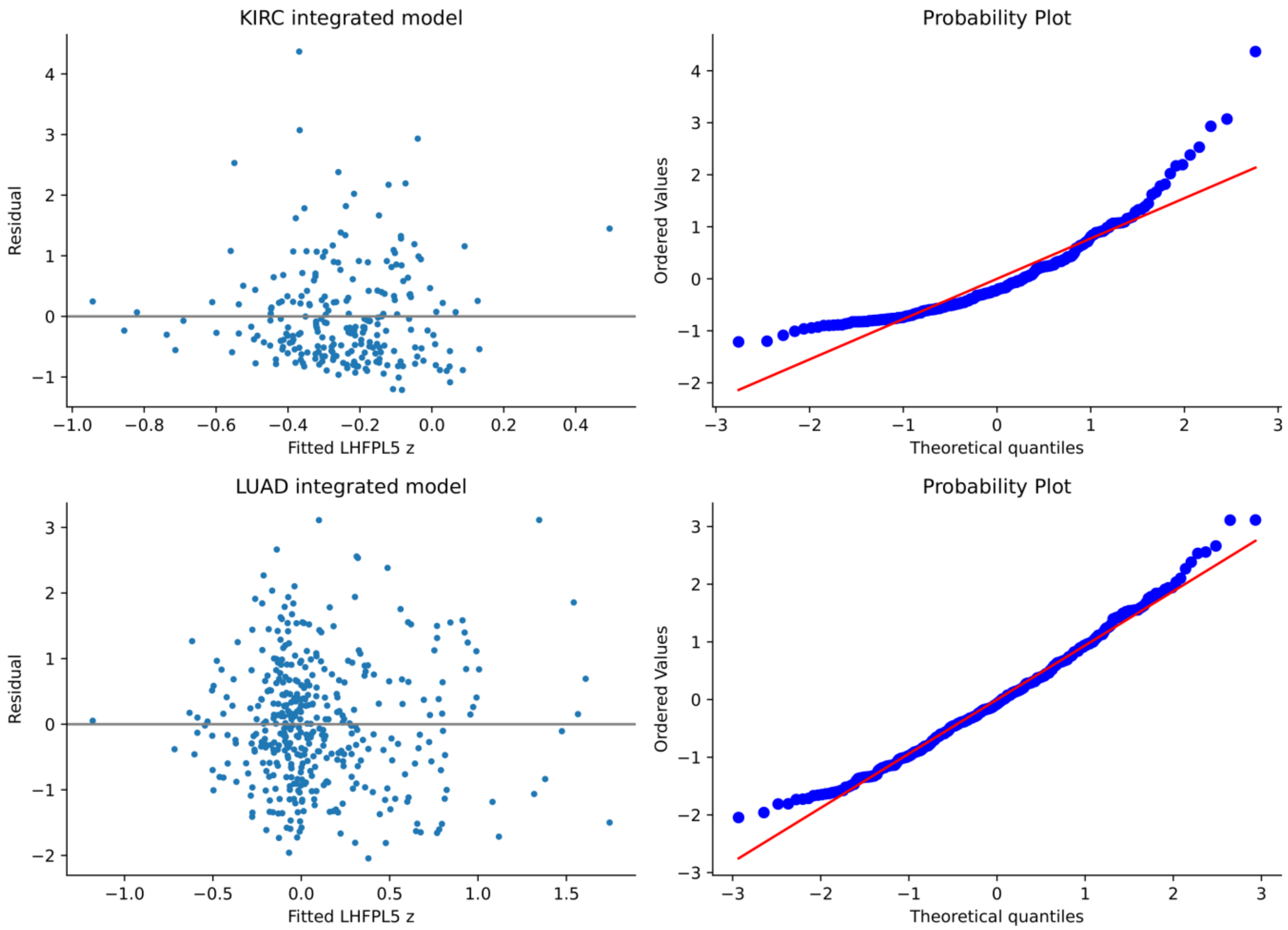

Residual and normal quantile plots for integrated expression models with methylation mean, copy ratio, purity, age, sex, and stage. HC3 uncertainty estimates were used. Table S8 includes heteroskedasticity, VIF, influence, and incremental model-fit outputs. These diagnostics limit interpretation of weak KIRC and heteroskedastic LUAD associations.
